# Disentangling the effects of multifunctional forestry practices on the abundances of birds and their invertebrate prey

**DOI:** 10.1002/eap.70198

**Published:** 2026-03-08

**Authors:** João Manuel Cordeiro Pereira, Sara Klingenfuß, Marco Basile, Julian Frey, Grzegorz Mikusiński, Ilse Storch

**Affiliations:** ^1^ Chair of Wildlife Ecology and Management University of Freiburg Freiburg Germany; ^2^ Chair of Geobotany University of Freiburg Freiburg Germany; ^3^ Swiss Federal Research Institute WSL Birmensdorf Switzerland; ^4^ Chair of Remote Sensing and Landscape Information Systems (FELIS), University of Freiburg Freiburg Germany; ^5^ School for Forest Management, Swedish University of Agricultural Sciences (SLU) Skinskatteberg Sweden; ^6^ Mammal Research Institute, Polish Academy of Sciences Białowieża Poland; ^7^ Present address: Department of Forest Nature Conservation Forest Research Institute of Baden‐Württemberg (FVA) Freiburg Germany; ^8^ Present address: Chair of Forest Growth and Dendroecology University of Freiburg Freiburg Germany

**Keywords:** Central Europe, forest arthropods, forest birds, integrative forest management, montane forests, path analysis, resource availability, retention forestry

## Abstract

European forests are increasingly managed to harmonize production goals with biodiversity conservation, through practices such as retention and close‐to‐nature forestry. Forest birds may benefit from these practices, but it remains unclear how the effects of different management practices compare, and whether responses to management are driven by changes in the availability of invertebrates, a crucial element of bird diets during the breeding season. To answer these questions, we carried out bird point counts on 135 1‐ha plots in southwestern Germany from 2017 to 2022, and measured the abundance of invertebrate groups in the lower forest strata using flight interception traps and pitfall traps. We used *N*‐mixture models and Bayesian generalized linear models (GLMs) to estimate, respectively, how abundances of 32 bird species and 20 invertebrate groups respond to predictors representing forest management, structure, composition, and the abiotic environment. We then compared the responses of birds and invertebrates, and employed piecewise structural equation models (SEMs) to disentangle the causal links between forest structure and abundances of bird guilds and invertebrate groups. Bird abundances responded to predictors representing retention and close‐to‐nature forestry practices, but the direction of effects varied across species and facets of management. Moreover, the effects of retention practices were weaker than those of close‐to‐nature practices, especially those of admixing broadleaf trees. Hence, these management practices likely need to be applied in tandem with others (e.g., gap creation) to secure a diverse forest bird assemblage. Invertebrate abundances responded to both management types, but responses did not clearly align with those of bird species, and SEMs did not support direct links between bird and invertebrate abundances. Still, we revealed parallel positive responses of birds and invertebrate groups to the same habitat features, such as broadleaf share, suggesting that these may function as cues for high food availability during habitat selection by birds. Therefore, forest management that aims at increasing bird populations should address other potential limiting factors, such as nest site availability, in addition to fostering high invertebrate abundances, which may safeguard habitat quality for birds.

## INTRODUCTION

In Europe, centuries of forest clearing, followed by the development of industrialized forestry, have led to widespread loss of old‐growth forests (Sabatini et al., 2018) and habitat homogenization (Bengtsson et al., 2000). This has had detrimental effects on multiple forest‐dwelling taxa, including birds, especially those reliant on features underrepresented in production forests, such as multilayered stands, large trees, and deadwood (Lehikoinen & Virkkala, 2018; Moning & Müller, 2009; Nascimbene et al., 2013). Nowadays, European forests are increasingly managed to reconcile timber production goals with biodiversity protection, following a multifunctional forestry paradigm (European Commission, 2021). In Central Europe, even‐aged monocultures of Norway spruce (*Picea abies*) have given way since the early 1990s to forests managed under close‐to‐nature forestry (Bauhus et al., 2013; Bürgi, 2015), that is, by admixing broadleaf trees to diversify forest composition, and employing single‐tree selection and natural regeneration, thereby ensuring continuous forest cover and uneven‐aged stands. In the past decade, retention forestry—entailing retention of dead and living trees across harvesting cycles—has also been adopted throughout Central Europe (Gustafsson et al., 2020). Different management systems have different effects on forest organisms, and these effects may also vary substantially across taxa (Elek et al., 2018; Paillet et al., 2010). Still, retention and close‐to‐nature forestry practices have proven beneficial for multiple taxa linked with late‐successional stages, deadwood and native broadleaf trees (e.g., Edelmann et al., 2022; Nascimbene et al., 2013; Tomao et al., 2020). Moreover, it is thought that the shift to multifunctional forestry has helped stabilize or even reverse population declines of forest birds throughout Central Europe (EBCC/BirdLife/RSPB/CSO, 2024; Reif et al., 2022; Schulze et al., 2019; Storch et al., 2023).

The literature examining responses of birds to forest management has typically focused on measuring species richness, abundance, and assemblage composition (e.g., Akresh et al., 2023; Basile et al., 2019; Vanderwel et al., 2007). Still, abundance patterns do not necessarily reflect habitat quality (Johnson, 2007), and studies have much less often delved into the ecological mechanisms driving abundance responses (Belder et al., 2018). One such driver is the availability of food resources, which may act as a limiting factor for bird breeding success (Ruffino et al., 2014; Seress et al., 2020) and population growth (Tallamy & Shriver, 2021; Watson, 2015). The majority of bird species inhabiting temperate forests rely on invertebrates as food during the breeding season (Nyffeler et al., 2018). Invertebrate abundance and activity are also important predictors of functional diversity in forest bird assemblages (Barbaro et al., 2019). In turn, forest structure can affect both birds and invertebrates (e.g., Moorman et al., 2012; Renner et al., 2024; Vélová et al., 2021; Vergara et al., 2021). For instance, forest gaps were linked with higher abundances of both foliage‐dwelling arthropods and foliage‐gleaning birds in the southeastern USA (Moorman et al., 2012). In Central Europe, Renner et al. (2024) found that invertebrate biomass partly mediates the effects of forest structure on overall bird abundance. Hence, the effects of multifunctional forestry practices on bird abundance may potentially be mediated by changes in the invertebrate supply.

Close‐to‐nature forestry fosters the vertical heterogeneity and tree diversity of stands, both well‐established drivers of bird abundance (James & Wamer, 1982; MacArthur & MacArthur, 1961). In Central Europe, multilayered forests show higher abundance and diversity of birds (Heidrich et al., 2020; Lewandowski et al., 2021; Schall et al., 2018), and host higher abundance of foliage arthropods (Knuff et al., 2020; Wildermuth et al., 2024), compared to even‐aged stands. On the other hand, close‐to‐nature practices lead to wide extents of closed‐canopy forests (Gustafsson et al., 2020), which host more specialized communities, but lower abundance and diversity of birds and invertebrates (Černecká et al., 2020; Lewandowski et al., 2021; Penone et al., 2019; Przepióra et al., 2020). As for tree species composition, a majority of foliage‐gleaning bird species in Central Europe favor foraging in broadleaf trees over conifers during the breeding season (Felton et al., 2010), likely due to higher abundances of defoliating insects (Ampoorter et al., 2020). However, Norway spruce canopies host higher spider abundances (Wildermuth et al., 2023) and European beech (*Fagus sylvatica*) is considered insect‐poor in comparison with other broadleaved trees (Catfolis et al., 2023; Korňan & Adamík, 2017). Regarding retention practices, these are considered beneficial for cavity‐nesting birds (Basile et al., 2019; Rosenvald & Lõhmus, 2008), which are typically limited in production forests by the low availability of nesting sites (Remm & Lõhmus, 2011; Wesołowski & Martin, 2018). However, benefits of retention may not be limited to nest site provision. Forests with larger diameter trees tend to show higher overall bird abundances (Penone et al., 2019; Rosenvald et al., 2011), and older forests show higher invertebrate abundances (e.g., Knuff et al., 2020; Lange et al., 2014; Leidinger et al., 2019). An increased volume of deadwood can sustain higher abundances of saproxylic insects (e.g., Eckerter et al., 2021; Gossner et al., 2013), while lying deadwood can also increase the abundance of non‐saproxylic invertebrates (Kirchenbaur et al., 2017; Seibold et al., 2016). In summary, forest birds consume a wide breadth of invertebrate taxa with correspondingly diverse ecological requirements and responses to management. Thus, there is a need to better discern how bird species and their prey respond to different facets of management practices. It can also be anticipated that effects of retention are weaker than those of close‐to‐nature forestry, given that the former is more recent, and forests can bear legacies of past management (Munteanu et al., 2016).

A growing body of literature (reviewed by van Klink et al., 2020) has uncovered declines in insect abundance and biomass in the temperate zone, extending into forest habitats (Staab et al., 2023). Managing Central European forests for a more diverse tree composition and higher structural complexity is expected to buffer against insect declines (Staab et al., 2023), with potential benefits for forest birds. Still, only a few studies (e.g., Renner et al., 2024; Vélová et al., 2021) have so far assessed how abundances of both forest birds and their invertebrate prey respond to the management practices currently applied in Central European forests. Moreover, to our knowledge, none of the existing studies have differentiated the effects of management practices across multiple bird foraging guilds and a wide range of potential prey groups, or focused on mixed beech–spruce–fir montane forests, which cover wide swathes of this region (Leuschner & Ellenberg, 2017).

In this study, we measure the abundances of bird species and a range of invertebrate taxa potentially preyed upon by birds, as well as multiple variables representing management practices and other facets of the forest environment, in a montane forest region of Central Europe. We investigate whether: (1) forest maturity variables, as a proxy for retention practices, affect the abundance of bird species across nesting and foraging guilds; (2) close‐to‐nature forestry shows more pronounced effects on bird and invertebrate abundances than the more recently implemented retention practices; (3) both retention and close‐to‐nature forestry practices affect the abundance of invertebrates available to ground‐foraging, foliage‐gleaning, and bark‐foraging birds; and (4) the abundance of bird guilds responds to increased invertebrate availability, suggesting trophic‐mediated effects of management. Overall, we expect the direction of effects to vary across taxa and across individual predictors, including those reflecting the same management approach (e.g., share of broadleaf trees and canopy cover, that both reflect close‐to‐nature forestry). By answering these questions, we contribute to a more mechanistic, resource‐based understanding of the effects of multifunctional forestry on birds, which can then inform future practice.

## METHODS

### Study area

**FIGURE 1:**
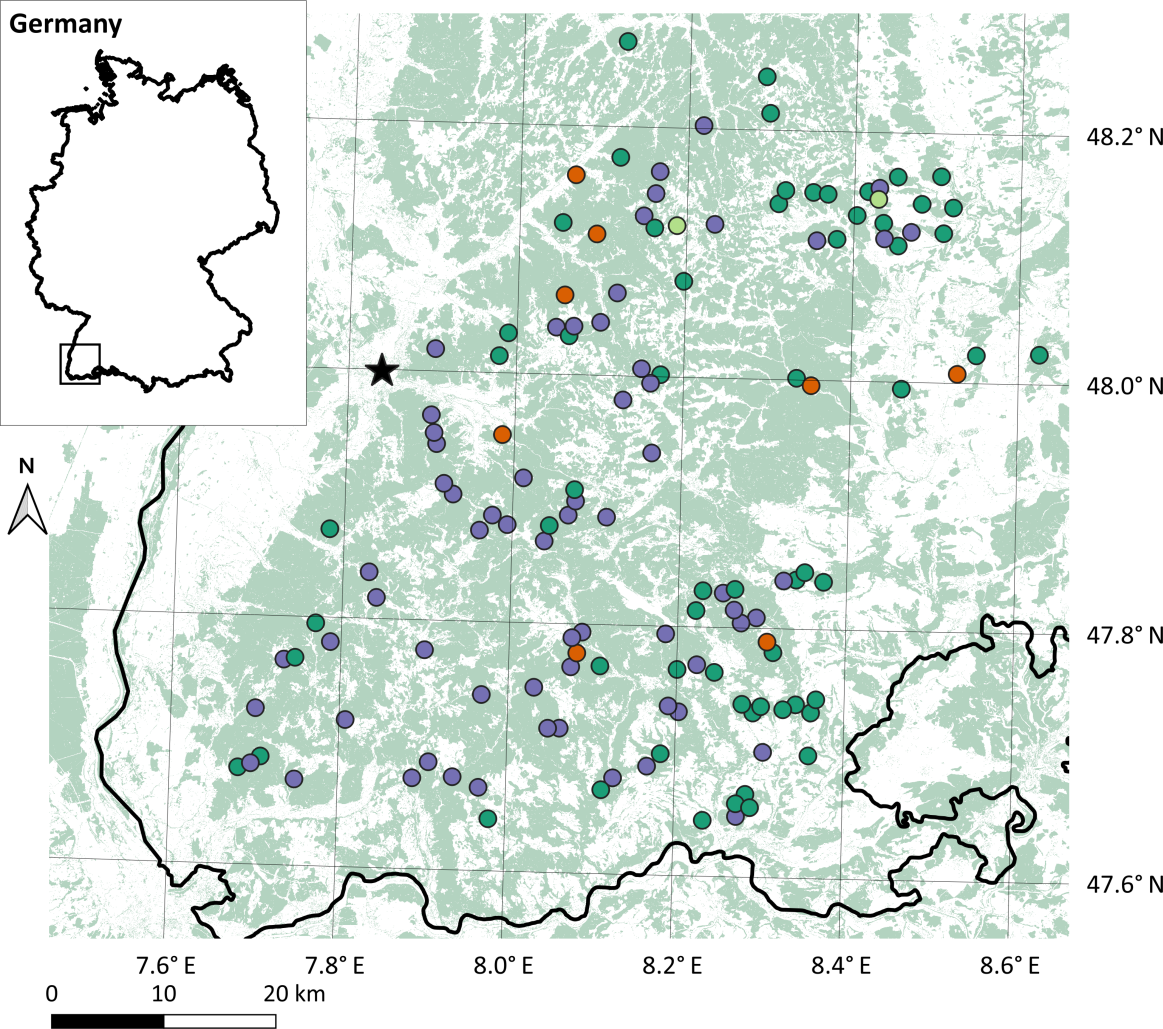
Map of the study area, with circles denoting the 135 ConFoBi plots, and forest cover in the background. Plot color represents the availability of invertebrate data: Purple plots have both pitfall and flight interception trap data, light green only the former, dark green only the latter, and orange neither type. Bird abundance data are available from all plots. The inset shows the location of the study area within Germany. The largest city in the area, Freiburg, is marked with a star.

We conducted this study in 135 1‐ha plots established by the ConFoBi Research Training Group (Storch et al., 2020) in the Black Forest, southwestern Germany (Figure 1). The Black Forest is a low‐elevation mountain range (1493 m) covered by mixed montane forests, dominated by Norway spruce, European beech, and silver fir (*Abies alba*). Plots were selected following a stratified design that covered gradients of forest structural complexity and surrounding forest cover, and were spaced by >750 m (details in Storch et al., 2020). All plots were located in state‐owned forests, managed under a close‐to‐nature forestry paradigm since the early 1990s, and in which retention of deadwood and habitat trees has gradually been implemented since 2010 (“Alt‐ und Totholzkonzept,” ForstBW, 2016).

### Bird surveys

We carried out point count surveys on all 135 plots during the breeding season (late February to early July), covering 100–135 plots each year from 2017 to 2022, and replicating surveys up to 3 times per year on each plot (1.72 visits on average, 6–14 visits per plot over all years). Counts took place between half an hour after sunrise and 12:00 CET, under favorable weather conditions (no rain, fog, or strong wind). From the plot center, experienced observers identified and counted all birds seen or heard within a 50‐m radius, in four 5‐min intervals preceded by a 5‐min settlement time, and ensuring that no individuals were counted repeatedly within each 5‐min period (as in Basile et al., 2021). For a given survey, the count for each species was its maximum count across the four intervals. We focused our analysis on species that occurred in more than 10 plots (all years pooled), and which nest and forage in forest habitats. We excluded raptors and corvids with large home ranges, including non‐forested areas (e.g., carrion crow, *Corvus corone*). We then classified species into nesting and foraging guilds, according to von Glutz Blotzheim and Bauer (1985).

### Invertebrate sampling

We sampled invertebrates with a combination of pitfall traps (PTs) and flight interception traps (FITs), as in Renner et al. (2024) and Vergara et al. (2021). Catches from either trap type reflect both invertebrate abundance and activity, hereafter referred to as abundance for simplicity. PTs are an established method to capture epigeal invertebrates, preyed upon by ground‐foraging bird species (e.g., Johnston & Holberton, 2009). In turn, FITs target invertebrates active above the ground layer. FITs are window traps with collectors at the top and bottom, collecting a wide range of flying insects (Knuff et al., 2019), but also able to capture non‐flying tree‐, shrub‐ and bark‐dwelling taxa, such as aphids and spiders, dropping from higher vegetation layers (Machač et al., 2018; Stenchly et al., 2007). Thus, while most often used to capture prey available to aerial‐foraging birds, FITs and similar methods have also been employed to measure prey availability for foliage‐gleaning and bark‐foraging bird species (e.g., Blüthgen et al., 2016; Iwata et al., 2003).

We placed two FITs in each plot, on its southeastern and northwestern quadrants. Traps were set at 1.5 m height, as often done in previous studies (e.g., Blüthgen et al., 2016; Müller et al., 2015), and enabling us to easily install traps on a large number of plots. However, it is important to note that the resulting catches were mostly representative of the lower vegetation layers in the forest, and may not reflect the distinctive invertebrate communities found on the canopy (Floren & Schmidl, 2008). Contents were collected every 4 weeks, from April to August 2017 (for details, see Knuff et al., 2020), and all invertebrates sorted to order or higher taxa (apart from Hemiptera, which were sorted into suborders Sternorrhyncha, Auchenorrhyncha, and Heteroptera). For our analysis, we pooled the abundance of invertebrates in each plot, but included only the first three sampling cycles (April–June), matching the bird breeding season. We selected plots with a minimum trapping effort of 30 days (*n* = 125) and focused on invertebrate taxa with >500 adult specimens captured. We excluded Thysanoptera, Acari, and Neuroptera due to their low importance in the diet of our focal bird species (von Glutz Blotzheim & Bauer, 1985). From April to May 2020, we placed three PTs per plot, at the plot center and the two FIT locations, on a representative subset of 66 of the 135 ConFoBi plots (for details, see Cordeiro Pereira, Schwegmann, et al., 2024). We collected trap catches after 21–45 days, sorted invertebrates to order level or higher, and pooled abundances for each plot. We focused on taxa with >150 adult specimens captured. We excluded Lepidoptera and Hemiptera since these groups are not epigeal, thus not being adequately captured by PTs, and are also likely less relevant as prey for ground‐foraging birds. We excluded all larvae captured by PTs or FITs, as these were collected in very low numbers compared to adults.

### Forest management, structure, composition, and abiotic environment

To describe forest maturity, as a proxy for retention measures, we used four variables: the mean tree diameter at breast height (dbh), the number of standing dead trees (snags), the mean dbh of snags, and the volume of lying deadwood (lying DW). We derived the first three from an inventory carried out in all plots in 2016–2018, including all trees with dbh > 7 cm. The volume of lying deadwood was estimated through a V‐transect carried out alongside the forest inventory, including only coarse woody debris (for details, see Storch et al., 2020). No harvesting took place in the plots since their establishment in 2016.

To represent close‐to‐nature forestry practices, we used four variables: the SD of tree dbh, the share of plot basal area belonging to broadleaved trees (broadleaf share), the tree species richness in the plot, and the canopy cover. We calculated the first three variables from the aforementioned tree inventory. Canopy cover was calculated from a canopy height model of each plot, created with UAV‐SfM (Unoccupied Aerial Vehicle–Structure from Motion) flights (Frey et al., 2018), defining it as the proportion of plot area with vegetation >5 m. Flights were conducted twice in each plot, in 2017–2018 and again in 2019–2020.

In addition, we measured four variables representing other facets of forest structure and composition that are not directly modified by management: the effective number of layers (ENL), shrub‐layer cover, herb‐layer cover, and understory vascular plant richness (understory SR). The ENL (Ehbrecht et al., 2016) was calculated from terrestrial laser scanning (TLS) with Faro Focus 3D laser scanners (Faro Technologies Inc., Lake Mary, Florida) during the growing seasons of 2017 and 2018, in the three invertebrate trapping locations in each plot (for details, see Frey et al., 2019; Knuff et al., 2020). We averaged ENL values from these three locations to obtain a plot‐level value. High ENL values reflect multilayered stands with evenly filled layers (Ehbrecht et al., 2016). Herb‐ and shrub‐layer covers were the average cover of vegetation <1 m height (including woody species) and between 1 and 5 m height, respectively, over a grid of six 5 × 5 m subplots (see Helbach et al., 2022), measured in the growing seasons of 2016–2018. In 2020, we conducted a second vegetation survey on 61 of the 66 plots where PTs were also placed, measuring cover in 18 circular 1‐m^2^ subplots. We calculated understory SR aggregated from all vegetation subplots in each plot, in 2016–2018 and 2020. Understory species richness included all plants up to 5 m tall in 2016–2018 and only up to 1.5 m in 2020, but these values were strongly correlated (ρ = 0.84).

To account for the abiotic environment, which may strongly affect the activity and abundance of invertebrates, we calculated the average elevation of the plot, the northness index, and the SD of the slope, from a Digital Terrain Model of our study area with 1‐m resolution (LGL, 2018). We used the SD slope within each plot to describe small‐scale heterogeneity in soil and climate (as in Heidrich et al., 2020). We used the northness index, which ranges from −1 for southern aspects to 1 for northern aspects, to represent the solar exposure of plots. Table 1 summarizes the values for all variables across our plots.

**TABLE 1:** Descriptive statistics and years of measurement for all predictors used to model the abundance of bird species and invertebrate groups, across all plots (*n* = 135) and for the subset of plots with pitfall traps (*n* = 66).

Predictor	Year	Units	Range		Mean (±SD)	
			All plots	Subset	All plots	Subset
Mean dbh	2016–2018	cm	12.2–52.6	12.2–52.6	30.2 ± 8.3	30.1 ± 8.2
No. snags	2016–2018	…	0–394	0–394	33.5 ± 53.9	48.1 ± 73.2
Snag dbh	2016–2018	cm	0–59.3	0–52.2	24.9 ± 10.4	26.5 ± 11.1
Lying deadwood volume	2016–2018	m^3^	2.7–282.9	6.7–282.9	43.6 ± 43.9	57.2 ± 55.6
ENL	2017–2018	…	7.15–32.96	7.15–32.96	18.95 ± 4.96	19.62 ± 4.82
SD dbh	2016–2018	cm	7.7–24.6	8.2–24.6	14.8 ± 3.6	15.2 ± 3.7
Canopy cover	2017–2018	%	12.3–95.2		71.9 ± 14.8	
	2019–2020	%	12.4–98.2	17.9–98.2	72.8 ± 15.7	70.5 ± 15.8
Shrub cover	2016–2018	%	0–55		12.8 ± 12.5	
	2020	%		0–50.6		13.2 ± 13.2
Herb cover	2016–2018	%	0.1–73.8		35.0 ± 18.8	
	2020	%		0–87.3		37.8 ± 24.9
Broadleaf share	2016–2018	%	0–96.2	0.3–92.4	28.8 ± 26.0	34.2 ± 25.4
Tree SR	2016–2018	…	2–15	2–10	5.5 ± 2.1	5.1 ± 1.6
Understory SR	2016–2018	…	2–58		24.6 ± 12.2	
	2020	…		0–46		20.1 ± 11.2
Elevation	…	m	443–1334	512–1334	822 ± 183	861 ± 194
SD slope	…	%	0.7–12.7	1.1–11.5	4.0 ± 2.3	4.6 ± 2.2
Northness	…	…	−0.98 to 0.99	−0.96 to 0.97	0.06 ± 0.62	0.14 ± 0.60

Abbreviations: ENL, effective number of Layers; SR, species richness.

### Statistical analyses

#### Bird abundance (questions 1 and 2)

We modeled the abundance of each bird species with static single‐species *N*‐mixture models, accounting for imperfect detection (Royle, 2004). These are hierarchical models composed of an ecological sub‐model for species abundance *N*_*i*_ at plot *i* (Poisson‐distributed), and a detection sub‐model, conditional on *N*_*i*_, that explains the observed counts *C*_*ij*_ in each replicate survey *j* of plot *i* (binomial‐distributed). In the ecological sub‐model, we included the plot‐level variables described in the previous section (hereafter forest predictors). We verified that no strong collinearities (|ρ| > 0.7) were present among forest predictors. To prevent overfitting, we carried out an a priori selection of predictors relevant to each species, according to their foraging and nesting requirements in von Glutz Blotzheim and Bauer (1985). We log‐transformed the number of snags and the lying deadwood volume, and applied a square root transformation to shrub cover, to reduce skew in these variables' values. The detection sub‐model for each species included fixed effects of the ordinal date and minutes since sunrise, as well as a random effect of observer identity (10 levels), all of which may influence bird detectability. We scaled all predictors (mean = 0, SD = 1) to facilitate convergence.

To account for multiple years of bird count data, we adopted a “stacked” approach in our models (as in Rhinehart et al., 2024), in which we treated abundance for each plot‐year combination as a separate data point. Then, we added random effects of plot identity and year to the abundance sub‐model, thereby allowing abundances to vary across years and accounting for non‐independence among data points. This also enabled us to specify predictor values that vary across years, that is, using replicated canopy cover values for the period 2019–2020 to model bird abundances from 2019 onwards, and replicated vegetation survey data from 2020 (where available) to model abundances from 2020 onwards.

We ran the *N*‐mixture models in a Bayesian framework with R package *ubms* (Kellner et al., 2022), using weakly informative priors for our parameters (Appendix S1: Section S1). For each model parameter, we approximated its posterior distribution with 8 Markov chain Monte Carlo (MCMC) chains with 2000 iterations, from which the first 1000 were discarded. We considered that a model had converged if the Gelman–Rubin statistics ($\hat{R}$) of all effect parameters were <1.1 (Gelman & Hill, 2006) and there were no divergent transitions in the algorithm. If we did not achieve convergence, we refitted the model with stepwise removal of the random effects of plot identity, year and observer (in this order) and chose the best‐fitting model, that is, with highest expected log predictive density (ELPD), calculated through leave‐one‐out cross‐validation (Vehtari et al., 2017). We then checked goodness of fit of each species' model with posterior predictive checks, and calculated a model‐level Bayesian *p* value (Conn et al., 2018; Kéry & Royle, 2016). If below 0.05, indicating overdispersed residuals, we refitted that model with R package *spAbundance* (Doser et al., 2024), where we could account for overdispersion by specifying a negative binomial distribution for the abundance sub‐model. On *spAbundance*, we built 3 MCMC chains with 250,000 samples, discarding the first 100,000, and keeping every 150th sample. These models required more iterations and longer running times than *ubms* models, due to the different MCMC algorithms used and higher number of parameters. We assessed convergence and goodness of fit as above. In all our final models, we considered the effect of a predictor to be significant if 90% of the posterior distribution of its coefficient did not overlap 0 (as in Basile et al., 2021). All priors, MCMC specifications and R code for the models are shown in Appendix S1.

#### Invertebrate groups (question 3)

We modeled the abundance of each invertebrate group as a function of forest predictors, using Bayesian generalized linear models (GLMs) with R package *brms* (Bürkner, 2017). We built 4 chains with 2000 iterations, from which the first 1000 were discarded. We specified the same weakly informative priors as for the bird abundance models and used the same criteria for assessing convergence and goodness of fit, besides calculating a Bayes *R* ^2^ for each model (Gelman et al., 2019). We used a negative binomial distribution with log‐link for invertebrate abundances to account for overdispersed residuals. If overdispersion remained, we refitted models with a Poisson distribution, added an observation‐level random effect (Harrison, 2014), and rechecked goodness of fit. All models included sampling effort as offsets (number of traps multiplied by number of days for PTs and number of days for FITs). We scaled all predictors by the mean and SD of their values over all plots. We chose a relevant set of predictors a priori for each invertebrate group based on the available literature. Since the sample size was smaller for PTs (*n* = 66) than for FITs (*n* = 125), we carried out further variable selection for each PT model. We achieved this in three steps—we first fitted the full model in a frequentist framework with R package *lme4* (Bates et al., 2015); then used function *dredge()* from R package *MuMIn* (Bartoń, 2023) to rank all possible nested models containing only 6 predictors by the Akaike information criterion corrected for small sample sizes (AIC_c_); lastly, we selected the top‐ranked model and fitted it with *brms*. In all our final models, we considered the effect of a predictor on invertebrate abundance to be significant if 90% of its posterior distribution did not overlap 0.

#### Direct and trophic‐mediated effects (question 4)

To address our last research question, we employed piecewise structural equation models (SEM) (Lefcheck, 2016), a form of confirmatory path analysis that allowed us to test hypothesized causal relationships between forest predictors and bird abundances, discerning direct effects from those mediated by abundance of invertebrate groups (as in Vergara et al., 2021). We used R package *piecewiseSEM* (Lefcheck, 2016), based on a local estimation approach (Shipley, 2000), that accommodates non‐Gaussian error structures. We built two global SEMs—one with epigeal invertebrate groups and ground‐foraging birds (using data from 2020 in 57 plots) and another with FIT invertebrate groups and foliage‐gleaning and bark‐foraging birds (using data from 2017 in 97 plots). In these SEMs, the total abundance of birds in each guild was modeled as a function of forest predictors, as well as of the total abundance of potential prey (all PT groups or all FIT groups), adjusted for sampling effort. We excluded Hymenoptera from PT totals since this group was mostly composed of red wood ants (*Formica* spp.), which are relevant in the diet of only a few bird species (von Glutz Blotzheim & Bauer, 1985). In turn, the total abundance of invertebrate prey was modeled as a function of forest predictors. We also hypothesized multiple links among forest predictors, such as an influence of canopy cover on shrub and herb cover. In addition to the global SEMs, we ran a more specific SEM with the total abundance of small‐sized ground‐foragers (European robin, *Erithacus rubecula*; Eurasian wren, *Troglodytes troglodytes*; and dunnock, *Prunella modularis*) and the abundance of epigeal spiders (but not epigeal beetles, due to the large size of most specimens). We describe all hypothesized causal links, error distributions, and link functions in Appendix S2. To simplify the SEMs and prevent overfitting, we used only predictors that had been identified as significant correlates of bird and invertebrate abundances in the previous steps of analysis. As bird abundances, we used estimates from the *N*‐mixture models, but refitted these to include only an intercept and random effects in their abundance component, in order to prevent inflated Type 1 errors (as in Duclos et al., 2019; Higgins et al., 2021). We extracted the median of posterior draws of abundance of each species in each plot and year, and summed these values across species in each foraging guild (excluding species with unrealistic abundance estimates, that is, >20 individuals per plot, likely due to flocking behavior).

We assessed the global fit of each SEM with Fisher's *C* test of directed separation (Shipley, 2013). If *p* < 0.05, the hypothesized causal structure was not supported by the data, and we inspected individual d‐separation claims to identify missing correlations. We then added these to the SEM as free correlations (assuming no causal link) and rechecked global fit. Following this, we compared the fit of SEMs with and without links between invertebrate and bird guild abundances, using an AIC_c_ derived from Fisher's *C* (Shipley, 2013) to assess the importance of these paths. Lastly, we plotted the supported causal structures with R package *DiagrammeR* (Iannone, 2022).

## RESULTS

### Effects of forest predictors on bird abundances (questions 1 and 2)

**FIGURE 2:**
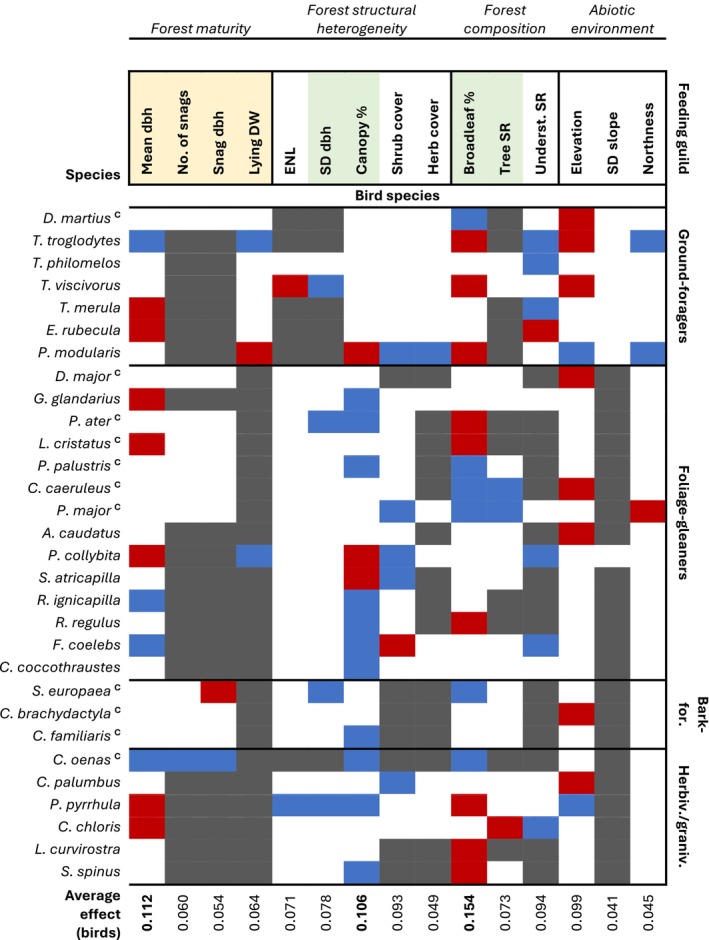
Summary of effects of plot‐level predictors on the abundance of bird species. Cell colors indicate whether a predictor was excluded a priori (dark gray), non‐significant (90% CI overlapping 0, white), significant with a positive effect (90% CI above 0, blue), or with a negative effect (90% CI below 0, red). Bird species (on the left, with genus name abbreviated to its first letter) are further grouped by foraging guild (on the right). Cavity‐nesting birds are denoted with a “c” in superscript after the species name. Forest predictors are grouped into four categories, and shaded according to whether they represent retention forestry (shaded yellow) or close‐to‐nature (shaded green) forestry practices. Weighted averages of the effects of each predictor on bird abundances are shown at the bottom (averages weighted by the inverse of the length of the 90% CI for each effect). Means and 90% CI for each effect are shown in Appendix S5. bark‐for., bark‐foragers; DW, deadwood; herbiv./graniv., herbivores/granivores; No., number; SR, species richness; underst., understory.

From 2017 to 2022, we carried out 1394 bird surveys, detecting 81 bird species (Appendix S3). Among these, we selected 32 species for our analysis (Figure 2), of which two (fieldfare, *Turdus pilaris*; and spotted nutcracker, *Nucifraga caryocatactes*) were further excluded due to non‐converging *N*‐mixture models. Our focal set of species included 14 foliage‐gleaning invertivorous species, seven ground‐foraging invertivorous species, three bark‐foraging invertivorous species, and six herbivorous/granivorous species. Eleven species were cavity‐nesters. *N*‐mixture models yielded mean abundances ranging from 0.12 (stock dove, *Columba oenas*) to 6.46 (coal tit, *Periparus ater*) individuals per plot and indicated that we detected on average between 8.2% (short‐toed treecreeper, *Certhia brachydactyla*) and 51.1% (Eurasian chaffinch, *Fringilla coelebs*) of individuals present (summarized model outputs and diagnostics in Appendix S4).

Forest maturity variables, reflecting retention forestry practices, significantly affected the abundances of species across all four foraging guilds, and effects also extended across nesting guilds (Figure 2, all effect plots in Appendix S5). For instance, the volume of lying deadwood was positively associated with the abundance of two ground‐nesting species, the Eurasian wren and the common chiffchaff (*Phylloscopus collybita*). Mean tree dbh showed positive effects on the abundance of four species and negative effects on the abundance of seven others. Among cavity‐nesting birds, the number of snags and their dbh showed effects only on stock dove and Eurasian nuthatch (*Sitta europaea*). In fact, the abundance of stock doves significantly increased with all three forest maturity variables included in this species' model.

**FIGURE 3:**
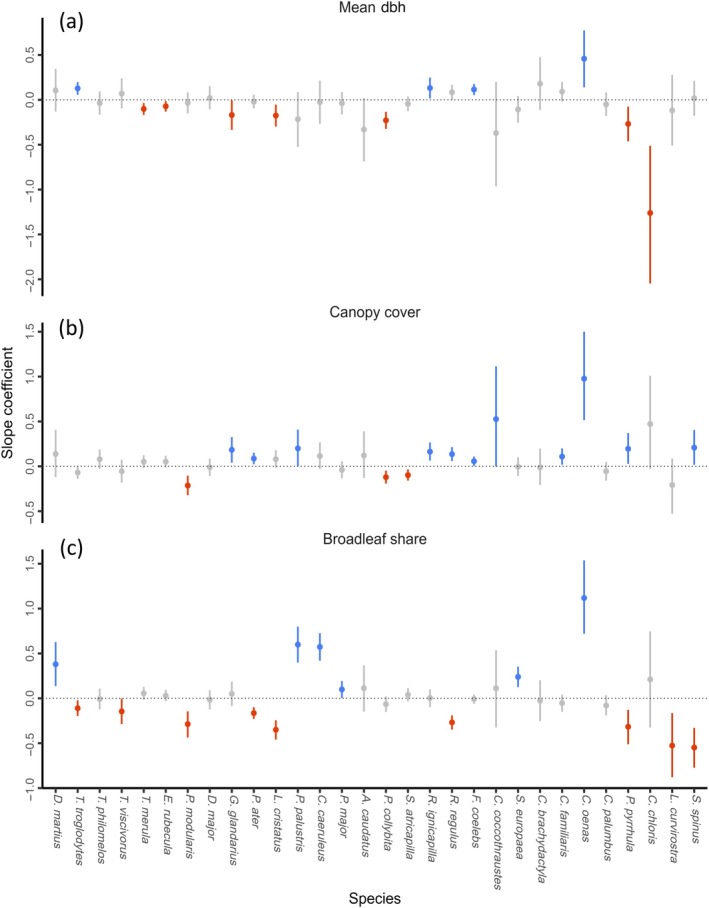
Posterior means and 90% CI of the effects of three management‐related predictors on the abundances of the 30 focal bird species (horizontal axis, with abbreviated genus name): (a) mean dbh, representing retention practices, (b) canopy cover, and (c) broadleaf share, both representing close‐to‐nature forestry.

Variables reflecting close‐to‐nature forestry practices affected the abundance of more species than forest maturity variables (Figure 2). In particular, canopy cover and broadleaf share had the strongest effects across most species, and the effects of broadleaf share were on average stronger than those of mean tree dbh (Figure 3). Six species responded positively to an increasing share of broadleaf trees, while nine responded negatively. The abundance of 11 species was higher in plots with higher canopy cover, while the abundance of three others was higher in open‐canopy stands. Only three species responded to higher tree species richness (two of them positively), and four species to the standard deviation of tree dbh (all positively). Moreover, the abundances of multiple species increased with shrub cover and with understory richness, and responded significantly to the abiotic environment, especially to elevation (significant effects on 10 species, of which eight were negative).

### Effects of forest predictors on invertebrate groups (question 3)

**FIGURE 4:**
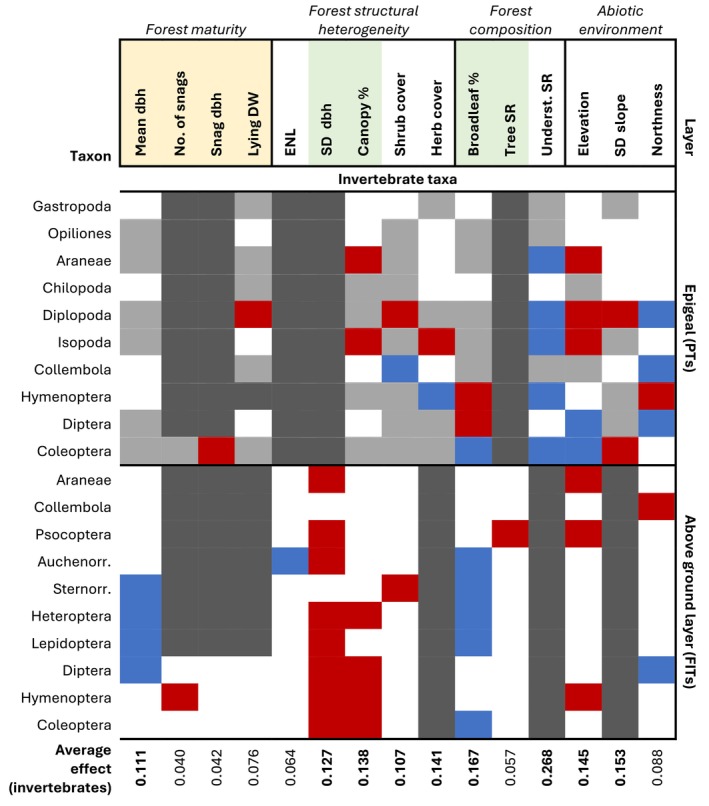
Summary of effects of plot‐level predictors on the abundance of invertebrate groups. Cell colors indicate whether a predictor was excluded a priori (dark gray), excluded following automatic variable selection (light gray), non‐significant (90% CI overlapping 0, white), significant with a positive effect (90% CI above 0, blue), or with a negative effect (90% CI below 0, red). Invertebrate groups (on the left) are further grouped by vertical layer and corresponding trapping method (on the right). Forest predictors are grouped into four categories, and shaded according to whether they represent retention forestry (shaded yellow) or close‐to‐nature (shaded green) forestry practices. Weighted averages of the effects of each predictor on invertebrate abundances are shown at the bottom (averages weighted by the inverse of the length of the 90% CI for each effect). Means and 90% CI for each effect are shown in Appendix S5. Auchenorr, Auchenorrhyncha; DW, deadwood; FITs, flight interception traps; No., number; PTs: pitfall traps; SR, species richness; Sternorr., Sternorrhyncha; underst., understory.

**FIGURE 5:**
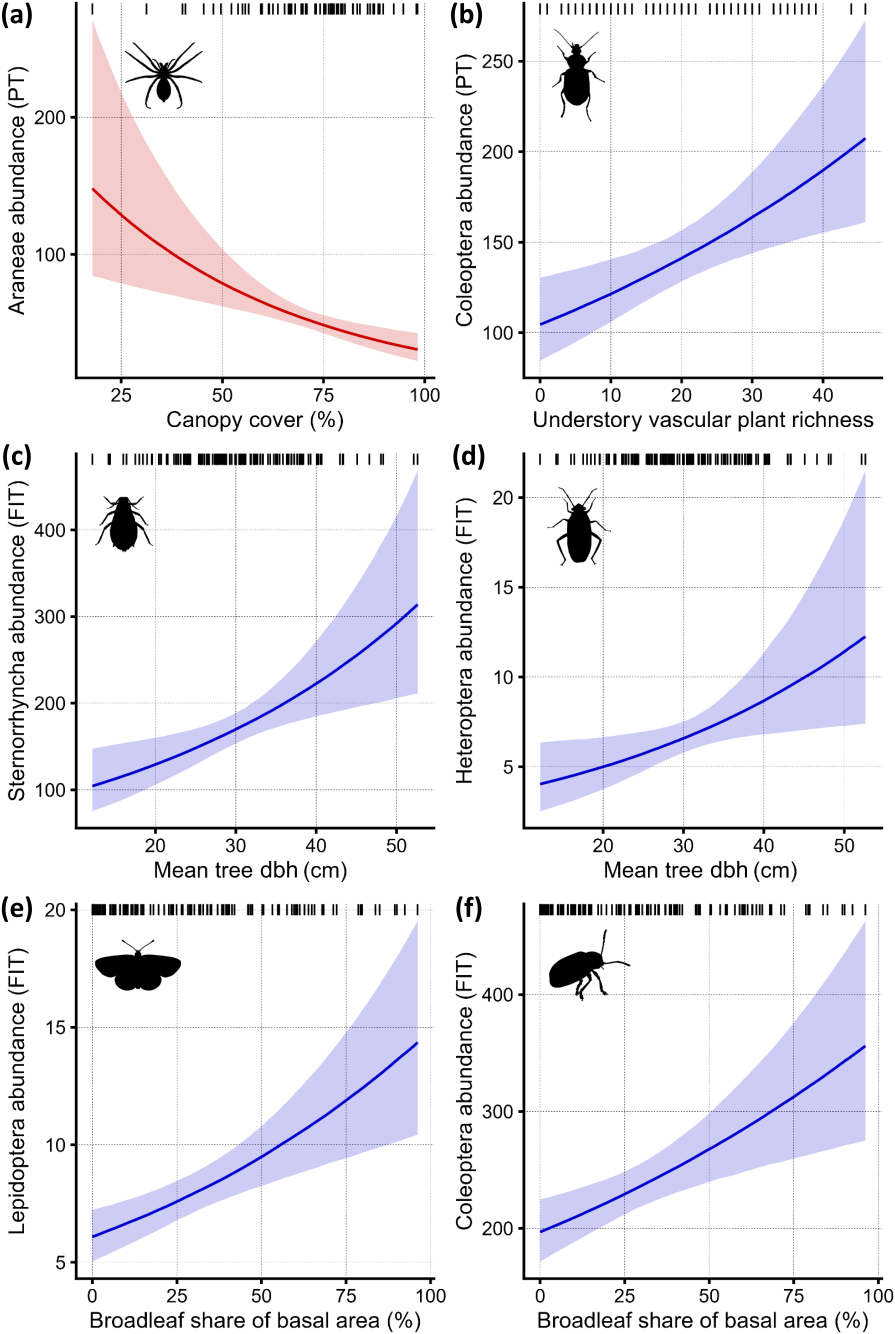
Six examples of significant marginal effects of forest predictors (horizontal axes) on abundances of invertebrate groups (vertical axes; standardized for sampling effort, and represented by icons). On vertical axes, PT and FIT denote whether an invertebrate group was captured on pitfall traps or flight interception traps, respectively. The posterior means (solid line) and the 90% credible interval (shaded area) are shown, and the color reflects the direction of the effect (red: Negative; blue: Positive). All icons are licensed under CC0 1.0 (Universal Public Domain Dedication), sourced from: phylopic.org/images/16b94f15‐3072‐43ec‐a6b0‐39f200b16399 (by T. Michael Keesey, panel a); phylopic.org/images/9c1c33c0‐db2f‐472f‐a94b‐56b1553e4a45 (by Prespa Research Group, panel b); phylopic.org/images/cd57935f‐334e‐4067‐8078‐3c0dc1f44bf0 (by Christoph Schomburg, panel c); phylopic.org/images/abb918ae‐6d3d‐4dd3‐a6eb‐e5457199f25f (by Dave Angelini, panel d); phylopic.org/images/a669c1df‐139a‐419d‐93ac‐44fc0da50fef (by Andy Wilson, panel e); phylopic.org/images/7c72a017‐e297‐4867‐ac96‐8a2e05ad8ba7 (by Andy Wilson, panel f).

We included in our analysis 40,797 specimens from PTs (618.1 per plot on average), belonging to 10 groups, and 114,279 specimens from FITs (914.2 per plot on average), belonging to 10 groups (Figure 4). The two most numerous groups captured in PTs were Hymenoptera and Coleoptera, and in FITs, Diptera and Coleoptera (see Appendix S6 for a summary of invertebrate models). The forest predictor with the strongest average effect on the abundances of invertebrate groups was the understory plant richness, followed by the broadleaf share (Figure 4). Among the 10 epigeal invertebrate groups analyzed, five increased in abundance with understory plant richness (e.g., epigeal Coleoptera, Figure 5b), and none was negatively affected.

We found effects of variables reflecting retention and close‐to‐nature forestry on both epigeal invertebrates and those active above the ground layer, but it was the latter management type that had more pronounced effects across more groups (Figure 4). Forest maturity variables did not show positive effects on any epigeal group, but four groups captured by FITs (Sternorrhyncha, Heteroptera, Lepidoptera and Diptera) showed significantly higher abundances in plots with higher mean tree dbh (e.g., Figure 5c,d). On the other hand, all significant responses of invertebrate groups to standard deviation of tree dbh (8 of 10) and canopy cover (6 of 16) were negative, and all significant responses of FIT invertebrate groups to broadleaf share (5 of 10) were positive. For instance, abundances of epigeal spiders were lower in plots with higher canopy cover (Figure 5a), and plots with a higher broadleaf share showed higher abundances of Lepidoptera (Figure 5e) and Coleoptera (Figure 5f).

### Links between invertebrate abundance and bird abundance (question 4)

Responses of invertivorous bird species to forest predictors did not clearly align with those of invertebrate groups to the same predictors. For example, half of foliage‐gleaning bird species responded positively to canopy cover, in contrast with the negative effect of canopy cover on multiple FIT invertebrate groups (Figures 2 and 4). On the other hand, some bird species and invertebrate groups showed responses in a similar direction to predictors not directly related to management, for example, negatively to elevation and positively to understory plant richness (Figures 2 and 4).

**FIGURE 6:**
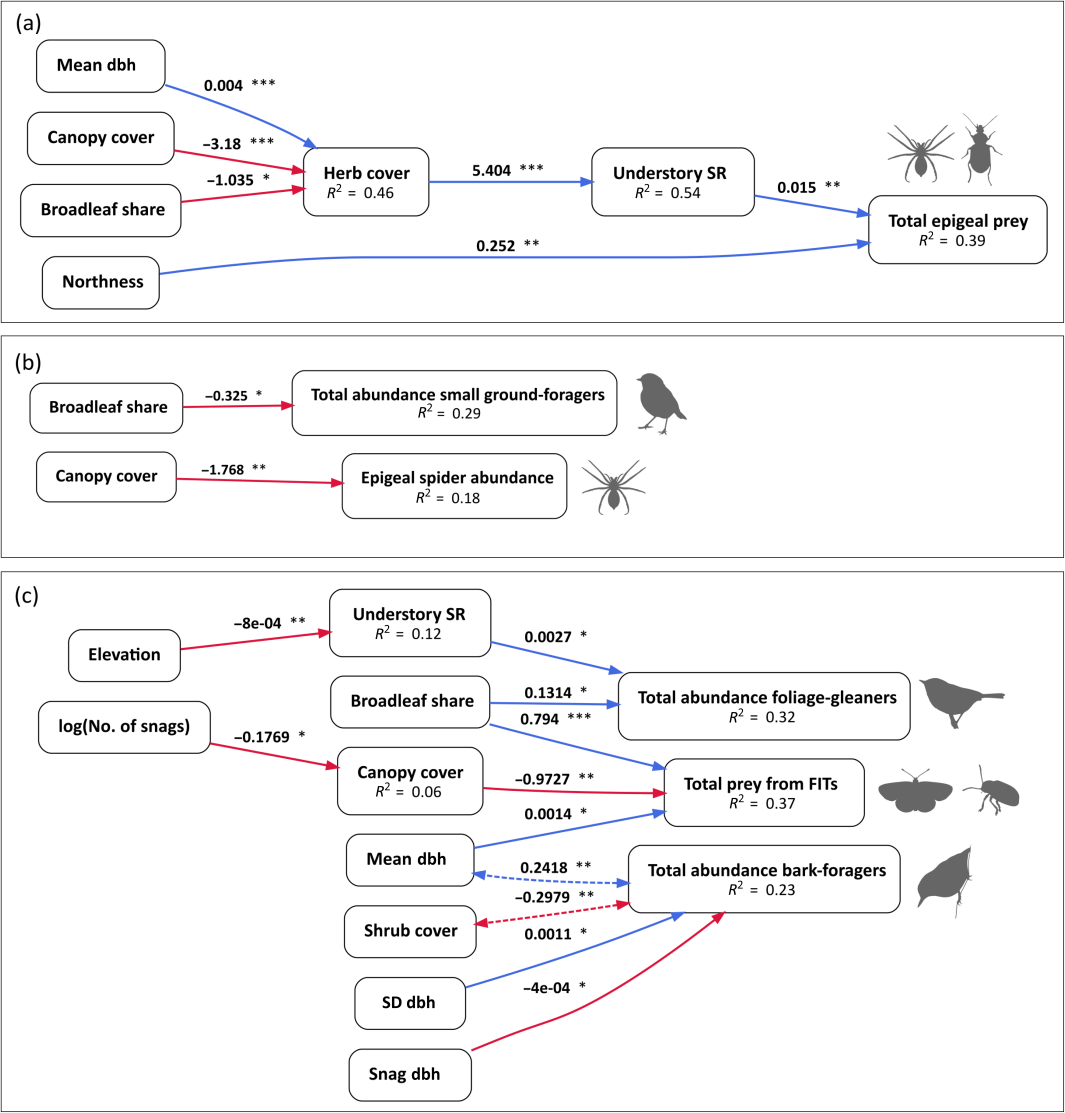
Simplified path diagrams for piecewise structural equation models (SEMs), showing significant links of bird and invertebrate abundances with their causal ancestors (diagrams with all significant links are shown in Appendix S7): (a) links of forest predictors with total abundance of epigeal prey (*n* = 57, data from 2020; total abundance of ground‐foraging birds is not shown, as it had no significant causal ancestors), (b) links of forest predictors with total abundance of small ground‐foraging birds (summed abundances of *Erithacus rubecula*, *Troglodytes troglodytes* and *Prunella modularis*) and abundance of epigeal spiders (*n* = 57, data from 2020), and (c) links of forest predictors with total abundances of foliage‐gleaning birds, bark‐foraging birds, and invertebrates from flight interception traps (*n* = 97, data from 2017). Path colors denote whether the relationship is negative (red) or positive (blue). Variance explained by causal ancestors (*R* ^2^) is shown below the variable name. Next to each path, the unstandardized coefficient is shown, alongside its significance level (*p < 0.05; **p < 0.01; ***p < 0.001). Dashed bidirectional arrows denote free correlations, which were not included in the initial SEMs, but were suggested by directed separation tests. All icons are licensed under CC0 1.0 (Universal Public Domain Dedication), sourced from: phylopic.org/images/b0d54194‐4105‐4694‐b670‐b693df1640cf (by Anthony Caravaggi, robin on panel b); phylopic.org/images/5d1edc19‐b18f‐4d76‐8dae‐84b095c3f4c2 (by Jiekun He, warbler on panel c); phylopic.org/images/4f24cf78‐48d8‐42ab‐9e12‐18853528c73c (by Andy Wilson, nuthatch on panel c); see caption of Figure 5 for invertebrate icons. FITs, flight interception traps; No., number; SR, species richness.

At the foraging guild level, our data did not support the hypothesized causal structures in global SEMs for the ground‐foraging guild (Fisher's *C* statistic = 171.302, *p* = 0) and for the foliage‐gleaning and bark‐foraging guilds (Fisher's *C* statistic = 276.907, *p* = 0). After including in our SEMs multiple free correlations suggested by d‐separation tests (Appendix S2), we achieved models with a good fit (full diagrams in Appendix S7; ground‐foraging guild: Fisher's *C* = 74.932, *p* = 0.448; foliage‐gleaning and bark‐foraging guilds: Fisher's *C* = 70.570, *p* = 0.852). In both SEMs, paths linking total prey abundance with guild‐level bird abundance were non‐significant (*p* > 0.05) and removing them resulted in a much lower AIC_c_ (ground‐foraging guild: ΔAIC_c_ = 59.67; foliage‐gleaning and bark‐foraging guilds: ΔAIC_c_ = 137.34), indicating that effects of forest predictors on guild‐level bird abundance are not mediated by the abundance of the assessed invertebrate groups in the lower forest layers (Figure 6a,c). Nevertheless, total abundance of foliage‐gleaners and total abundance of FIT invertebrates both increased with broadleaf share (Figure 6c). In some cases, links between forest predictors and bird or invertebrate abundances were mediated by other forest predictors. For instance, canopy cover did not directly affect total abundance of epigeal invertebrates, but it did so indirectly, due to lowered herb layer cover in closed‐canopy forests, reducing understory vascular plant richness, which in turn affected epigeal invertebrates (Figure 6a).

As for the SEM linking forest predictors, small‐sized ground‐foraging birds and epigeal spiders, our data fitted the hypothesized causal structure (Fisher's *C* statistic = 37.428, *p* = 0.496), but as in the global SEMs, there was no evidence for a causal link between spider and bird abundance (*p* > 0.05, ΔAIC_c_ = 8.726). Small ground‐foragers responded negatively to increased broadleaf share, whereas spider abundance responded negatively to canopy cover (Figure 6b).

## DISCUSSION

We found that both bird species and invertebrate groups clearly responded to multifunctional forestry as implemented in our study area. Among birds, species' abundances responded more strongly to predictors representing close‐to‐nature forestry than to those representing retention practices, but the direction of effects varied across species. Invertebrate abundances also responded to both forms of management, but responses did not clearly align with those of birds, and piecewise SEMs did not support direct links between the abundance of birds and that of the invertebrates we sampled in the lower forest layers. Nonetheless, there was indication that some forest predictors act as shared drivers of bird and invertebrate abundances. Below, we discuss in detail the observed effects of retention and close‐to‐nature forestry practices on birds, invertebrates, and links between the two.

### Effects of forest predictors on bird species (questions 1 and 2)

As expected, the effects of forest maturity variables on bird abundance spanned across all studied foraging and nesting guilds, and the direction of effects varied also across species. The responses of individual species to mean tree dbh reflect their preferences for specific stages of forest development (Begehold et al., 2015). However, we did not find a clear preference of most species for higher tree dbh (Begehold et al., 2015; Komlós et al., 2024), which may be attributed to the absence of the oldest successional stages in our region (Sabatini et al., 2018). Still, we found a positive link between the volume of lying deadwood and the abundances of two ground‐nesting species. These structures may provide more sheltered nesting sites to ground‐nesters, and the Eurasian wren is known to nest frequently under fallen logs (Piechnik et al., 2020). In clearcutting systems, higher retention levels are associated with lower abundances of ground‐nesters (Basile et al., 2019), but our findings suggest that ground‐nesters may benefit from retaining downed deadwood within forests managed under close‐to‐nature forestry.

As hypothesized, predictors representing retention forestry practices had an overall weaker effect than those linked to close‐to‐nature forestry. Forest birds may exhibit a response to forest maturity only above certain thresholds of stand age or deadwood availability (Birčák & Reif, 2015; Moning & Müller, 2008; Müller & Bütler, 2010), and retention practices may be yet too recent to achieve their full potential (Rosenvald et al., 2019). In fact, aerial‐foraging birds were rare in our study area, even though this guild is characteristic of old‐growth montane forests (Adamík et al., 2003). Besides, few cavity‐nesters showed significant responses to forest maturity variables. In managed forests, woodpeckers are the chief providers of nesting sites for secondary cavity‐nesters (Wesołowski & Martin, 2018). Neither of the two woodpecker species analyzed responded to retention variables, potentially explaining a lack of response of most secondary cavity‐nesters. The great spotted woodpecker (*Dendrocopos major*) is a habitat generalist, abundant across most forest development stages (Kosiński, 2006; Pasinelli, 2007). Moreover, neither woodpecker species fully relies on snags for foraging in the breeding season (von Glutz Blotzheim & Bauer, 1985; Rolstad et al., 1998). This contrasts with more specialized woodpecker species (e.g., three‐toed woodpecker, *Picoides tridactylus*), which we rarely recorded. Contrary to expected, two species of secondary cavity‐nesters even showed a negative response to the mean dbh of living trees and snags. Smaller secondary cavity‐nesters may select for nesting sites in smaller trees (as in Robles et al., 2011) if these cavities show higher suitability (e.g., smaller entrance sizes, lower rates of internal decay, or reduced search efficiency by predators), independently of the number of cavities available or their origin (Camprodon et al., 2008; Edworthy & Martin, 2014).

As for the effects of close‐to‐nature forestry, the broadleaf share was the most important predictor of bird abundances, but this effect also varied in direction across species. This reflects the close associations of European forest birds with either broadleaved or conifer forests, rather than particular tree species (Felton et al., 2010; Kameniar et al., 2023; Szarvas et al., 2024). It is also notable that several cavity‐nesters (e.g., black woodpecker, *Dryocopus martius*; and marsh tit, *Poecile palustris*) responded to increased broadleaf share rather than to forest maturity. The microhabitats that commonly develop on European beech (e.g., rot holes) differ from those formed in conifer species (Spînu et al., 2022), and previous research has shown that focusing retention efforts on broadleaved and pioneer tree species accelerates the development of old‐growth features (Rosenvald et al., 2019; Spînu et al., 2023). Besides, woodpeckers avoid excavating nests on living spruce and fir (Hebda et al., 2017; Spînu et al., 2022). In contrast, old beech trees with heart rot are key structures for the black woodpecker (Puverel et al., 2019; Zahner et al., 2012) and for the stock dove that uses its cavities (Kosiński et al., 2010). Thus, our results support the notion that the ongoing conversion to mixed stands including European beech not only fosters the coexistence of multiple bird species but is also especially beneficial for cavity‐nesters.

As for canopy cover, this was a more important predictor of bird species' abundances than an uneven age structure, in line with previous studies (Kameniar et al., 2021; Moning & Müller, 2008). Our results reveal a bird assemblage characteristic of closed‐canopy forests, which can thus benefit from close‐to‐nature forestry, maintaining continuous canopy cover. In contrast, bird species dependent on large canopy gaps, which would benefit from bark beetle disturbances or clearcuts (Moning & Müller, 2008; Paquet et al., 2006; Przepióra et al., 2020), had low frequencies on our plots and were underrepresented among our focal species. Importantly, this includes species declining at the regional level, such as willow warbler (*Phylloscopus trochilus*) and common redstart (*Phoenicurus phoenicurus*) (Kramer et al., 2022). Forest gaps may also act as an important refuge for declining farmland birds (Graser et al., 2025; Ram et al., 2020; Żmihorski et al., 2016). Besides, studies in primeval forests in Poland have demonstrated that even small canopy gaps result in characteristic bird assemblages that differ from closed‐canopy sites (Fuller, 2000; Lewandowski et al., 2021).

### Effects of forest predictors on invertebrate prey groups (question 3)

Both retention and close‐to‐nature forestry practices significantly affected the abundance of potential prey for invertivorous birds in the lower forest layers, although retention had only limited effects on epigeal invertebrates. As described for carabid beetles in the same plots (Cordeiro Pereira, Schwegmann, et al., 2024), mean tree dbh and downed deadwood may be more important drivers of invertebrate species richness than abundance, thanks to increased forest floor heterogeneity. Nonetheless, the mean tree dbh showed a positive effect on four herbivorous insect groups, which can be attributed to the larger green biomass available for consumption in mature forests (Leidinger et al., 2019).

Among predictors representing close‐to‐nature forestry, the broadleaf share had the strongest effect on invertebrate abundances. Beech litter hosts a higher biomass of predatory insects than litter originating from spruce (Scheu et al., 2003). Beech leaves are also more palatable and nutrient‐rich for herbivores than conifer needles (Ampoorter et al., 2020). This can explain the positive effects of broadleaf share on the abundances of Coleoptera (from both trap types), Lepidoptera, and all sub‐orders of Hemiptera. Whereas a higher broadleaf share was linked to a higher abundance of several invertebrate groups, an uneven age structure and closed canopy were linked with lower abundances of invertebrates from FITs, epigeal spiders, and isopods. This is consistent with previous research (Černecká et al., 2020; Penone et al., 2019) and can be explained by decreased light penetration to lower vegetation layers and a colder microclimate (Bouget & Duelli, 2004; Brüllhardt et al., 2020). Knuff et al. (2020), using the same FITs as in our study, found a positive effect of multilayered vegetation on invertebrate abundances, but we focused only on the spring months, in which microclimactic conditions may be more limiting for invertebrate abundances than in summer.

The richness of understory plants was the strongest predictor of epigeal invertebrate abundances, with an overall positive effect. Species‐rich plant communities may enable higher productivity and higher C:N ratios (Ebeling et al., 2014) and provide a diverse set of resources, potentially more stable over time (Haddad et al., 2011; Sperandii et al., 2025). Thus, a species‐rich understory can sustain higher abundances of phytophagous, saprophagous, and omnivorous invertebrates (Ebeling et al., 2014), in turn attracting higher abundances of predatory invertebrates (Haddad et al., 2009; Lange et al., 2014). Nonetheless, further research is needed on the bottom‐up effects of understory diversity on forest food webs, as most studies on the topic have focused on grasslands.

### Trophic‐mediated effects of forest predictors on bird abundances (question 4)

Contrary to our expectation, responses of bird and invertebrate abundances to the same management‐related predictors were not congruent. This suggests that effects of multifunctional forestry on abundances of invertivorous birds are not trophic‐mediated, at least regarding the invertebrate groups and forest layers we surveyed. This was reinforced by our SEMs, which did not support a numerical response of bird guild abundances to prey availability. For instance, we found that canopy gaps increase the availability of invertebrate food resources, as proposed by previous studies in montane forests (e.g., Lewandowski et al., 2021; Przepióra et al., 2020). However, in contrast to those studies, the response of most foliage‐gleaning invertivores to canopy cover was positive. Moreover, while canopy openness directly increased the abundance of epigeal spiders and indirectly increased the overall abundance of epigeal invertebrates, this did not translate into higher abundances of ground‐foraging birds. Invertivorous forest birds in Europe exhibit a relatively low level of diet specialization (Morelli et al., 2021). That considered, the invertebrate supply in mature temperate forests during the breeding season may easily exceed their metabolic needs (Rosenvald et al., 2011; Wesołowski, 2007). Accordingly, numerical responses of temperate forest birds to invertebrate abundances are probably less common than functional responses to spatial and temporal patterns in prey availability, for example, by switching prey types and foraging substrates (Getman‐Pickering et al., 2023; Hogstad, 2004), or matching phenology to peaks in high‐quality food supplies (Jones et al., 2003; Perrins, 1991). This contrasts with boreal forests, where bottom‐up effects of insect abundance on bird abundance are likely more prevalent, due to simpler food webs and prey availability being more constrained in time (Folkard & Smith, 1995; Yazdanian et al., 2024). Birds may also respond to management‐related changes in food availability through improved breeding success, but not necessarily increased local densities (Cordeiro Pereira, Mikusiński, & Storch, 2024; Grames et al., 2023). Thus, for the species we analyzed, abundance responses to management may be driven by factors other than overall food availability. For instance, settlement choices of breeding birds are strongly influenced by perceived predation risk (Fontaine & Martin, 2006), and higher canopy cover may improve nest concealment and reduce nest predation risk for midstory‐ and canopy‐nesters (Reidy et al., 2017), while also ensuring lower risk foraging opportunities (Suhonen, 1993).

Our findings contrast with those of Renner et al. (2024), who showed that the effects of forest structure on bird abundance were mediated by changes in arthropod biomass and abundance, in three regions of Germany. The reasons for this difference are unclear, but possible explanations are: the different forest types covered (mostly lowland forests), the pooling of abundances across all birds and invertebrate taxa, the inclusion of canopy invertebrates in that study, and the possible variation in the strength of bird–invertebrate relationships across years, given the high interannual variability of invertebrate populations (Haridas et al., 2016). Although our study used multiannual bird survey data, thereby obtaining more information to model relationships between forest predictors and bird abundances, neither study used multiannual data on invertebrates. Bottom‐up effects of invertebrate abundances on birds can manifest with a time lag of one or more years (e.g., Yazdanian et al., 2024). Thus, more research is needed to clarify in which circumstances effects of forest management on bird abundances may be mediated by invertebrate availability, ideally collecting both bird and invertebrate data across multiple years.

Despite the lack of support for a causal link between total abundance of invertebrates captured in FITs and the total abundance of foliage‐gleaning birds, both responded positively to broadleaf share. Several bird species and their potential prey groups also responded similarly to elevation and understory richness. Breeding birds often select territories without being able to assess how much food will be available later in the season (Hollander et al., 2013; Mäntylä et al., 2015), so that they may need to rely on habitat cues that are indicative of future food supplies (Chalfoun & Schmidt, 2012; McGrath et al., 2009). In these cases, bird densities would be more reflective of such cues than of actual invertebrate abundances, which are subject to strong fluctuations (e.g., due to weather). For example, in North America, foliage‐gleaning birds preferentially used dense shrubby vegetation in forest gaps throughout the year, but this only matched higher arthropod abundances for part of the year (Moorman et al., 2012), and that preference remained following experimental removal of arthropods (Champlin et al., 2009). In our case, the understory richness and broadleaf share (as well as elevation) may act as proximate cues of higher availability of invertebrates, explaining why birds respond to those forest predictors and not directly to invertebrate abundances.

### Study limitations and directions for future research

The trapping methods we used do not adequately sample Annelida (earthworms) or larvae of Lepidoptera (caterpillars) and other insect orders, which are important elements in the diet of thrushes and most foliage‐gleaners, respectively (von Glutz Blotzheim & Bauer, 1985; Nyffeler et al., 2018). Thus, some trophic‐mediated effects of management on birds may have remained undetected. Nevertheless, earthworms reach a much higher biomass in broadleaved than in spruce forests (Schelfhout et al., 2017; Verstraeten et al., 2018), and we did not detect any positive response of thrush species to broadleaf share. This hints that stand‐scale earthworm availability is not a limiting resource for thrush abundances in our study area. Regarding caterpillars, birds may respond to environmental cues that indicate low caterpillar supply by establishing larger territories, resulting in lower densities (Schöll et al., 2016). Since defoliating caterpillars are more abundant in broadleaf trees (Ampoorter et al., 2020; Hammond & Miller, 1998) and caterpillar biomass is lower at higher elevations (Schöll et al., 2016), responses of foliage‐gleaners to these predictors in our study may reflect their role as cues for caterpillar availability. Further research using a broader set of arthropod sampling techniques, as well as assessing resources for herbivorous and granivorous bird species, is needed to fully clarify links between bird abundances and food availability. In particular, sweep netting or branch clipping is more effective than FITs at capturing small prey with limited mobility (e.g., insect larvae and aphids) that are consumed by foliage‐gleaners, although the higher costs of this approach must be considered (Kent et al., 2019; Zandt, 1994).

Another limitation of this study is our focus on the forest floor and near‐ground vegetation layers, even though multiple foliage‐gleaner species often forage at higher levels, and invertebrate assemblages can strongly differ across forest layers (Floren & Schmidl, 2008). Thus, further insights could be gained by placing FITs higher (as in Renner et al., 2024) or using canopy fogging (as in Wildermuth et al., 2024). We also focused solely on the plot scale, as it reflects stand‐scale management practices, but birds and invertebrates may interact at larger scales. In our study area, Basile et al. (2021) determined that the abundance and composition of bird assemblages are strongly affected by landscape‐level factors, namely the cover of different forest types. While this is partly explained by bird movement patterns (Salgueiro et al., 2021), species with larger territory sizes (e.g., woodpeckers, thrushes, and pigeons) may respond numerically to food resources available beyond the stand scale (e.g., Rolstad et al., 1998). Increasingly detailed remote sensing data offer an opportunity for studies relating bird abundances with large‐scale availability of key foraging resources (e.g., Zielewska‐Büttner et al., 2018). Lastly, since a great majority of studies focus on bird abundance and habitat use in the breeding season, and European forest bird fauna contains a large proportion of resident species (Villard & Foppen, 2018), we urge for more research looking into the effects of multifunctional forestry practices outside the breeding season.

### Conclusions and management implications

Our findings indicate that retention and close‐to‐nature forestry practices, as implemented in Central European montane forests, affect the abundances of bird species spanning across nesting and foraging guilds. In particular, admixing broadleaf trees in stands has strong effects on bird abundances, as also found at the landscape scale (Basile et al., 2021) and may be of particular benefit to cavity‐nesting birds, in combination with the retention of large living broadleaf trees with unique microhabitats. Therefore, these management practices hold promise for the conservation of birds in Central European forests, although retention likely demands more time for its potential to be fully realized. Moreover, the direction of bird abundance responses to management varied, reinforcing that there is no “one‐size‐fits‐all” strategy for forest bird conservation and that a mosaic of differently structured stands, also including larger scale forest gaps, is needed to maximize diversity of forest birds at broader scales (Schall et al., 2018; Uhl et al., 2024).

In our study, despite both birds and invertebrates being affected by management, birds did not respond numerically to the abundances of the invertebrate groups we analyzed. Thus, management may be most effective at increasing bird abundances if it considers other limiting factors, such as availability of high‐quality nesting sites. Nonetheless, our results suggest that specific bird species and foraging guilds also respond to habitat features in forests (e.g., broadleaf share or higher understory richness) as cues for higher food availability. Therefore, management should still strive to promote high invertebrate abundances, not only to buffer against external stressors on invertebrate populations but also to prevent mismatches between habitat selection by birds and habitat quality (i.e., ecological traps, Battin, 2004). For instance, while close‐to‐nature forestry may promote invertebrate availability and attract higher bird abundances due to higher broadleaf shares, a closed canopy may be detrimental for birds and invertebrates, due to its negative effects on understory plant richness. It is then worth considering alternatives to single‐tree selection that create more canopy gaps, such as group selection cuts or irregular shelterwood (Uhl et al., 2024).

Our approach, measuring abundances of both birds and invertebrates, and using piecewise SEMs to infer causality on inter‐trophic relationships, showed promise to tease apart the mechanisms by which management affects both groups and their linkages. We encourage further research using this approach, which can also lay the groundwork for more focused experimental manipulations of forest structure and food supply, with a higher inferential power. Both observational and experimental approaches have important and complementary roles in building an evidence base on which to sustain multifunctional forestry.

## AUTHOR CONTRIBUTIONS

João Manuel Cordeiro Pereira conceptualized the study and conducted the statistical analyses. Ilse Storch secured funding for this study (through ConFoBi) and, alongside Grzegorz Mikusiński, supervised the doctoral dissertation of João Manuel Cordeiro Pereira, of which this study is a part. João Manuel Cordeiro Pereira, Sara Klingenfuß, Marco Basile, Julian Frey, and Grzegorz Mikusiński collected field data. João Manuel Cordeiro Pereira wrote the first manuscript draft, and all authors revised and contributed critically to subsequent drafts.

## CONFLICT OF INTEREST STATEMENT

The authors declare no conflicts of interest.

## Supporting information


Appendix S1.

Appendix S2.

Appendix S3.

Appendix S4.

Appendix S5.

Appendix S6.

Appendix S7.

## Data Availability

Data (Cordeiro Pereira et al., 2025) are available in Figshare at https://doi.org/10.6084/m9.figshare.28429760.
