# Supplementary material for: Disentangling the effects of multifunctional forestry practices on the abundances of birds and their invertebrate prey
Source: Ecol Appl. 2026 Mar 8;36(2):e70198. doi: 10.1002/eap.70198 (PMC12967705; doi:10.1002/eap.70198)
Supplement: Supplementary file 1 — Appendix S1. [file EAP-36-e70198-s002.pdf]

Appendix S1

Specifications and code for *N*-mixture models for bird abundance

Journal: Ecological Applications

Title: Disentangling the effects of multifunctional forestry practices on the abundances of birds and their invertebrate prey

Authors: João Manuel Cordeiro Pereira, Sara Klingenfuß, Marco Basile, Julian Frey, Grzegorz Mikusiński, Ilse Storch

Section S1: Prior specifications and parameters of Markov Chain Monte Carlo (MCMC) algorithms, for *N*-mixture models for bird abundance ran with R package *ubms* (Kellner et al., 2022), an interface for STAN (Stan Development Team, 2024). This encompasses all single-species models with Poisson distributions for abundance. Weakly informative priors were chosen according to Northrup & Gerber (2018).

MCMC algorithm: No U-Turn Sampler (NUTS) (Hoffman & Gelman, 2014)
Index of integration (K): 30
Prior for intercept coefficient of abundance sub-model: **Uniform (min = -10, max = 10)**
Prior for effect coefficients of abundance sub-model: **Uniform (min = -5, max = 5)**
Prior for coefficients of detection sub-model (intercept and effects): **Normal (mean = 0, variance = 2)**
Prior for standard deviation of random effects: **Gamma (shape = 1, rate = 1)**
Number of chains: 8
Number of iterations: 2,000
Warmup: 1,000
Size of final posterior sample: 8,000

Section S2: Prior specifications and MCMC parameters for *N*-mixture models for bird abundance ran with R package *spAbundance* (Doser et al., 2024). This encompasses all single-species models with negative binomial (NB) distribution for abundance. Similar weakly informative priors were used as those in Section S1.

MCMC algorithm: Custom adaptive algorithm from Roberts & Rosenthal (2009).
Prior for coefficients of abundance sub-model (intercept and effects): **Normal(mean = 0, variance = 100)**
Prior for coefficients of detection sub-model (intercept and effects): **Normal(mean = 0, variance = 2)**
Prior for negative binomial dispersion parameter (k): **Uniform(min = 0, max = 100)**
Prior for standard deviation of random effects: **Inverse Gamma(shape = 0.1, scale = 0.1)**

Tuning parameters: 0.5 for all parameters (default)
Number of chains: 3
Batch length: 25
Number of batches: 10,000
Target acceptance rate for Adaptive MCMC: 0.43 (default)
Number of batches at which acceptance rate is reported: 500
Total number of samples: 250,000
Burn-in: 100,000
Thinning frequency: 150
Size of final posterior sample: 3,000

Section S3: R code specifying and running *N*-mixture models for bird species' abundances. Code for each species is shown in the same order as in Fig. 2. Models for each species are named using the first three letters of each word in the scientific name, e.g. dry_mar for the black woodpecker, *Dryocopus martius*. For each species, code for two models is shown: one a full model, and another a model without fixed effects on its abundance component (model name followed by suffix "_null"), with the latter being used to generate estimates for our SEM analysis. The function used is *stan_pcount()* from R package *ubms* (Kellner et al., 2022) or *NMix()* from R package *spAbundance* (Doser et al., 2024), depending on whether Poisson or negative binomial distributions for abundance are used, respectively. Prior specifications and parameters of MCMC algorithms are similar across models, and summarized in Sections S1 and S2. The data file used for each model (with the same name as the model, followed by "_ubms" or "_data", depending on the package used) contains the bird counts and all observation-level or site-level covariate values, in the format required by each package. Legend for variables: *date*, ordinal date, *time*, minutes since sunrise, *observer*, observer (categorical), *plot_id*, unique plot ID (categorical), *Year*, year of sampling (treated categorically), *DBHMean*, mean tree diameter at breast height (DBH), *ndead_tf*, number of dead trees (log-transformed), *snag_dbh*, mean DBH of dead trees, *lying_dw_volume_tf*, volume of lying deadwood (log-transformed), *enl*, Effective Number of Layers (ENL), *DBH_sd*, standard deviation of tree DBH, *cancover*, canopy cover, *sCover_tf*, shrub-layer cover (square-root-transformed), *hCover*, herb-layer cover, *pc_broadleaf*, broadleaf share of basal area, *SR_trees*, tree species richness, *SR_understory*, understorey vascular plant richness, *avg_alt*, average elevation above sea level, *sd_slope*, standard deviation of terrain slope, *northness*, northness index.

```
dry_mar <- stan_pcount(~ date + time + (1|observer) ~ DBHMean + ndead_tf
+ snag_dbh + lying_dw_volume_tf + cancover + sCover_tf + hCover +
pc_broadleaf + SR_understory + avg_alt + sd_slope + northness,
                             data = dry_mar_ubms, K = 30,
                             prior_intercept_state = uniform(-10,10),
prior_coef_state = uniform(-5,5),
```

```
                           prior_intercept_det = normal(0, sqrt(2)),
prior_coef_det = normal(0, sqrt(2)),
                             prior_sigma = gamma(1,1),
                           chains = 8, iter = 2000, warmup = 1000, cores
= 8, seed = 123)
dry_mar_null <- stan_pcount(~ date + time + (1|observer) ~ 1,
                           data = dry_mar_ubms, K = 30,
                           prior_intercept_state = uniform(-10,10),
prior_coef_state = uniform(-5,5),
                           prior_intercept_det = normal(0, sqrt(2)),
prior_coef_det = normal(0, sqrt(2)),
                           prior_sigma = gamma(1,1),
                           chains = 8, iter = 2000, warmup = 1000, cores
= 8, seed = 123)

tro_tro <- stan_pcount(~ date + time + (1|observer) ~ DBHMean +
lying_dw_volume_tf + cancover + sCover_tf + hCover + pc_broadleaf +
SR_understory + avg_alt + sd_slope + northness,
                           data = tro_tro_ubms, K = 30,
                           prior_intercept_state = uniform(-10,10),
prior_coef_state = uniform(-5,5),
                           prior_intercept_det = normal(0, sqrt(2)),
prior_coef_det = normal(0, sqrt(2)),
                             prior_sigma = gamma(1,1),
                           chains = 8, iter = 2000, warmup = 1000, cores
= 8, seed = 123)
tro_tro_null <- stan_pcount(~ date + time + (1|observer) ~ 1,
                           data = tro_tro_ubms, K = 30,
                           prior_intercept_state = uniform(-10,10),
prior_coef_state = uniform(-5,5),
                           prior_intercept_det = normal(0, sqrt(2)),
prior_coef_det = normal(0, sqrt(2)),
                           prior_sigma = gamma(1,1),
                           chains = 8, iter = 2000, warmup = 1000, cores
= 8, seed = 123)

tur_phi <- stan_pcount(~ date + time + (1|observer) ~ DBHMean +
lying_dw_volume_tf + enl + DBH_sd + cancover + sCover_tf + hCover +
pc_broadleaf + SR_trees + SR_understory + avg_alt + sd_slope + northness,
                           data = tur_phi_ubms, K = 30,
                           prior_intercept_state = uniform(-10,10),
prior_coef_state = uniform(-5,5),
```

```
                              prior_intercept_det = normal(0, sqrt(2)),
prior_coef_det = normal(0, sqrt(2)),
                                prior_sigma = gamma(1,1),
                              chains = 8, iter = 2000, warmup = 1000, cores
= 8, seed = 123)
tur_phi_null <- stan_pcount(~ date + time + (1|observer) ~ 1,
                              data = tur_phi_ubms, K = 30,
                              prior_intercept_state = uniform(-10,10),
prior_coef_state = uniform(-5,5),
                              prior_intercept_det = normal(0, sqrt(2)),
prior_coef_det = normal(0, sqrt(2)),
                              prior_sigma = gamma(1,1),
                              chains = 8, iter = 2000, warmup = 1000, cores
= 8, seed = 123)

tur_vis <- stan_pcount(~ date + time + (1|observer) ~ DBHMean +
lying_dw_volume_tf + enl + DBH_sd + cancover + sCover_tf + hCover +
pc_broadleaf + SR_trees + SR_understory + avg_alt + sd_slope + northness,
                              data = tur_vis_ubms, K = 30,
                              prior_intercept_state = uniform(-10,10),
prior_coef_state = uniform(-5,5),
                              prior_intercept_det = normal(0, sqrt(2)),
prior_coef_det = normal(0, sqrt(2)),
                              prior_sigma = gamma(1,1),
                              chains = 8, iter = 2000, warmup = 1000, cores
= 8, seed = 123)
tur_vis_null <- stan_pcount(~ date + time + (1|observer) ~ 1,
                              data = tur_vis_ubms, K = 30,
                              prior_intercept_state = uniform(-10,10),
prior_coef_state = uniform(-5,5),
                              prior_intercept_det = normal(0, sqrt(2)),
prior_coef_det = normal(0, sqrt(2)),
                              prior_sigma = gamma(1,1),
                              chains = 8, iter = 2000, warmup = 1000, cores
= 8, seed = 123)

tur_mer <- stan_pcount(~ date + time + (1|observer) ~ DBHMean +
lying_dw_volume_tf + cancover + sCover_tf + hCover + pc_broadleaf +
SR_understory + avg_alt + sd_slope + northness,
                              data = tur_mer_ubms, K = 30,
                              prior_intercept_state = uniform(-10,10),
prior_coef_state = uniform(-5,5),
                              prior_intercept_det = normal(0, sqrt(2)),
prior_coef_det = normal(0, sqrt(2)) ,
```

```
                              prior_sigma = gamma(1,1),
                              chains = 8, iter = 2000, warmup = 1000, cores
= 8, seed = 123)
tur_mer_null <- stan_pcount(~ date + time + (1|observer) ~ 1,
                              data = tur_mer_ubms, K = 30,
                              prior_intercept_state = uniform(-10,10),
prior_coef_state = uniform(-5,5),
                              prior_intercept_det = normal(0, sqrt(2)),
prior_coef_det = normal(0, sqrt(2)),
                              prior_sigma = gamma(1,1),
                              chains = 8, iter = 2000, warmup = 1000, cores
= 8, seed = 123)

eri_rub <- stan_pcount(~ date + time + (1|observer) ~ DBHMean +
lying_dw_volume_tf + cancover + sCover_tf + hCover + pc_broadleaf +
SR_understory + avg_alt + sd_slope + northness,
                              data = eri_rub_ubms, K = 30,
                              prior_intercept_state = uniform(-10,10),
prior_coef_state = uniform(-5,5),
                              prior_intercept_det = normal(0, sqrt(2)),
prior_coef_det = normal(0, sqrt(2)),
                              prior_sigma = gamma(1,1),
                              chains = 8, iter = 2000, warmup = 1000, cores
= 8, seed = 123)
eri_rub_null <- stan_pcount(~ date + time + (1|observer) ~ 1,
                              data = eri_rub_ubms, K = 30,
                              prior_intercept_state = uniform(-10,10),
prior_coef_state = uniform(-5,5),
                              prior_intercept_det = normal(0, sqrt(2)),
prior_coef_det = normal(0, sqrt(2)),
                              prior_sigma = gamma(1,1),
                              chains = 8, iter = 2000, warmup = 1000, cores
= 8, seed = 123)

pru_mod <- stan_pcount(~ date + time + (1|observer) ~ DBHMean +
lying_dw_volume_tf + cancover + sCover_tf + hCover + pc_broadleaf +
SR_understory + avg_alt + sd_slope + northness,
                              data = pru_mod_ubms, K = 30,
                              prior_intercept_state = uniform(-10,10),
prior_coef_state = uniform(-5,5),
                              prior_intercept_det = normal(0, sqrt(2)),
prior_coef_det = normal(0, sqrt(2)),
                              prior_sigma = gamma(1,1),
```

```
                           chains = 8, iter = 2000, warmup = 1000, cores
= 8, seed = 123)
pru_mod_null <- stan_pcount(~ date + time + (1|observer) ~ 1,
                           data = pru_mod_ubms, K = 30,
                           prior_intercept_state = uniform(-10,10),
prior_coef_state = uniform(-5,5),
                           prior_intercept_det = normal(0, sqrt(2)),
prior_coef_det = normal(0, sqrt(2)),
                           prior_sigma = gamma(1,1),
                           chains = 8, iter = 2000, warmup = 1000, cores
= 8, seed = 123)

den_maj <- stan_pcount(~ date + time + (1|observer) ~ DBHMean + ndead_tf
+ snag_dbh + enl + DBH_sd + cancover + pc_broadleaf + SR_trees + avg_alt
+ northness + (1|Year),
                           data = den_maj_ubms, K = 30,
                           prior_intercept_state = uniform(-10,10),
prior_coef_state = uniform(-5,5),
                           prior_intercept_det = normal(0, sqrt(2)),
prior_coef_det = normal(0, sqrt(2)),
                           prior_sigma = gamma(1,1),
                           chains = 8, iter = 2000, warmup = 1000, cores
= 8, seed = 123)
den_maj_null <- stan_pcount(~ date + time + (1|observer) ~ 1 + (1|Year),
                           data = den_maj_ubms, K = 30,
                           prior_intercept_state = uniform(-10,10),
prior_coef_state = uniform(-5,5),
                           prior_intercept_det = normal(0, sqrt(2)),
prior_coef_det = normal(0, sqrt(2)),
                           prior_sigma = gamma(1,1),
                           chains = 8, iter = 2000, warmup = 1000, cores
= 8, seed = 123)

gar_gla <- NMix(
  abund.formula = ~ DBHMean + enl + DBH_sd + cancover + sCover_tf +
hCover + pc_broadleaf + SR_trees + SR_understory + avg_alt + northness,
  det.formula = ~ date + time + (1|observer),
  data = gar_gla_data,
  priors = list(
    alpha.normal = list(mean = 0, var = 2),
    beta.normal = list(mean = 0, var = 100),
    kappa.unif = c(0, 100),
    sigma.sq.mu.ig = list(0.1, 0.1),
    sigma.sq.p.ig = list(0.1, 0.1)
```

```
  ),
  tuning = list(
    beta = 0.5, alpha = 0.5, kappa = 0.5, alpha.star = 0.5, beta.star =
0.5),
  n.batch = 10000, batch.length = 25, accept.rate = 0.43,
  family = "NB",
  n.omp.threads = 1, n.report = 500, n.burn = 100000, n.thin = 150,
n.chains = 3
)
gar_gla_null <- NMix(
  abund.formula = ~ 1,
  det.formula = ~ date + time + (1|observer),
  data = gar_gla_data,
  priors = list(
    alpha.normal = list(mean = 0, var = 2),
    beta.normal = list(mean = 0, var = 100),
    kappa.unif = c(0, 100),
    sigma.sq.mu.ig = list(0.1, 0.1),
    sigma.sq.p.ig = list(0.1, 0.1)
  ),
  tuning = list(
    beta = 0.5, alpha = 0.5, kappa = 0.5, alpha.star = 0.5, beta.star =
0.5),
  n.batch = 10000, batch.length = 25, accept.rate = 0.43,
  family = "NB",
  n.omp.threads = 1, n.report = 500, n.burn = 100000, n.thin = 150,
n.chains = 3
)

per_ate <- stan_pcount(~ date + time + (1|observer) ~ DBHMean + ndead_tf
+ snag_dbh + enl + DBH_sd + cancover + sCover_tf + pc_broadleaf + avg_alt
+ northness + (1|Year),
                           data = per_ate_ubms, K = 30,
                           prior_intercept_state = uniform(-10,10),
prior_coef_state = uniform(-5,5),
                           prior_intercept_det = normal(0, sqrt(2)),
prior_coef_det = normal(0, sqrt(2)),
                           prior_sigma = gamma(1,1),
                           chains = 8, iter = 2000, warmup = 1000, cores
= 8, seed = 123)
per_ate_null <- stan_pcount(~ date + time + (1|observer) ~ 1 + (1|Year),
                           data = per_ate_ubms, K = 30,
                           prior_intercept_state = uniform(-10,10),
prior_coef_state = uniform(-5,5),
```

```
                           prior_intercept_det = normal(0, sqrt(2)),
prior_coef_det = normal(0, sqrt(2)),
                           prior_sigma = gamma(1,1),
                           chains = 8, iter = 2000, warmup = 1000, cores
= 8, seed = 123)

lop_cri <- stan_pcount(~ date + time + (1|observer) ~ DBHMean + ndead_tf
+ snag_dbh + enl + DBH_sd + cancover + sCover_tf + pc_broadleaf + avg_alt
+ northness,
                           data = lop_cri_ubms, K = 30,
                           prior_intercept_state = uniform(-10,10),
prior_coef_state = uniform(-5,5),
                           prior_intercept_det = normal(0, sqrt(2)),
prior_coef_det = normal(0, sqrt(2)),
                           prior_sigma = gamma(1,1),
                           chains = 8, iter = 2000, warmup = 1000, cores
= 8, seed = 123)
lop_cri_null <- stan_pcount(~ date + time + (1|observer) ~ 1,
                           data = lop_cri_ubms, K = 30,
                           prior_intercept_state = uniform(-10,10),
prior_coef_state = uniform(-5,5),
                           prior_intercept_det = normal(0, sqrt(2)),
prior_coef_det = normal(0, sqrt(2)),
                           prior_sigma = gamma(1,1),
                           chains = 8, iter = 2000, warmup = 1000, cores
= 8, seed = 123)

poe_pal <- stan_pcount(~ date + time + (1|observer) ~ DBHMean + ndead_tf
+ snag_dbh + enl + DBH_sd + cancover + sCover_tf + pc_broadleaf +
SR_trees + avg_alt + northness,
                           data = poe_pal_ubms, K = 30,
                           prior_intercept_state = uniform(-10,10),
prior_coef_state = uniform(-5,5),
                           prior_intercept_det = normal(0, sqrt(2)),
prior_coef_det = normal(0, sqrt(2)),
                           prior_sigma = gamma(1,1),
                           chains = 8, iter = 2000, warmup = 1000, cores
= 8, seed = 123,
                           control = list(adapt_delta = 0.99))
poe_pal_null <- NMix(
  abund.formula = ~ 1,
  det.formula = ~ date + time + (1|observer),
  data = poe_pal_data,
  priors = list(
```

```
    alpha.normal = list(mean = 0, var = 2),
    beta.normal = list(mean = 0, var = 100),
    kappa.unif = c(0, 100),
    sigma.sq.mu.ig = list(0.1, 0.1),
    sigma.sq.p.ig = list(0.1, 0.1)
  ),
  tuning = list(
    beta = 0.5, alpha = 0.5, kappa = 0.5, alpha.star = 0.5, beta.star =
0.5),
  n.batch = 10000, batch.length = 25, accept.rate = 0.43,
  family = "NB",
  n.omp.threads = 1, n.report = 500, n.burn = 100000, n.thin = 150,
n.chains = 3
)

cya_cae <- stan_pcount(~ date + time ~ DBHMean + ndead_tf + snag_dbh +
enl + DBH_sd + cancover + sCover_tf + pc_broadleaf + SR_trees + avg_alt +
northness + (1|Year),
                          data = cya_cae_ubms, K = 30,
                          prior_intercept_state = uniform(-10,10),
prior_coef_state = uniform(-5,5),
                          prior_intercept_det = normal(0, sqrt(2)),
prior_coef_det = normal(0, sqrt(2)),
                          prior_sigma = gamma(1,1),
                          chains = 8, iter = 4000, warmup = 2000, cores =
8, seed = 123,
                          control = list(adapt_delta = 0.95))
cya_cae_null <- NMix(
  abund.formula = ~ 1 + (1|Year),
  det.formula = ~ date + time,
  data = cya_cae_data,
  priors = list(
    alpha.normal = list(mean = 0, var = 2),
    beta.normal = list(mean = 0, var = 100),
    kappa.unif = c(0, 100),
    sigma.sq.mu.ig = list(0.1, 0.1),
    sigma.sq.p.ig = list(0.1, 0.1)
  ),
  tuning = list(
    beta = 0.5, alpha = 0.5, kappa = 0.5, alpha.star = 0.5, beta.star =
0.5),
  n.batch = 10000, batch.length = 25, accept.rate = 0.43,
  family = "NB",
```

```
  n.omp.threads = 1, n.report = 500, n.burn = 100000, n.thin = 150,
n.chains = 3
)

par_maj <- NMix(
  abund.formula = ~ DBHMean + ndead_tf + snag_dbh + enl + DBH_sd +
cancover + sCover_tf + hCover + pc_broadleaf + SR_trees + SR_understory +
avg_alt + northness,
  det.formula = ~ date + time + (1|observer),
  data = par_maj_data,
  priors = list(
    alpha.normal = list(mean = 0, var = 2),
    beta.normal = list(mean = 0, var = 100),
    kappa.unif = c(0, 100),
    sigma.sq.mu.ig = list(0.1, 0.1),
    sigma.sq.p.ig = list(0.1, 0.1)
  ),
  tuning = list(
    beta = 0.5, alpha = 0.5, kappa = 0.5, alpha.star = 0.5, beta.star =
0.5),
  n.batch = 10000, batch.length = 25, accept.rate = 0.43,
  family = "NB",
  n.omp.threads = 1, n.report = 500, n.burn = 100000, n.thin = 150,
n.chains = 3
)
par_maj_null <- NMix(
  abund.formula = ~ 1,
  det.formula = ~ date + time + (1|observer),
  data = par_maj_data,
  priors = list(
    alpha.normal = list(mean = 0, var = 2),
    beta.normal = list(mean = 0, var = 100),
    kappa.unif = c(0, 100),
    sigma.sq.mu.ig = list(0.1, 0.1),
    sigma.sq.p.ig = list(0.1, 0.1)
  ),
  tuning = list(
    beta = 0.5, alpha = 0.5, kappa = 0.5, alpha.star = 0.5, beta.star =
0.5),
  n.batch = 10000, batch.length = 25, accept.rate = 0.43,
  family = "NB",
  n.omp.threads = 1, n.report = 500, n.burn = 100000, n.thin = 150,
n.chains = 3
)
```

```
aeg_cau <- stan_pcount(~ date + time + (1|observer) ~ DBHMean + enl +
DBH_sd + cancover + sCover_tf + pc_broadleaf + SR_trees + avg_alt +
northness,
                           data = aeg_cau_ubms, K = 30,
                           prior_intercept_state = uniform(-10,10),
prior_coef_state = uniform(-5,5),
                           prior_intercept_det = normal(0, sqrt(2)),
prior_coef_det = normal(0, sqrt(2)),
                           prior_sigma = gamma(1,1),
                           chains = 8, iter = 2000, warmup = 1000, cores
= 8, seed = 123)
aeg_cau_null <- stan_pcount(~ date + time + (1|observer) ~ 1,
                           data = aeg_cau_ubms, K = 30,
                           prior_intercept_state = uniform(-10,10),
prior_coef_state = uniform(-5,5),
                           prior_intercept_det = normal(0, sqrt(2)),
prior_coef_det = normal(0, sqrt(2)),
                           prior_sigma = gamma(1,1),
                           chains = 8, iter = 2000, warmup = 1000, cores
= 8, seed = 123)

phy_col <- stan_pcount(~ date + time + (1|observer) ~ DBHMean +
lying_dw_volume_tf + enl + DBH_sd + cancover + sCover_tf + hCover +
pc_broadleaf + SR_trees + SR_understory + avg_alt + sd_slope + northness,
                           data = phy_col_ubms, K = 30,
                           prior_intercept_state = uniform(-10,10),
prior_coef_state = uniform(-5,5),
                           prior_intercept_det = normal(0, sqrt(2)),
prior_coef_det = normal(0, sqrt(2)),
                           prior_sigma = gamma(1,1),
                           chains = 8, iter = 2000, warmup = 1000, cores
= 8, seed = 123)
phy_col_null <- stan_pcount(~ date + time + (1|observer) ~ 1,
                           data = phy_col_ubms, K = 30,
                           prior_intercept_state = uniform(-10,10),
prior_coef_state = uniform(-5,5),
                           prior_intercept_det = normal(0, sqrt(2)),
prior_coef_det = normal(0, sqrt(2)),
                           prior_sigma = gamma(1,1),
                           chains = 8, iter = 2000, warmup = 1000, cores
= 8, seed = 123)
```

```
syl_atr <- stan_pcount(~ date + time + (1|observer) ~ DBHMean + enl +
DBH_sd + cancover + sCover_tf + pc_broadleaf + SR_trees + avg_alt +
northness,
                           data = syl_atr_ubms, K = 30,
                           prior_intercept_state = uniform(-10,10),
prior_coef_state = uniform(-5,5),
                           prior_intercept_det = normal(0, sqrt(2)),
prior_coef_det = normal(0, sqrt(2)),
                           prior_sigma = gamma(1,1),
                           chains = 8, iter = 2000, warmup = 1000, cores
= 8, seed = 123)
syl_atr_null <- stan_pcount(~ date + time + (1|observer) ~ 1,
                           data = syl_atr_ubms, K = 30,
                           prior_intercept_state = uniform(-10,10),
prior_coef_state = uniform(-5,5),
                           prior_intercept_det = normal(0, sqrt(2)),
prior_coef_det = normal(0, sqrt(2)),
                           prior_sigma = gamma(1,1),
                           chains = 8, iter = 2000, warmup = 1000, cores
= 8, seed = 123)

reg_ign <- stan_pcount(~ date + time + (1|observer) ~ DBHMean + enl +
DBH_sd + cancover + sCover_tf + pc_broadleaf + avg_alt + northness,
                           data = reg_ign_ubms, K = 30,
                           prior_intercept_state = uniform(-10,10),
prior_coef_state = uniform(-5,5),
                           prior_intercept_det = normal(0, sqrt(2)),
prior_coef_det = normal(0, sqrt(2)),
                           prior_sigma = gamma(1,1),
                           chains = 8, iter = 2000, warmup = 1000, cores
= 8, seed = 123)
reg_ign_null <- stan_pcount(~ date + time + (1|observer) ~ 1,
                           data = reg_ign_ubms, K = 30,
                           prior_intercept_state = uniform(-10,10),
prior_coef_state = uniform(-5,5),
                           prior_intercept_det = normal(0, sqrt(2)),
prior_coef_det = normal(0, sqrt(2)),
                           prior_sigma = gamma(1,1),
                           chains = 8, iter = 2000, warmup = 1000, cores
= 8, seed = 123)

reg_reg <- stan_pcount(~ date + time + (1|observer) ~ DBHMean + enl +
DBH_sd + cancover + sCover_tf + pc_broadleaf + avg_alt + northness +
(1|Year),
```

```
                           data = reg_reg_ubms, K = 30,
                           prior_intercept_state = uniform(-10,10),
prior_coef_state = uniform(-5,5),
                           prior_intercept_det = normal(0, sqrt(2)),
prior_coef_det = normal(0, sqrt(2)),
                           prior_sigma = gamma(1,1),
                           chains = 8, iter = 2000, warmup = 1000, cores
= 8, seed = 123)
reg_reg_null <- stan_pcount(~ date + time + (1|observer) ~ 1 + (1|Year),
                           data = reg_reg_ubms, K = 30,
                           prior_intercept_state = uniform(-10,10),
prior_coef_state = uniform(-5,5),
                           prior_intercept_det = normal(0, sqrt(2)),
prior_coef_det = normal(0, sqrt(2)),
                           prior_sigma = gamma(1,1),
                           chains = 8, iter = 2000, warmup = 1000, cores
= 8, seed = 123)

fri_coe <- stan_pcount(~ date + time + (1|observer) ~ DBHMean + enl +
DBH_sd + cancover + sCover_tf + hCover + pc_broadleaf + SR_trees +
SR_understory + avg_alt + northness,
                           data = fri_coe_ubms, K = 30,
                           prior_intercept_state = uniform(-10,10),
prior_coef_state = uniform(-5,5),
                           prior_intercept_det = normal(0, sqrt(2)),
prior_coef_det = normal(0, sqrt(2)),
                           prior_sigma = gamma(1,1),
                           chains = 8, iter = 2000, warmup = 1000, cores
= 8, seed = 123)
fri_coe_null <- stan_pcount(~ date + time + (1|observer) ~ 1,
                           data = fri_coe_ubms, K = 30,
                           prior_intercept_state = uniform(-10,10),
prior_coef_state = uniform(-5,5),
                           prior_intercept_det = normal(0, sqrt(2)),
prior_coef_det = normal(0, sqrt(2)),
                           prior_sigma = gamma(1,1),
                           chains = 8, iter = 2000, warmup = 1000, cores
= 8, seed = 123)

coc_coc <- stan_pcount(~ date + time + (1|observer) ~ DBHMean + enl +
DBH_sd + cancover + sCover_tf + hCover + pc_broadleaf + SR_trees +
SR_understory + avg_alt + northness,
                           data = coc_coc_ubms, K = 30,
```

```
                           prior_intercept_state = uniform(-10,10),
prior_coef_state = uniform(-5,5),
                           prior_intercept_det = normal(0, sqrt(2)),
prior_coef_det = normal(0, sqrt(2)),
                           prior_sigma = gamma(1,1),
                           chains = 8, iter = 2000, warmup = 1000, cores
= 8, seed = 123)
coc_coc_null <- stan_pcount(~ date + time + (1|observer) ~ 1,
                           data = coc_coc_ubms, K = 30,
                           prior_intercept_state = uniform(-10,10),
prior_coef_state = uniform(-5,5),
                           prior_intercept_det = normal(0, sqrt(2)),
prior_coef_det = normal(0, sqrt(2)),
                           prior_sigma = gamma(1,1),
                           chains = 8, iter = 2000, warmup = 1000, cores
= 8, seed = 123)

sit_eur <- stan_pcount(~ date + time + (1|observer) ~ DBHMean + ndead_tf
+ snag_dbh + enl + DBH_sd + cancover + pc_broadleaf + SR_trees + avg_alt
+ northness,
                           data = sit_eur_ubms, K = 30,
                           prior_intercept_state = uniform(-10,10),
prior_coef_state = uniform(-5,5),
                           prior_intercept_det = normal(0, sqrt(2)),
prior_coef_det = normal(0, sqrt(2)),
                           prior_sigma = gamma(1,1),
                           chains = 8, iter = 2000, warmup = 1000, cores
= 8, seed = 123)
sit_eur_null <- stan_pcount(~ date + time + (1|observer) ~ 1,
                           data = sit_eur_ubms, K = 30,
                           prior_intercept_state = uniform(-10,10),
prior_coef_state = uniform(-5,5),
                           prior_intercept_det = normal(0, sqrt(2)),
prior_coef_det = normal(0, sqrt(2)),
                           prior_sigma = gamma(1,1),
                           chains = 8, iter = 2000, warmup = 1000, cores
= 8, seed = 123)

cer_bra <- stan_pcount(~ date + time + (1|observer) ~ DBHMean + ndead_tf
+ snag_dbh + enl + DBH_sd + cancover + pc_broadleaf + SR_trees + avg_alt
+ northness,
                           data = cer_bra_ubms, K = 30,
```

```
                            prior_intercept_state = uniform(-10,10),
prior_coef_state = uniform(-5,5),
                            prior_intercept_det = normal(0, sqrt(2)),
prior_coef_det = normal(0, sqrt(2)),
                            prior_sigma = gamma(1,1),
                            chains = 8, iter = 4000, warmup = 2000, cores
= 8, seed = 123)
cer_bra_null <- stan_pcount(~ date + time + (1|observer) ~ 1,
                            data = cer_bra_ubms, K = 30,
                            prior_intercept_state = uniform(-10,10),
prior_coef_state = uniform(-5,5),
                            prior_intercept_det = normal(0, sqrt(2)),
prior_coef_det = normal(0, sqrt(2)),
                            prior_sigma = gamma(1,1),
                            chains = 8, iter = 2000, warmup = 1000, cores
= 8, seed = 123)

cer_fam <- stan_pcount(~ date + time + (1|observer) ~ DBHMean + ndead_tf
+ snag_dbh + enl + DBH_sd + cancover + pc_broadleaf + SR_trees + avg_alt
+ northness + (1|Year),
                            data = cer_fam_ubms, K = 30,
                            prior_intercept_state = uniform(-10,10),
prior_coef_state = uniform(-5,5),
                            prior_intercept_det = normal(0, sqrt(2)),
prior_coef_det = normal(0, sqrt(2)),
                            prior_sigma = gamma(1,1),
                            chains = 8, iter = 2000, warmup = 1000, cores
= 8, seed = 123)
cer_fam_null <- stan_pcount(~ date + time + (1|observer) ~ 1 + (1|Year),
                            data = cer_fam_ubms, K = 30,
                            prior_intercept_state = uniform(-10,10),
prior_coef_state = uniform(-5,5),
                            prior_intercept_det = normal(0, sqrt(2)),
prior_coef_det = normal(0, sqrt(2)),
                            prior_sigma = gamma(1,1),
                            chains = 8, iter = 2000, warmup = 1000, cores
= 8, seed = 123)

col_oen <- stan_pcount(~ date + time ~ DBHMean + ndead_tf + snag_dbh +
cancover + pc_broadleaf + avg_alt + northness,
                            data = col_oen_ubms, K = 30,
                            prior_intercept_state = uniform(-10,10),
prior_coef_state = uniform(-5,5),
```

```
                           prior_intercept_det = normal(0, sqrt(2)),
prior_coef_det = normal(0, sqrt(2)),
                           chains = 8, iter = 2000, warmup = 1000, cores
= 8, seed = 123)
col_oen_null <- stan_pcount(~ date + time ~ 1,
                           data = col_oen_ubms, K = 30,
                           prior_intercept_state = uniform(-10,10),
prior_coef_state = uniform(-5,5),
                           prior_intercept_det = normal(0, sqrt(2)),
prior_coef_det = normal(0, sqrt(2)),
                           chains = 8, iter = 2000, warmup = 1000, cores
= 8, seed = 123)

col_pal <- NMix(
  abund.formula = ~ DBHMean + enl + DBH_sd + cancover + sCover_tf +
hCover + pc_broadleaf + SR_understory + SR_trees + avg_alt,
  det.formula = ~ date + time + (1|observer),
  data = col_pal_data,
  priors = list(
    alpha.normal = list(mean = 0, var = 2),
    beta.normal = list(mean = 0, var = 100),
    kappa.unif = c(0, 100),
    sigma.sq.mu.ig = list(0.1, 0.1),
    sigma.sq.p.ig = list(0.1, 0.1)
  ),
  tuning = list(
    beta = 0.5, alpha = 0.5, kappa = 0.5, alpha.star = 0.5, beta.star =
0.5),
  n.batch = 10000, batch.length = 25, accept.rate = 0.43,
  family = "NB",
  n.omp.threads = 1, n.report = 500, n.burn = 100000, n.thin = 150,
n.chains = 3
)
col_pal_null <- NMix(
  abund.formula = ~ 1,
  det.formula = ~ date + time + (1|observer),
  data = col_pal_data,
  priors = list(
    alpha.normal = list(mean = 0, var = 2),
    beta.normal = list(mean = 0, var = 100),
    kappa.unif = c(0, 100),
    sigma.sq.mu.ig = list(0.1, 0.1),
    sigma.sq.p.ig = list(0.1, 0.1)
  ),
```

```
  tuning = list(
    beta = 0.5, alpha = 0.5, kappa = 0.5, alpha.star = 0.5, beta.star =
0.5),
  n.batch = 10000, batch.length = 25, accept.rate = 0.43,
  family = "NB",
  n.omp.threads = 1, n.report = 500, n.burn = 100000, n.thin = 150,
n.chains = 3
)

pyr_pyr <- stan_pcount(~ date + time + (1|observer) ~ DBHMean + enl +
DBH_sd + cancover + sCover_tf + hCover + pc_broadleaf + SR_trees +
SR_understory + avg_alt + northness + (1|Year),
                           data = pyr_pyr_ubms, K = 30,
                           prior_intercept_state = uniform(-10,10),
prior_coef_state = uniform(-5,5),
                           prior_intercept_det = normal(0, sqrt(2)),
prior_coef_det = normal(0, sqrt(2)),
                           prior_sigma = gamma(1,1),
                           chains = 8, iter = 2000, warmup = 1000, cores
= 8, seed = 123)
pyr_pyr_null <- stan_pcount(~ date + time + (1|observer) ~ 1 + (1|Year),
                           data = pyr_pyr_ubms, K = 30,
                           prior_intercept_state = uniform(-10,10),
prior_coef_state = uniform(-5,5),
                           prior_intercept_det = normal(0, sqrt(2)),
prior_coef_det = normal(0, sqrt(2)),
                           prior_sigma = gamma(1,1),
                           chains = 8, iter = 2000, warmup = 1000, cores
= 8, seed = 123)

chl_chl <- stan_pcount(~ date + time ~ DBHMean + enl + DBH_sd + cancover
+ sCover_tf + hCover + pc_broadleaf + SR_trees + SR_understory + avg_alt
+ northness,
                          data = chl_chl_ubms, K = 30,
                          prior_intercept_state = uniform(-10,10),
prior_coef_state = uniform(-5,5),
                          prior_intercept_det = normal(0, sqrt(2)),
prior_coef_det = normal(0, sqrt(2)),
                          chains = 8, iter = 2000, warmup = 1000, cores =
8, seed = 123, control = list(adapt_delta = 0.95))
chl_chl_null <- stan_pcount(~ date + time ~ 1,
                          data = chl_chl_ubms, K = 30,
                          prior_intercept_state = uniform(-10,10),
prior_coef_state = uniform(-5,5),
```

```
                          prior_intercept_det = normal(0, sqrt(2)),
prior_coef_det = normal(0, sqrt(2)),
                          chains = 8, iter = 2000, warmup = 1000, cores =
8, seed = 123)

lox_cur <- NMix(
  abund.formula = ~ DBHMean + enl + DBH_sd + cancover + pc_broadleaf +
avg_alt + northness + (1|Year) + (1|plot_id),
  det.formula = ~ date + time + (1|observer),
  data = lox_cur_data,
  priors = list(
    alpha.normal = list(mean = 0, var = 2),
    beta.normal = list(mean = 0, var = 100),
    kappa.unif = c(0, 100),
    sigma.sq.mu.ig = list(0.1, 0.1),
    sigma.sq.p.ig = list(0.1, 0.1)
  ),
  tuning = list(
    beta = 0.5, alpha = 0.5, kappa = 0.5, alpha.star = 0.5, beta.star =
0.5),
  n.batch = 10000, batch.length = 25, accept.rate = 0.43,
  family = "NB",
  n.omp.threads = 1, n.report = 500, n.burn = 100000, n.thin = 150,
n.chains = 3
)

lox_cur_null <- NMix(
  abund.formula = ~ 1 + (1|Year) + (1|plot_id),
  det.formula = ~ date + time + (1|observer),
  data = lox_cur_data,
  priors = list(
    alpha.normal = list(mean = 0, var = 2),
    beta.normal = list(mean = 0, var = 100),
    kappa.unif = c(0, 100),
    sigma.sq.mu.ig = list(0.1, 0.1),
    sigma.sq.p.ig = list(0.1, 0.1)
  ),
  tuning = list(
    beta = 0.5, alpha = 0.5, kappa = 0.5, alpha.star = 0.5, beta.star =
0.5),
  n.batch = 10000, batch.length = 25, accept.rate = 0.43,
  family = "NB",
  n.omp.threads = 1, n.report = 500, n.burn = 100000, n.thin = 150,
n.chains = 3
```

```
)

spi_spi <- stan_pcount(~ date + time + (1|observer) ~ DBHMean + enl +
DBH_sd + cancover + pc_broadleaf + SR_trees + avg_alt + northness +
(1|Year),
                           data = spi_spi_ubms, K = 30,
                           prior_intercept_state = uniform(-10,10),
prior_coef_state = uniform(-5,5),
                           prior_intercept_det = normal(0, sqrt(2)),
prior_coef_det = normal(0, sqrt(2)),
                           prior_sigma = gamma(1,1),
                           chains = 8, iter = 2000, warmup = 1000, cores
= 8, seed = 123)
spi_spi_null <- stan_pcount(~ date + time + (1|observer) ~ 1 + (1|Year),
                           data = spi_spi_ubms, K = 30,
                           prior_intercept_state = uniform(-10,10),
prior_coef_state = uniform(-5,5),
                           prior_intercept_det = normal(0, sqrt(2)),
prior_coef_det = normal(0, sqrt(2)),
                           prior_sigma = gamma(1,1),
                           chains = 8, iter = 2000, warmup = 1000, cores
= 8, seed = 123)
```

References

Doser, J. W., Finley, A. O., Kéry, M., & Zipkin, E. F. (2024). spAbundance: An R package for single-species and multi-species spatially explicit abundance models. *Methods in Ecology and Evolution*, *15(6),* 1024-1033. https://doi.org/10.1111/2041-210X.14332

Hoffman, M. D., & Gelman, A. (2014). The No-U-Turn Sampler: Adaptively Setting Path Lengths in Hamiltonian Monte Carlo. *Journal of Machine Learning Research, 15(47)*, 1593–1623.

Kellner, K. F., Fowler, N. L., Petroelje, T. R., Kautz, T. M., Beyer, D. E., & Belant, J. L. (2022). ubms: An R package for fitting hierarchical occupancy and N-mixture abundance models in a Bayesian framework. *Methods in Ecology and Evolution*, *13*(3), 577–584. https://doi.org/10.1111/2041-210X.13777

Northrup, J. M., & Gerber, B. D. (2018). A comment on priors for Bayesian occupancy models. *PLOS ONE*, *13*(2), e0192819. https://doi.org/10.1371/journal.pone.0192819

Roberts, G. O. & Rosenthal, J. S. (2009). Examples of Adaptive MCMC. *Journal of Computational and Graphical Statistics*, *18(2),* 349–367. https://doi.org/10.1198/jcgs.2009.06134

Stan Development Team. (2024). *Stan Modeling Language Users Guide and Reference Manual, Version 2.34* [Computer software].
