# Supplementary material for: Disentangling the effects of multifunctional forestry practices on the abundances of birds and their invertebrate prey
Source: Ecol Appl. 2026 Mar 8;36(2):e70198. doi: 10.1002/eap.70198 (PMC12967705; doi:10.1002/eap.70198)
Supplement: Supplementary file 2 — Appendix S2. [file EAP-36-e70198-s003.pdf]

Appendix S2

Specifications and key outputs of Structural Equation Models (SEMs)

Journal: Ecological Applications

Title: Disentangling the effects of multifunctional forestry practices on the abundances of birds and their invertebrate prey

Authors: João Manuel Cordeiro Pereira, Sara Klingenfuß, Marco Basile, Julian Frey, Grzegorz Mikusiński, Ilse Storch

---

Section S1: Specified models (response on left side of formula and predictors on the right side), error distributions and link functions used to build a global SEM of ground-foraging birds and epigeal invertebrates, based on 2020 data in 57 plots[a]. This is followed by initial SEM goodness-of-fit, and explained variation (R^2) for each response variable. We also show a list of free correlations added based on directed separation tests, and the goodness-of-fit of SEM after addition of free correlations (final SEM), as well as after removal of the path linking invertebrate abundance to bird abundance.

Total abundance of ground-foragers (*Quasipoisson, log-link*) ~ mean DBH + lying DW volume + ENL + s.d. DBH + canopy cover + shrub cover + herb cover + broadleaf share + understorey SR + altitude + northness + total abundance of epigeal invertebrates[b]

Total abundance of epigeal invertebrates[b] (*Gamma, log-link*) ~ snag DBH + lying DW volume + canopy cover + shrub cover + herb cover + broadleaf share + understorey SR + altitude + s.d. slope + northness

Snag DBH (*Gaussian*, *linear model*) ~ mean DBH
Model: Linear model

ENL (*Gaussian*, *linear model*) ~ mean DBH + s.d. DBH + shrub cover + canopy cover + broadleaf share

Shrub cover[c,d] (*Beta, logit-link*) ~ mean DBH + canopy cover + broadleaf share

Herb cover[d] (*Beta, logit-link*) ~ mean DBH + canopy cover + broadleaf share

---

[a] For the SEMs we could only use plots where bird surveys and invertebrate trapping had occurred in the same year, resulting in 57 plots for pitfall traps in 2020, and 97 plots for flight interception traps in 2017.
[b] Total abundance of epigeal invertebrates excluded Hymenoptera, as most specimens in this group were wood ants (*Formica* spp.), not often taken as prey by birds. Total abundance of epigeal invertebrates was adjusted for sampling effort by dividing by trap-days.
[c] Shrub cover is not square-root-transformed, as it is used both as a response variable and a predictor in the SEM.
[d] Proportion variables (shrub cover, herb cover, canopy cover) were transformed as per Smithson & Verkuilen (2006), so all values are in an open interval from 0 to 1 and a beta regression could be used.

Understorey SR (*Negative binomial, log-link*) ~ herb cover + quadratic term(herb cover) + shrub cover + altitude + northness + broadleaf share

Global goodness-of-fit (initial model):
Fisher's C = 171.302, p = 0, df = 88
AICc = 797.67

R^2 for response variables (initial model):
- Total abundance of ground-foragers: 0.18
- Total abundance of epigeal invertebrates: 0.39
- Snag DBH: 0.01
- ENL: 0.43
- Shrub cover: 0.19
- Herb cover: 0.46
- Understorey SR: 0.54

Free correlations added:
lying DW volume ↔ snag DBH
s.d. DBH ↔ shrub cover
s.d. DBH ↔ snag DBH
altitude ↔ ENL
s.d. slope ↔ Total abundance of ground-foragers
herb cover ↔ Total abundance of ground-foragers
ENL ↔ herb cover
ENL ↔ Total abundance of epigeal invertebrates
snag DBH ↔ shrub cover

Global goodness-of-fit (final model, with added free correlations):
Fisher's C = 76.276, p = 0.343, df = 72
AICc = 702.64

Global goodness-of-fit (removing bird-invertebrate path from final model):
Fisher's C = 79.635, p = 0.306, df = 74
AICc = 642.968
ΔAICc = 59.672 (vs. final model)

Section S2: Same as Section S1, but concerning a global SEM of foliage-gleaning/bark-foraging birds and FIT invertebrates, based on 2017 data in 97 plots[a].

Total abundance of foliage-gleaners[e] (*Quasipoisson, log-link*) ~ mean DBH + lying DW volume + s.d. DBH + canopy cover + shrub cover + broadleaf share + tree SR + understorey SR + altitude + northness + total abundance of invertebrates from FITs[f]

Total abundance of bark-foragers (*Quasipoisson, log-link*) ~ snag DBH + s.d. DBH + canopy cover + broadleaf share + altitude + total abundance of invertebrates from FITs[f]

Total abundance of invertebrates from FITs[f,g] (*Gamma, log-link*) ~ mean DBH + no. snags + ENL + s.d. DBH + canopy cover + shrub cover + broadleaf share + tree SR + altitude + northness

Snag DBH (*Gaussian, linear model*) ~ mean DBH

ENL (*Gaussian, linear model*) ~ mean DBH + s.d. DBH + shrub cover + broadleaf share

Canopy cover[d] (*Beta, log-link*) ~ no. snags

Shrub cover[c,d] (*Beta, log-link*) ~ mean DBH + canopy cover + broadleaf share

Understorey SR (*Negative binomial, log-link*) ~ canopy cover + shrub cover + altitude + northness + broadleaf share

Tree SR (*Quasipoisson, log-link*) ~ altitude

Global goodness-of-fit (initial model):
Fisher's C = 276.907, p = 0, df = 114
AICc = 600.24

R^2 for response variables (initial model):
- Total abundance of foliage-gleaners: 0.32
- Total abundance of bark-foragers: 0.23
- Total abundance of invertebrates from FITs: 0.37
- Snag DBH: 0.00
- ENL: 0.51
- Canopy cover: 0.06
- Shrub cover: 0.16
- Understorey SR: 0.12
- Tree SR: 0.48

---

[e] Total abundance of foliage-gleaning birds excluded the Eurasian Jay (*Garrulus glandarius*), for which abundance estimates were unreliably high (> 20 inds. per plot).
[f] Total abundance of invertebrates from FITs was adjusted for sampling effort by dividing by the number of days in which traps were active in each plot.
[g] So we could use Gamma models for invertebrate abundance, values of 0 abundance were replaced with a very small number (10 times smaller than the next-largest value).

Free correlations added:
mean DBH ↔ tree SR
mean DBH ↔ total abundance of bark-foragers
lying DW volume ↔ canopy cover
lying DW volume ↔ snag DBH
s.d. DBH ↔ snag DBH
s.d. DBH ↔ shrub cover
broadleaf share ↔ canopy cover
broadleaf share ↔ snag DBH
altitude ↔ canopy cover
altitude ↔ ENL
no. snags ↔ shrub cover
canopy cover ↔ snag DBH
tree SR ↔ understorey SR
shrub cover ↔ total abundance of bark-foragers
snag DBH ↔ shrub cover

Global goodness-of-fit (final model, with added free correlations):
Fisher's C = 70.57, p = 0.852, df = 84
AICc = 393.903

Global goodness-of-fit (removing bird-invertebrate paths from final model):
Fisher’s C = 99.163, p = 0.095, df = 82
AICc = 256.559
ΔAICc = 137.344 (vs. final model)

Section S3: Same as Section S1, but concerning a SEM for small ground-foraging bird species and epigeal spiders, based on 2020 data in 57 plots[a].

Total abundance of small ground-foragers (*Quasipoisson, log-link*) ~ mean DBH + lying DW volume + canopy cover + shrub cover + herb cover + broadleaf share + understorey SR + altitude + northness + abundance of epigeal spiders

Abundance of epigeal spiders[g] (*Gamma, log-link*) ~ canopy cover + understorey SR + altitude

Shrub cover[c,d] ~ mean DBH (*Beta, logit-link*) + canopy cover + broadleaf share

Herb cover[d] ~ (*Beta, logit-link*) mean DBH + canopy cover + broadleaf share

Understorey SR (*Negative binomial, log-link*) ~ herb cover + quadratic term(herb cover) + shrub cover + altitude + northness + broadleaf share

Global goodness-of-fit (initial model):
Fisher's C = 37.428, p = 0.496, df = 38
AICc = 194.325

R^2 for response variables (initial model):
- Total abundance of small ground-foragers: 0.29
- Abundance of epigeal spiders: 0.18
- Shrub cover: 0.19
- Herb cover: 0.47
- Understorey SR: 0.54

No free correlations added.

Global goodness-of-fit (removing bird-invertebrate path from final model):
Fisher's C = 38.266, p = 0.548, df = 40
AICc = 185.599
ΔAICc = 8.726 (vs. final model)

References

Smithson, M., & Verkuilen, J. (2006). A better lemon squeezer? Maximum-likelihood regression with beta-distributed dependent variables. *Psychological Methods*, *11*(1), 54–71. https://doi.org/10.1037/1082-989X.11.1.54
