# Supplementary material for: Disentangling the effects of multifunctional forestry practices on the abundances of birds and their invertebrate prey
Source: Ecol Appl. 2026 Mar 8;36(2):e70198. doi: 10.1002/eap.70198 (PMC12967705; doi:10.1002/eap.70198)
Supplement: Supplementary file 3 — Appendix S3. [file EAP-36-e70198-s001.pdf]

Appendix S3

List of detected bird species

Journal: Ecological Applications

Title: Disentangling the effects of multifunctional forestry practices on the abundances of birds and their invertebrate prey

Authors: João Manuel Cordeiro Pereira, Sara Klingenfuß, Marco Basile, Julian Frey, Grzegorz Mikusiński, Ilse Storch

Table S1: All bird species detected in bird surveys across the 135 study plots (< 50 m from the plot center) over all years (2017-2022), including the average count for each species across all plots and years, the number of plots where the species was detected, and whether it was selected as a focal species for this study. The list follows the taxonomy of the International Ornithological Congress (IOC), version 13.2 (Gill, Donsker, and Rasmussen, 2023).

Species (scientific)	Species (English)	Average count 2017-2022	No. plots 2017-2022	Focal species
Anas platyrhynchos	Mallard	0.0028	1	
Apus apus	Common Swift	0.0139	4	
Columba oenas	Stock Dove	0.0390	11	✓
Columba palumbus	Common Wood Pigeon	0.6178	122	✓
Scolopax rusticola	Eurasian Woodcock	0.0014	1	
Pernis apivorus	European Honey Buzzard	0.0014	1	
Accipiter nisus	Eurasian Sparrowhawk	0.0111	7	
Accipiter gentilis	Northern Goshawk	0.0056	4	
Milvus milvus	Red Kite	0.0614	22	
Milvus migrans	Black Kite	0.0070	4	
Buteo buteo	Common Buzzard	0.1227	55	
Aegolius funereus	Boreal Owl	0.0014	1	
Glaucidium passerinum	Eurasian Pygmy Owl	0.0070	5	
Strix aluco	Tawny Owl	0.0084	5	
Jynx torquilla	Eurasian Wryneck	0.0014	1	
Picoides tridactylus	Eurasian Three-toed Woodpecker	0.0056	2	
Dendrocoptes medius	Middle Spotted Woodpecker	0.0084	6	

Dryobates minor	Lesser Spotted Woodpecker	0.0028	2	
Dendrocopos major	Great Spotted Woodpecker	0.4602	120	✓
Dryocopus martius	Black Woodpecker	0.0893	48	✓
Picus viridis	European Green Woodpecker	0.0125	5	
Picus canus	Grey-headed Woodpecker	0.0070	5	
Falco tinnunculus	Eurasian Kestrel	0.0014	1	
Falco peregrinus	Peregrine Falcon	0.0028	2	
Oriolus oriolus	Eurasian Oriole	0.0014	1	
Lanius collurio	Red-backed Shrike	0.0028	2	
Garrulus glandarius	Eurasian Jay	0.3863	115	✓
Nucifraga caryocatactes	Spotted Nutcracker	0.0265	13	✓
Corvus corone	Carrion Crow	0.1827	64	
Corvus corax	Northern Raven	0.0446	24	
Periparus ater	Coal Tit	1.1423	135	✓
Lophophanes cristatus	European Crested Tit	0.5370	122	✓
Poecile palustris	Marsh Tit	0.1227	49	✓
Poecile montanus	Willow Tit	0.0181	10	
Cyanistes caeruleus	Eurasian Blue Tit	0.1953	62	✓
Parus major	Great Tit	0.5844	124	✓
Lullula arborea	Woodlark	0.0014	1	
Alauda arvensis	Eurasian Skylark	0.0014	1	
Hirundo rustica	Barn Swallow	0.0084	4	
Delichon urbicum	Common House Martin	0.0042	2	
Aegithalos caudatus	Long-tailed Tit	0.0795	36	✓
Phylloscopus sibilatrix	Wood Warbler	0.0167	8	
Phylloscopus trochilus	Willow Warbler	0.0112	4	
Phylloscopus collybita	Common Chiffchaff	0.8187	131	✓
Sylvia atricapilla	Eurasian Blackcap	0.9637	134	✓
Sylvia borin	Garden Warbler	0.0028	1	
Sylvia curruca	Lesser Whitethroat	0.0056	4	
Regulus ignicapilla	Common Firecrest	0.5146	130	✓
Regulus regulus	Goldcrest	0.8731	133	✓
Troglodytes troglodytes	Eurasian Wren	0.7838	131	✓
Sitta europaea	Eurasian Nuthatch	0.3696	111	✓

Certhia brachydactyla	Short-toed Treecreeper	0.1088	49	✓
Certhia familiaris	Eurasian Treecreeper	0.5537	132	✓
Sturnus vulgaris	Common Starling	0.0153	6	
Turdus philomelos	Song Thrush	0.4700	124	✓
Turdus viscivorus	Mistle Thrush	0.3222	104	✓
Turdus iliacus	Redwing	0.0056	2	
Turdus merula	Eurasian Blackbird	0.9972	134	✓
Turdus pilaris	Fieldfare	0.1116	12	✓
Turdus torquatus	Ring Ouzel	0.0056	3	
Muscicapa striata	Spotted Flycatcher	0.0125	7	
Erithacus rubecula	European Robin	1.2971	135	✓
Ficedula hypoleuca	European Pied Flycatcher	0.0014	1	
Phoenicurus phoenicurus	Common Redstart	0.0126	4	
Prunella modularis	Dunnock	0.3501	98	✓
Motacilla cinerea	Grey Wagtail	0.0042	3	
Motacilla alba	White Wagtail	0.0084	6	
Anthus pratensis	Meadow Pipit	0.0014	1	
Anthus trivialis	Tree Pipit	0.0042	2	
Anthus spinoletta	Water Pipit	0.0014	1	
Fringilla coelebs	Eurasian Chaffinch	1.8354	135	✓
Fringilla montifringilla	Brambling	0.0795	5	
Coccothraustes coccothraustes	Hawfinch	0.0349	17	✓
Pyrrhula pyrrhula	Eurasian Bullfinch	0.2106	86	✓
Chloris chloris	European Greenfinch	0.0321	13	✓
Loxia curvirostra	Common Crossbill	0.2092	59	✓
Carduelis carduelis	European Goldfinch	0.0139	8	
Carduelis citrinella	Citril Finch	0.0042	1	
Serinus serinus	European Serin	0.0042	3	
Spinus spinus	Eurasian Siskin	0.3138	73	✓
Emberiza citrinella	Yellowhammer	0.0014	1	

References

Gill, F., Donsker, D. & Rasmussen, P. (Eds.) (2023). *IOC World Bird List* (version 13.2). https://doi.org/10.14344/IOC.ML.13.2.
