# Supplementary material for: Disentangling the effects of multifunctional forestry practices on the abundances of birds and their invertebrate prey
Source: Ecol Appl. 2026 Mar 8;36(2):e70198. doi: 10.1002/eap.70198 (PMC12967705; doi:10.1002/eap.70198)
Supplement: Supplementary file 4 — Appendix S4. [file EAP-36-e70198-s005.pdf]

Appendix S4

Summary of bird abundance models

Journal: Ecological Applications

Title: Disentangling the effects of multifunctional forestry practices on the abundances of birds and their invertebrate prey

Authors: João Manuel Cordeiro Pereira, Sara Klingenfuß, Marco Basile, Julian Frey, Grzegorz Mikusiński, Ilse Storch

Table S1: Details of *N*-mixture models for the abundance of 30 focal bird species, including: which random effects were included, which distribution was assumed for abundance (Poisson or negative binomial), how many variables were included (in the abundance sub-model, followed by detection sub-model), the apparent detection probability (i.e. the average ratio of yearly counts of the species in each plot to the estimated abundance), the average expected abundance across all plots and years (λ) and the posterior predictive probability (i.e. the probability that the discrepancy of simulated vs. fitted abundances is higher than the discrepancy of observed vs. fitted abundances). Values in brackets are those of a null model, containing no fixed effects for abundance, that was used to generate abundance estimates for use in the SEMs. For each species, the foraging guild (**G**: ground-foraging invertivore; **F**: foliage-gleaning invertivore; **B**: bark-foraging invertivore; **H**: herbivore/granivore) and the nesting guild are also indicated. Values of Average λ with an asterisk denote unrealistic estimates of abundance (reaching > 20 individuals in at least one plot), possibly due to flocking behaviour.

Species	Forag. guild	Nesting guild	Random effects	Distributio n	No. variables	Apparent *p*	Average λ	Posterior predictive p
Black Woodpecker (*Dryocopus martius*)	**G**	Cavity	Observer	Poisson	12 + 2	11.2 % (10.4 %)	0.762 (0.855)	0.473 (0.516)
Eurasian Wren (*Troglodytes troglodytes*)	**G**	Ground	Observer	Poisson	10 + 2	40.6 % (48.5 %)	1.926 (1.615)	1.000 (1.000)
Song Thrush (*Turdus philomelos*)	**G**	Canopy	Observer	Poisson	13 + 2	11.8 % (13.2 %)	4.002 (3.561)	0.675 (0.695)
Mistle Thrush (*Turdus viscivorus*)	**G**	Canopy	Observer	Poisson	13 + 2	16.1 % (18.5 %)	2.029 (1.737)	0.175 (0.144)
Eurasian Blackbird (*Turdus merula*)	**G**	Shrubs	Observer	Poisson	10 + 2	24.8 % (26.7 %)	4.046 (3.732)	0.999 (1.000)

European Robin (*Erithacus rubecula*)	**G**	Shrubs	Observer	Poisson	10 + 2	46.3 % (47.3 %)	2.806 (2.744)	1.000 (1.000)
Dunnock (*Prunella modularis*)	**G**	Shrubs	Observer	Poisson	10 + 2	14.3 % (23.6 %)	2.513 (1.483)	0.788 (0.742)
Great Spotted Woodpecker (*Dendrocopos major*)	**F**	Cavity	Observer, Year	Poisson	10 + 2	13.5 % (15.1 %)	3.440 (3.105)	0.220 (0.255)
Eurasian Jay (*Garrulus glandarius*)	**F**	Canopy	Observer	NB	11 + 2	4.4 % (3.3 %)	5.155* (6.936)	0.160 (0.233)
Coal Tit (*Periparus ater*)	**F**	Cavity	Observer, Year	Poisson	10 + 2	17.9 % (20.1 %)	6.456 (5.701)	1.000 (1.000)
European Crested Tit (*Lophophanes cristatus*)	**F**	Cavity	Observer	Poisson	10 + 2	13.3 % (18.8 %)	4.093 (2.861)	0.346 (0.550)
Marsh Tit (*Poecile palustris*)	**F**	Cavity	Observer	Poisson (NB)	11 + 2	9.4 % (6.2 %)	1.312 (0.848)	0.129 (0.435)
Eurasian Blue Tit (*Cyanistes caeruleus*)	**F**	Cavity	Year	Poisson (NB)	11 + 2	12.0 % (5.9 %)	1.622 (1.424)	0.100 (0.486)
Great Tit (*Parus major*)	**F**	Cavity	Observer	NB	13 + 2	13.3 % (13.8 %)	3.367 (3.315)	0.317 (0.277)
Long-tailed Tit (*Aegithalos caudatus*)	**F**	Shrubs	Observer	Poisson	9 + 2	17.8 % (17.9 %)	0.459 (0.471)	0.173 (0.081)
Common Chiffchaff (*Phylloscopus collybita*)	**F**	Ground	Observer	Poisson	13 + 2	26.2 % (40.9 %)	3.209 (2.003)	1.000 (0.996)
Eurasian Blackcap (*Sylvia atricapilla*)	**F**	Shrubs	Observer	Poisson	9 + 2	47.6 % (51.2 %)	2.047 (1.882)	0.974 (0.888)
Common Firecrest (*Regulus ignicapilla*)	**F**	Canopy	Observer	Poisson	8 + 2	28.2 % (30.0 %)	1.820 (1.713)	0.942 (0.953)
Goldcrest (*Regulus regulus*)	**F**	Canopy	Observer, Year	Poisson	8 + 2	24.1 % (34.5 %)	3.732 (2.555)	1.000 (1.000)
Eurasian Chaffinch (*Fringilla coelebs*)	**F**	Canopy	Observer	Poisson	11 + 2	51.1 % (53.7 %)	3.605 (3.418)	1.000 (1.000)
Hawfinch (*Coccothraustes coccothraustes*)	**F**	Canopy	Observer	Poisson	11 + 2	10.9 % (7.7 %)	0.329 (0.452)	0.214 (0.215)

Eurasian Nuthatch (*Sitta europaea*)	**B**	Cavity	Observer	Poisson	10 + 2	15.9 % (18.4 %)	2.353 (2.007)	0.562 (0.521)
Short-toed Treecreeper (*Certhia brachydactyla*)	**B**	Cavity	Observer	Poisson	10 + 2	8.2 % (11.2 %)	1.360 (0.969)	0.737 (0.507)
Eurasian Treecreeper (*Certhia familiaris*)	**B**	Cavity	Observer, Year	Poisson	10 + 2	16.0 % (16.2 %)	3.353 (3.317)	0.917 (0.951)
Stock Dove (*Columba oenas*)	**H**	Cavity	-	Poisson	7 + 2	15.1 % (33.4 %)	0.117 (0.117)	0.404 (0.290)
Common Wood Pigeon (*Columba palumbus*)	**H**	Canopy	Observer	NB	11 + 2	6.0 % (6.4 %)	6.464* (5.931)	0.425 (0.512)
Eurasian Bullfinch (*Pyrrhula pyrrhula*)	**H**	Shrubs	Observer, Year	Poisson	11 + 2	11.0 % (10.7 %)	1.952 (2.004)	0.195 (0.257)
European Greenfinch (*Chloris chloris*)	**H**	Shrubs	-	Poisson	11 + 2	9.8 % (12.0 %)	0.228 (0.268)	0.233 (0.144)
Common Crossbill (*Loxia curvirostra*)	**H**	Canopy	Observer, Year, Plot	NB	7 + 2	13.9 % (11.1 %)	2.417* (2.717)	0.676 (0.605)
Eurasian Siskin (*Spinus spinus*)	**H**	Canopy	Observer, Year	Poisson	8 + 2	13.7 % (12.8 %)	1.377 (1.409)	0.316 (0.393)
