# Supplementary material for: Disentangling the effects of multifunctional forestry practices on the abundances of birds and their invertebrate prey
Source: Ecol Appl. 2026 Mar 8;36(2):e70198. doi: 10.1002/eap.70198 (PMC12967705; doi:10.1002/eap.70198)

Appendix S5

Effect plots for all bird and invertebrate models

Journal: Ecological Applications

Title: Disentangling the effects of multifunctional forestry practices on the abundances of birds and their invertebrate prey

Authors: João Manuel Cordeiro Pereira, Sara Klingenfuß, Marco Basile, Julian Frey, Grzegorz Mikusiński, Ilse Storch

Figure S1: Posterior means and 90% credible intervals for fixed effect parameters in all bird *N*-mixture models (panels **a** to **dd**) and invertebrate abundance models (panels **ee** to **xx**; PT for pitfall traps and FIT for flight interception traps). Circles denote effect coefficients for forest predictors, and diamonds (only for birds) denote effect coefficients for ordinal date (*date*) and minutes since sunrise (*time*) on detection probability. Significant positive and negative effects (90% CI above and below 0, respectively) are colored blue and red, respectively. Legend for forest predictors: *mDBH*, mean tree diameter at breast height (DBH), *nsnag*, number of dead trees, *sndbh*, mean DBH of dead trees, *ldw*, volume of lying deadwood, *ENL***,** effective number of layers, *sdDBH*, standard deviation of tree DBH, *canopy*, canopy cover, *sCov***,** shrub layer cover, *hCov***,** herb layer cover, *broad***,** broadleaf share of basal area, *treSR***,** tree species richness, *undSR*, understory vascular plant richness, *alt***,** average elevation above sea level, *sdslo***,** standard deviation of terrain slope, *north***,** northness index.

(a) Black woodpecker (*Dryocopus martius*)

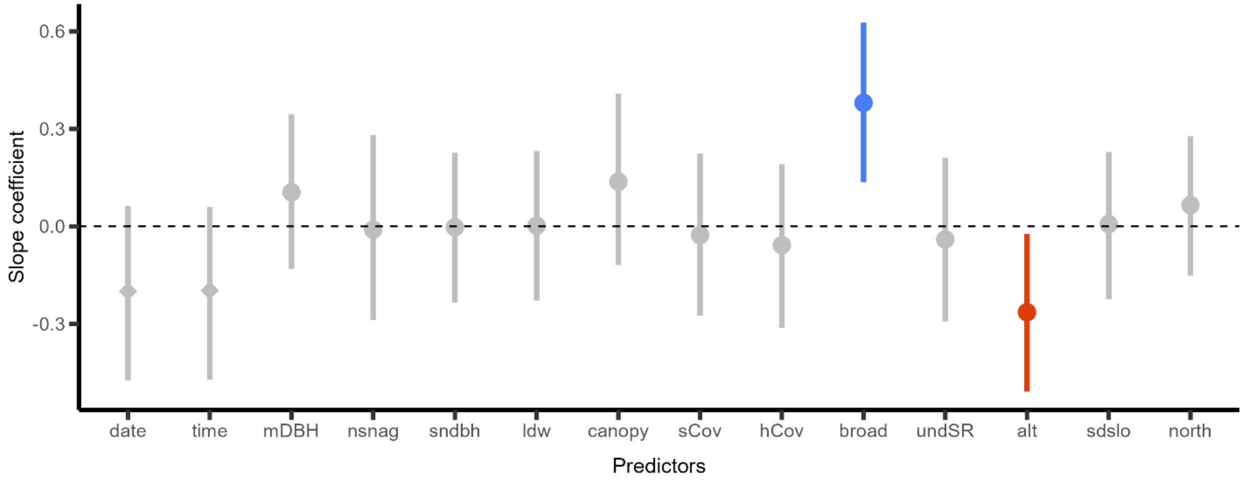

(b) Eurasian wren (*Troglodytes troglodytes*)

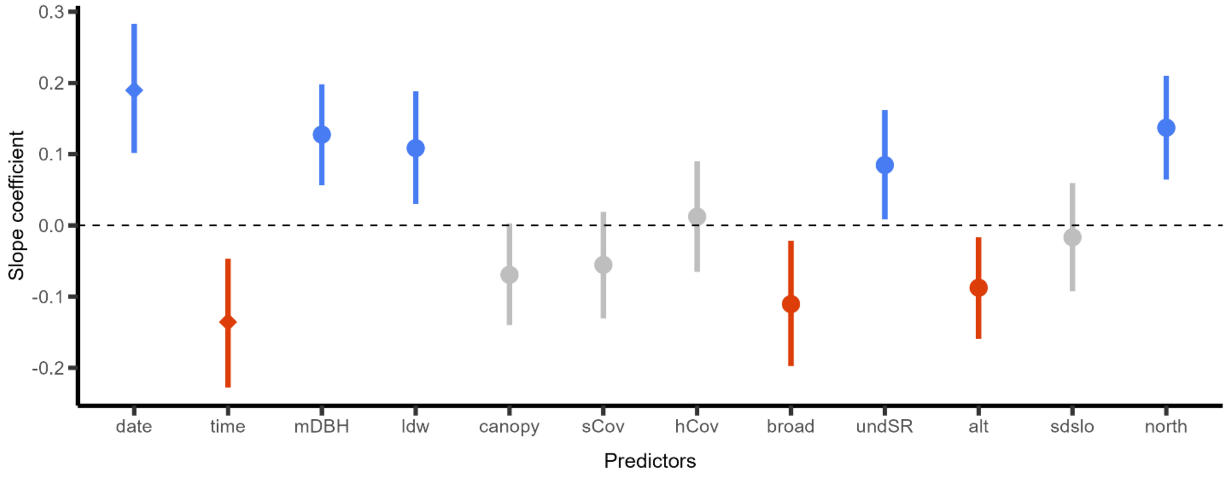

(c) Song thrush (*Turdus philomelos*)

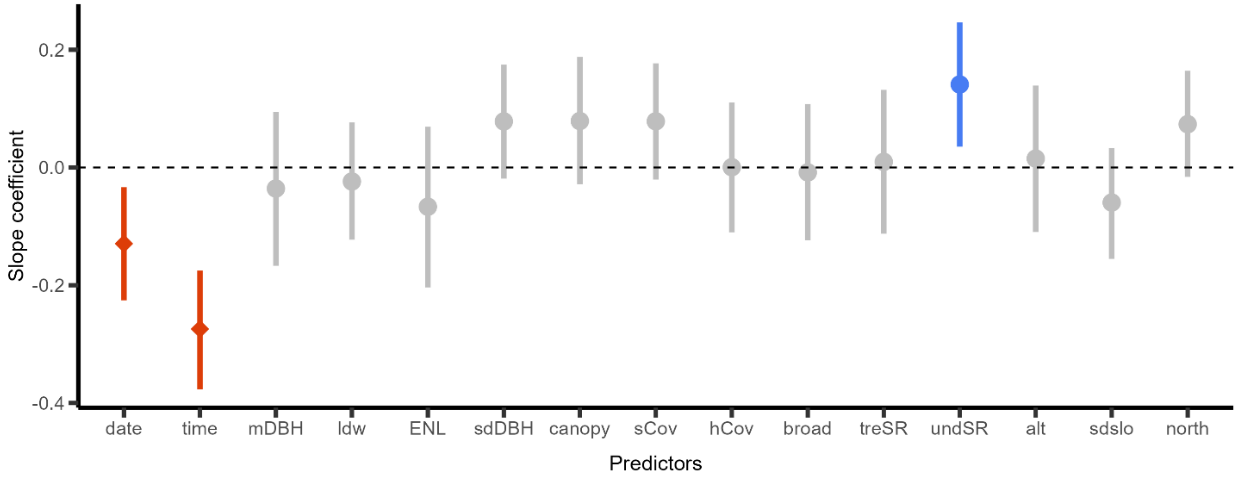

(d) Mistle thrush (*Turdus viscivorus*)

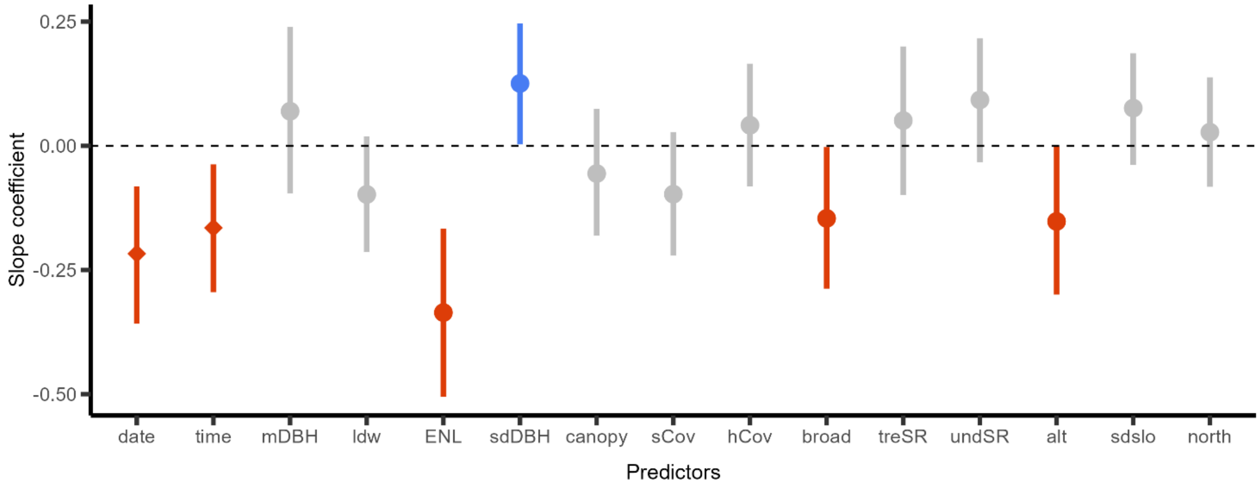

(e) Eurasian blackbird (*Turdus merula*)

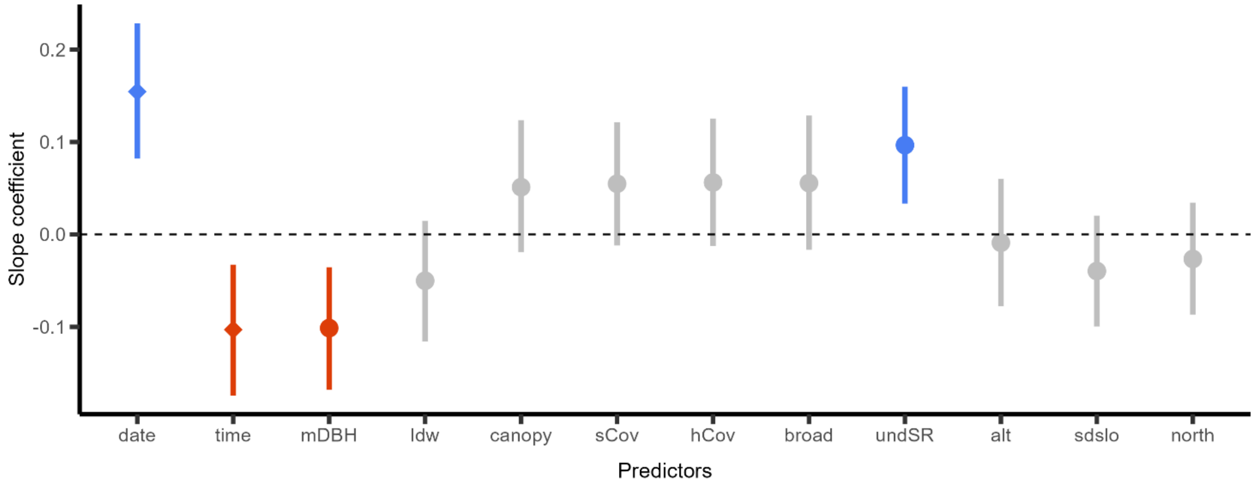

(f) European robin (*Erithacus rubecula*)

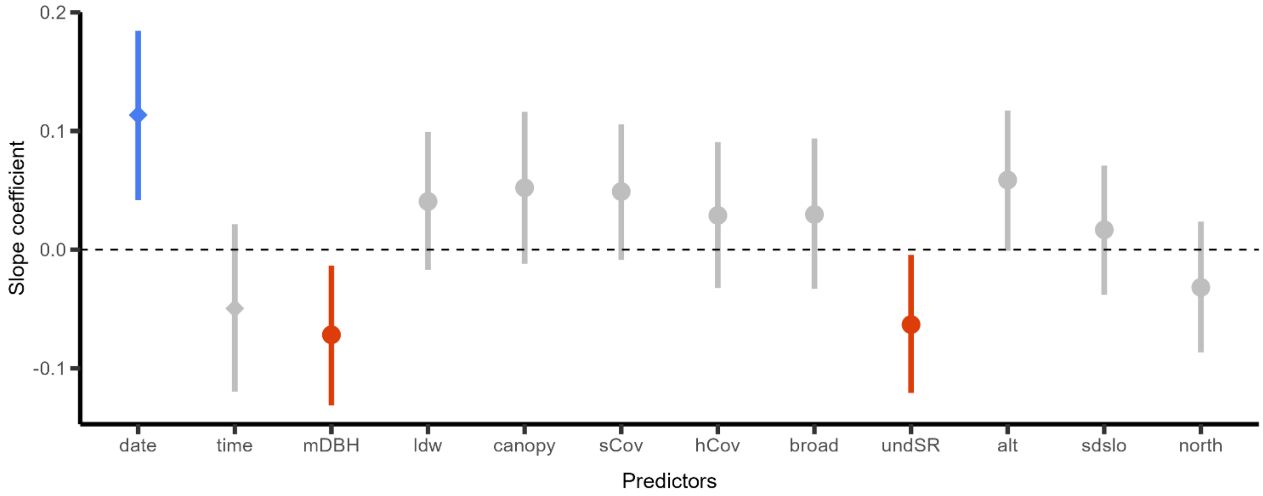

(g) Dunnock (*Prunella modularis*)

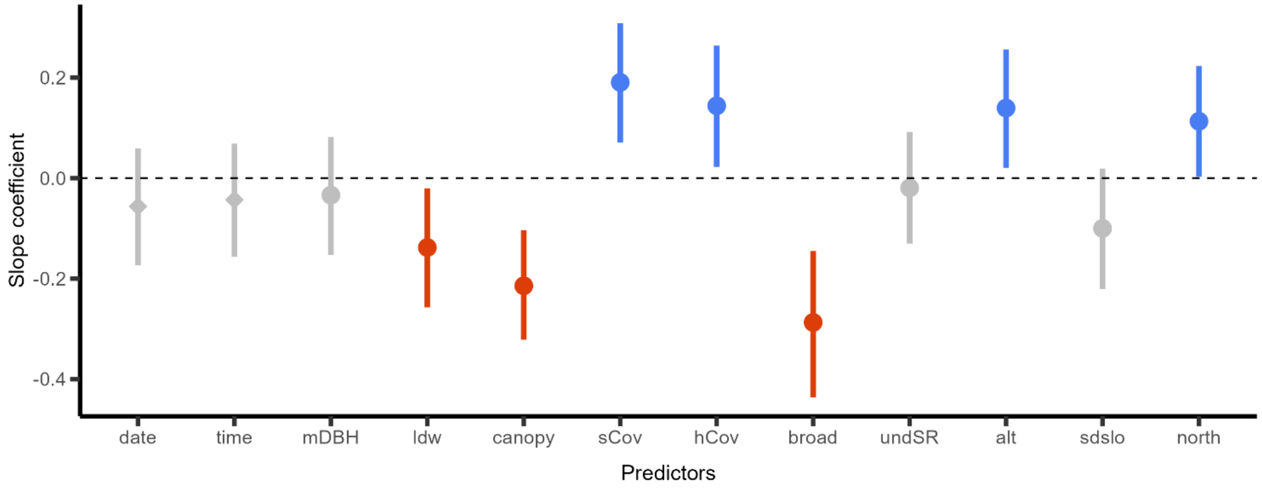

(h) Great spotted woodpecker (*Dendrocopos major*)

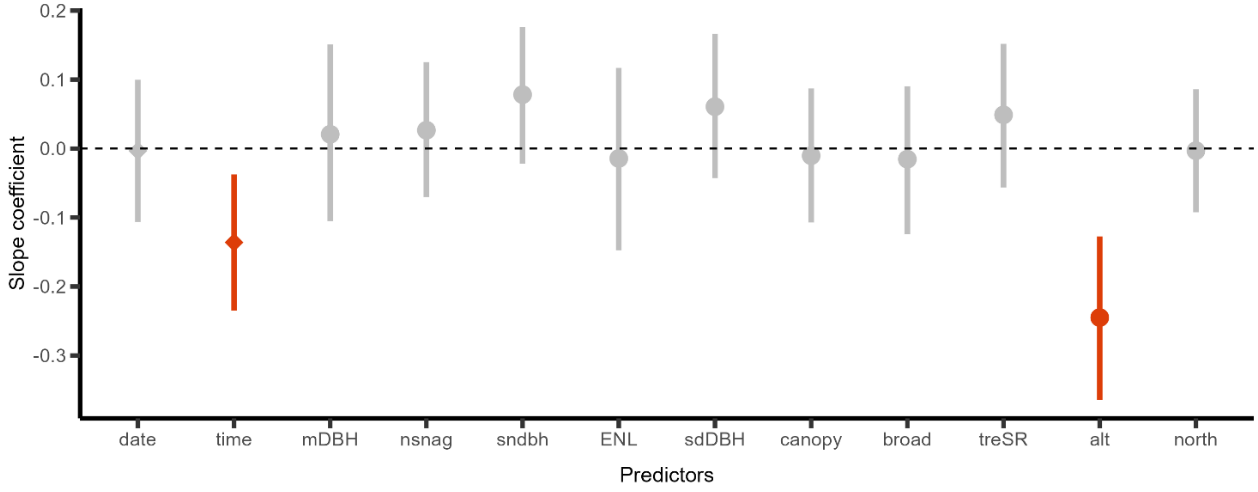

(i) Eurasian jay (*Garrulus glandarius*)

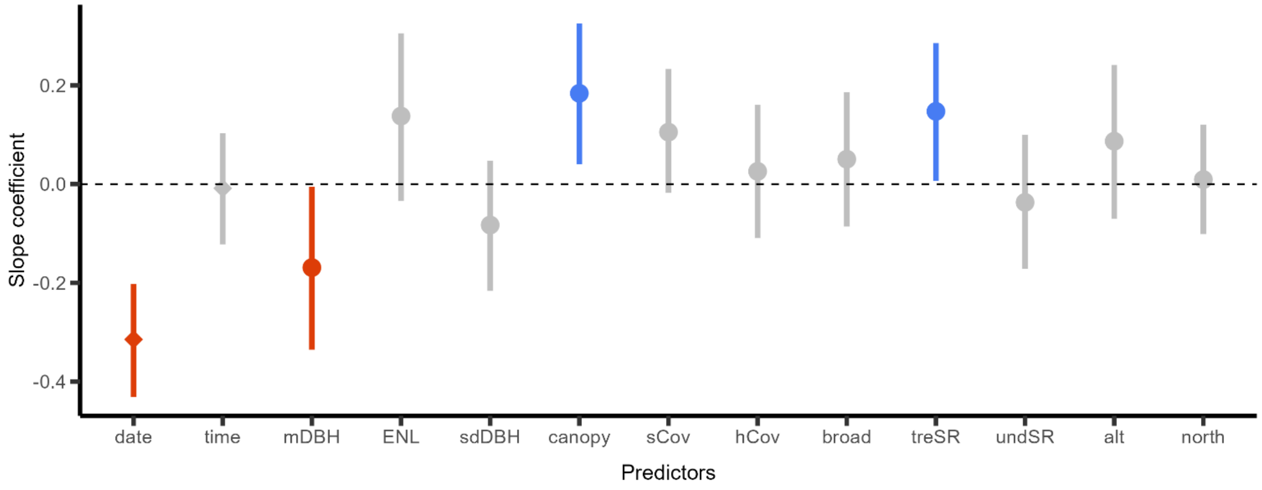

(j) Coal tit (*Periparus ater*)

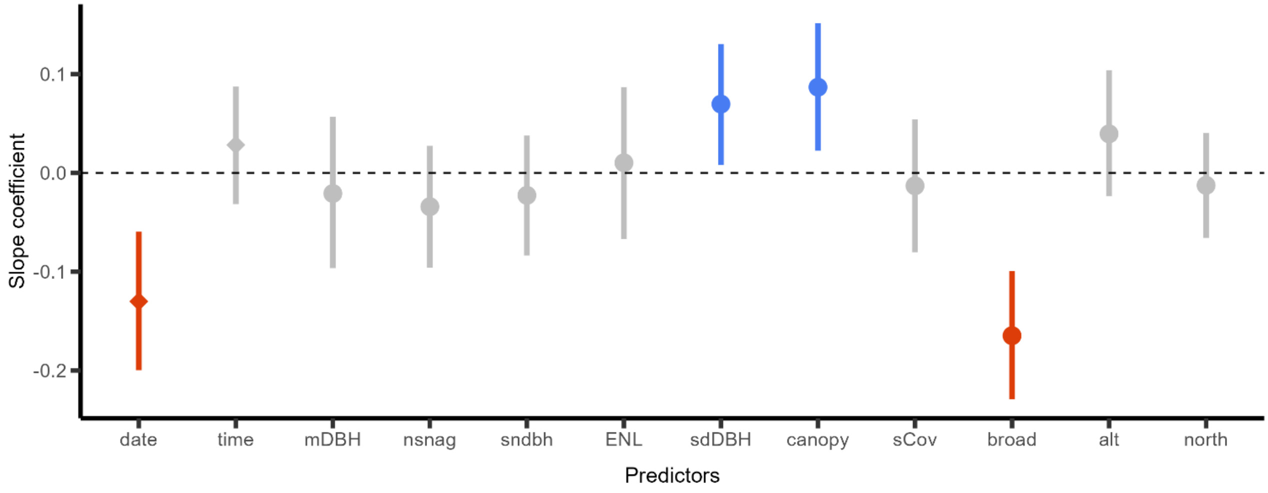

(k) European crested tit (*Lophophanes cristatus*)

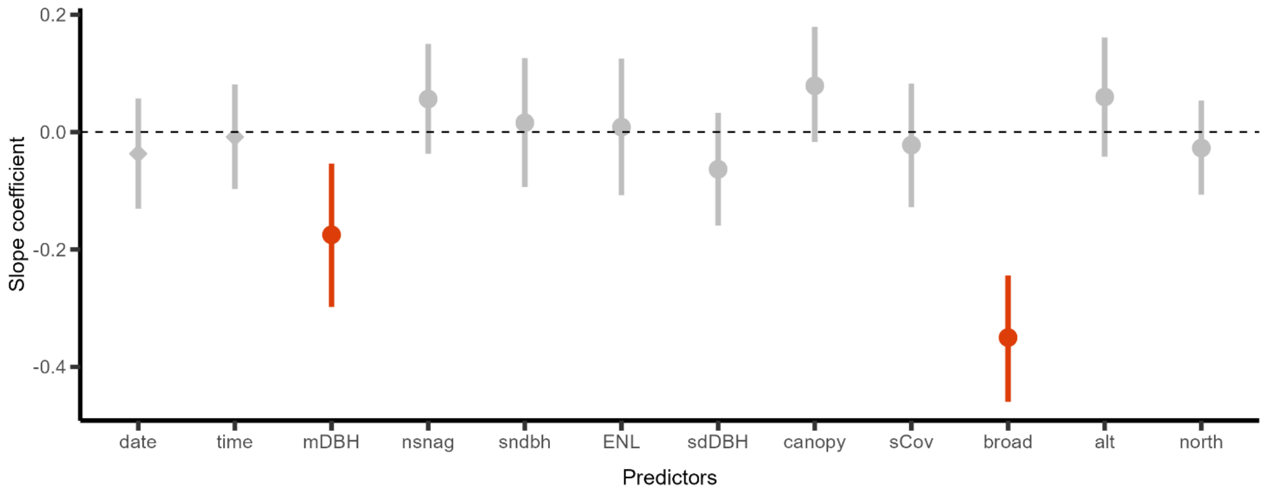

(l) Marsh tit (*Poecile palustris*)

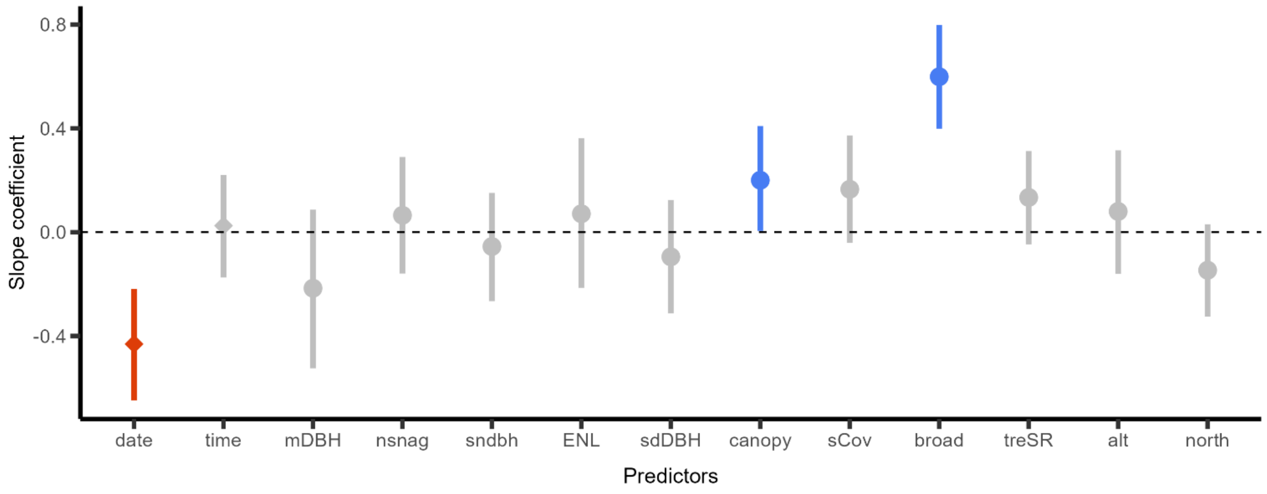

(m) Eurasian blue tit (*Cyanistes caeruleus*)

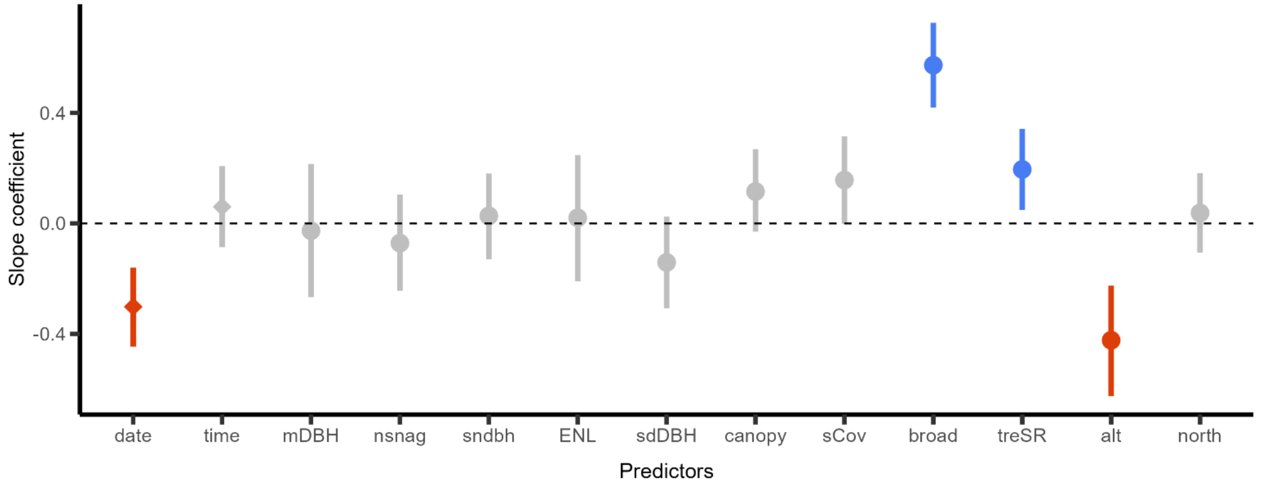

(n) Great tit (*Parus major*)

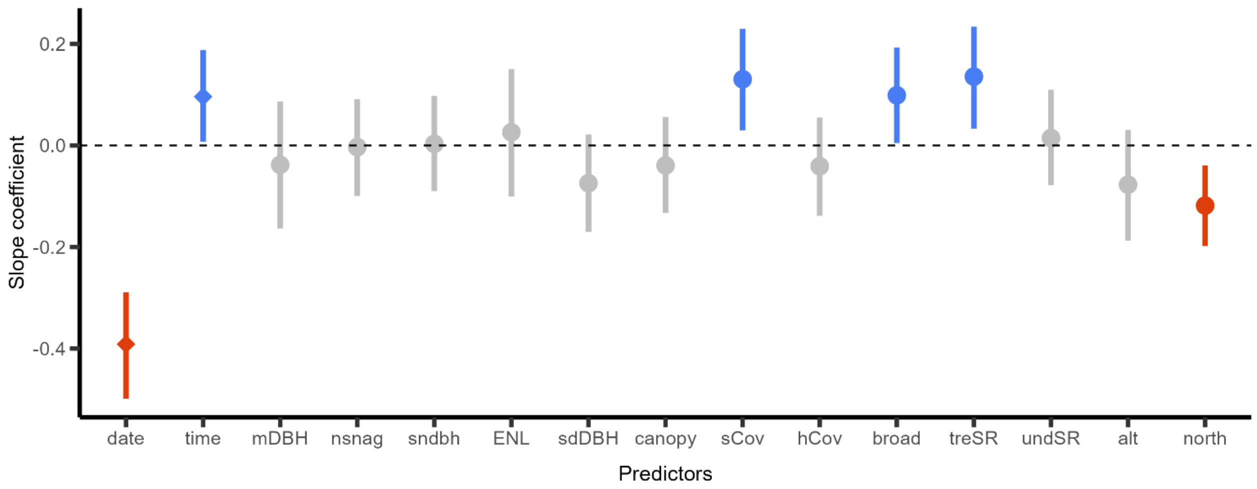

(o) Long-tailed tit (*Aegithalos caudatus*)

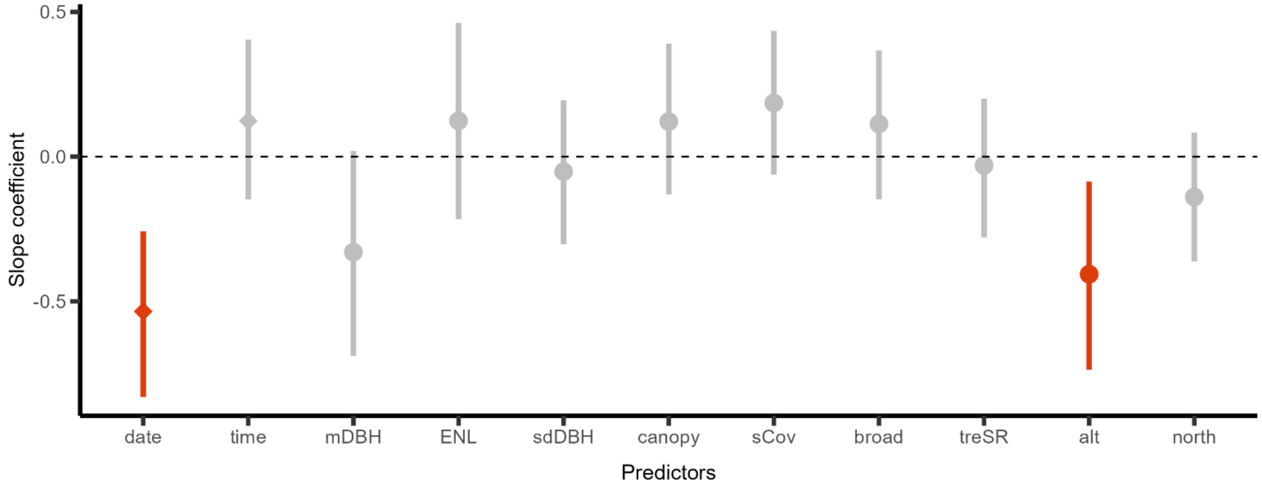

(p) Common chiffchaff (*Phylloscopus collybita*)

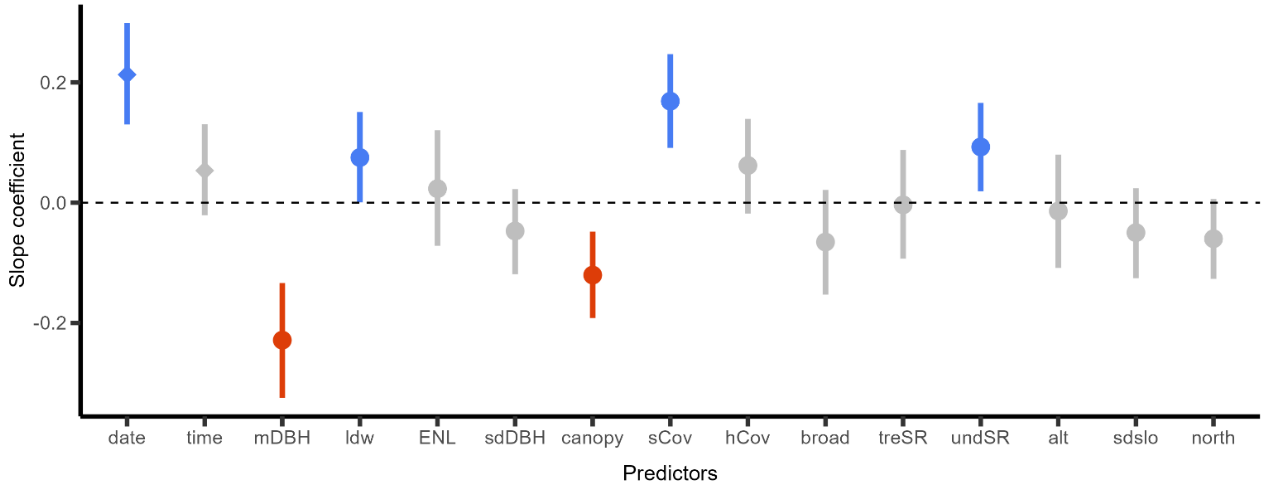

(q) Eurasian blackcap (*Sylvia atricapilla*)

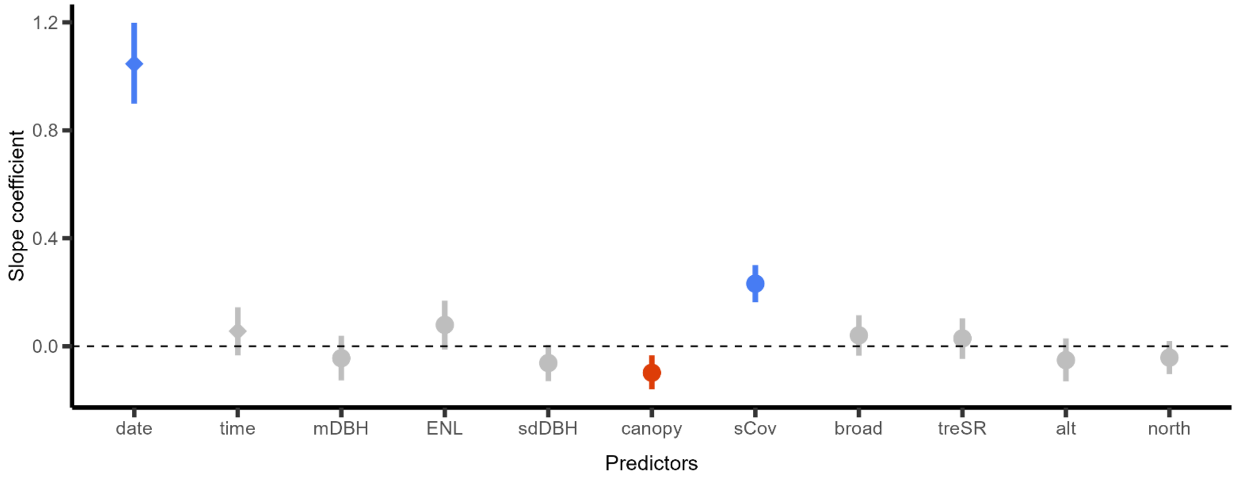

(r) Common firecrest (*Regulus ignicapilla*)

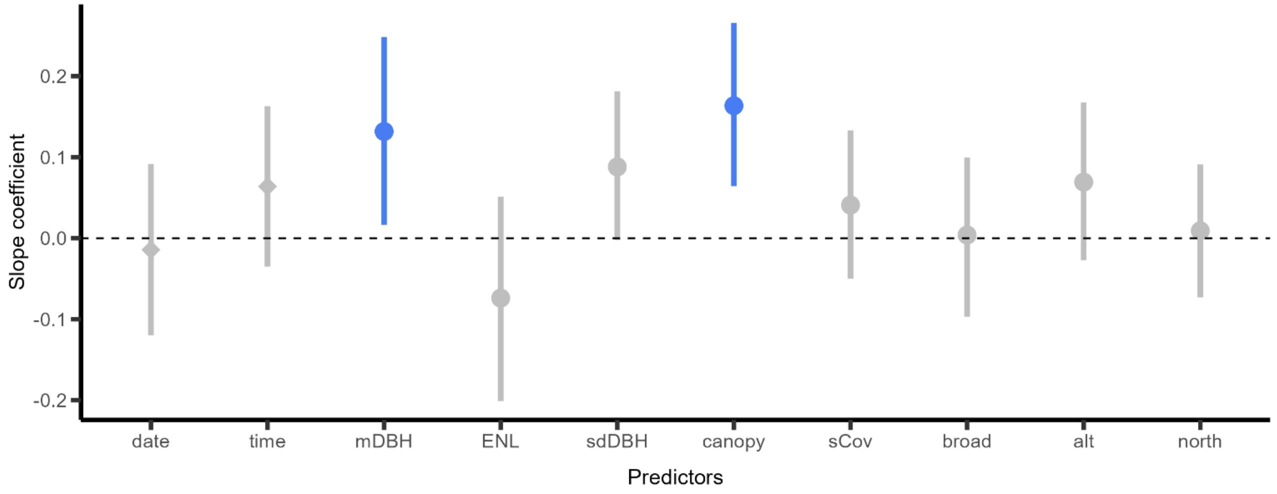

(s) Goldcrest (*Regulus regulus*)

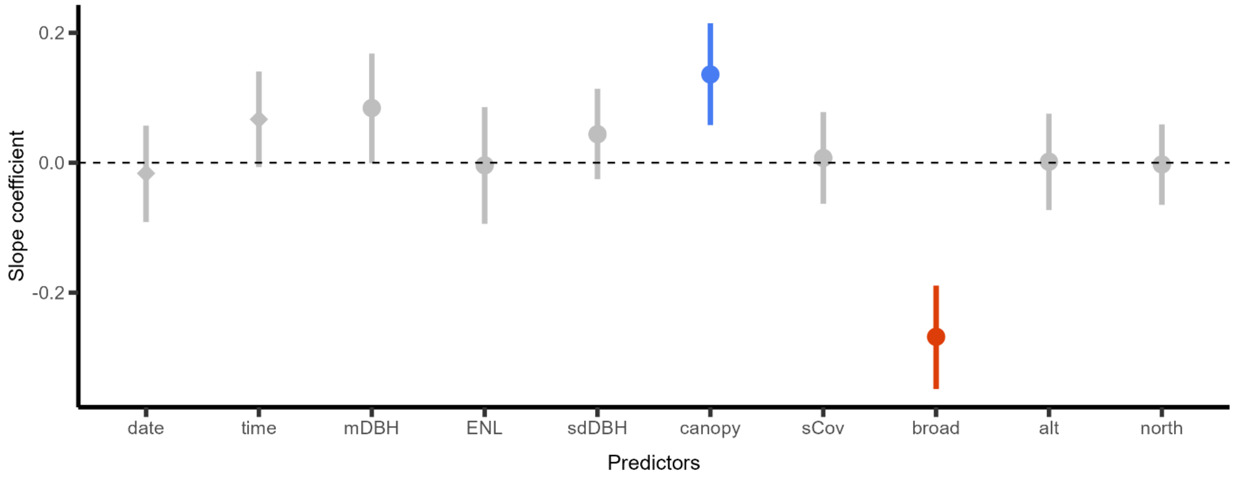

(t) Eurasian chaffinch (*Fringilla coelebs*)

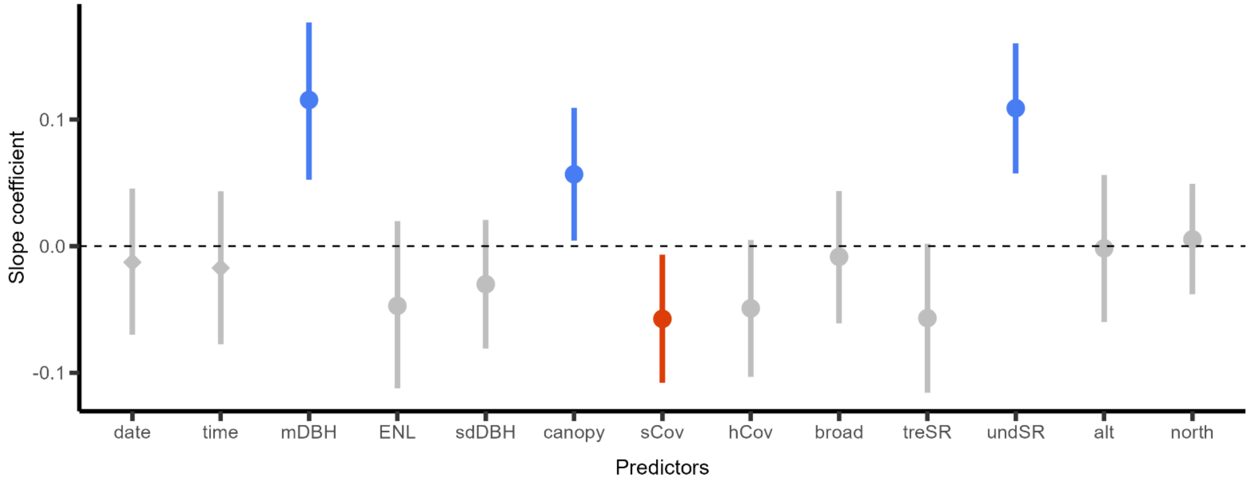

(u) Hawfinch (*Coccothraustes coccothraustes*)

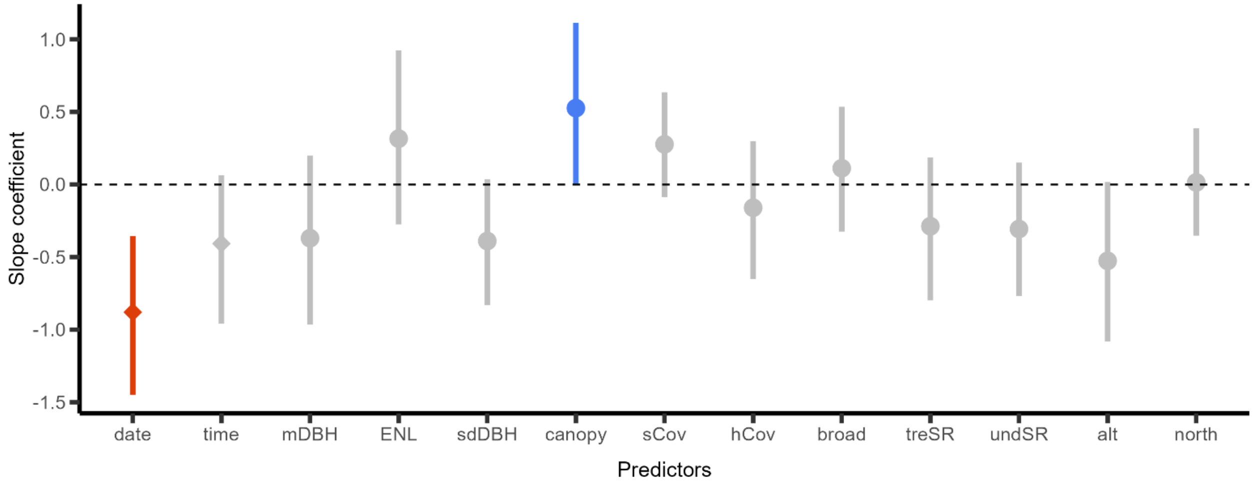

(v) Eurasian nuthatch (*Sitta europaea*)

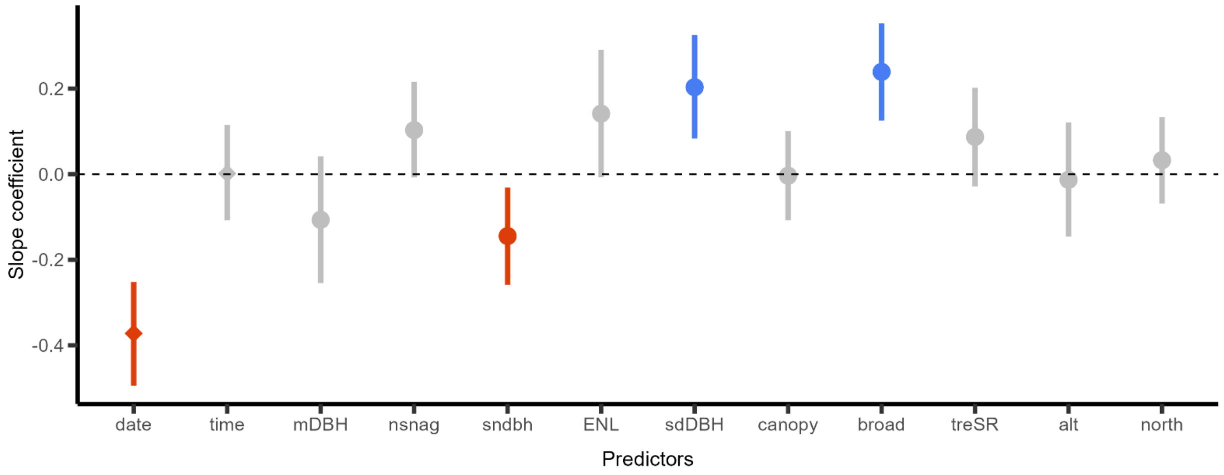

(w) Short-toed treecreeper (*Certhia brachydactyla*)

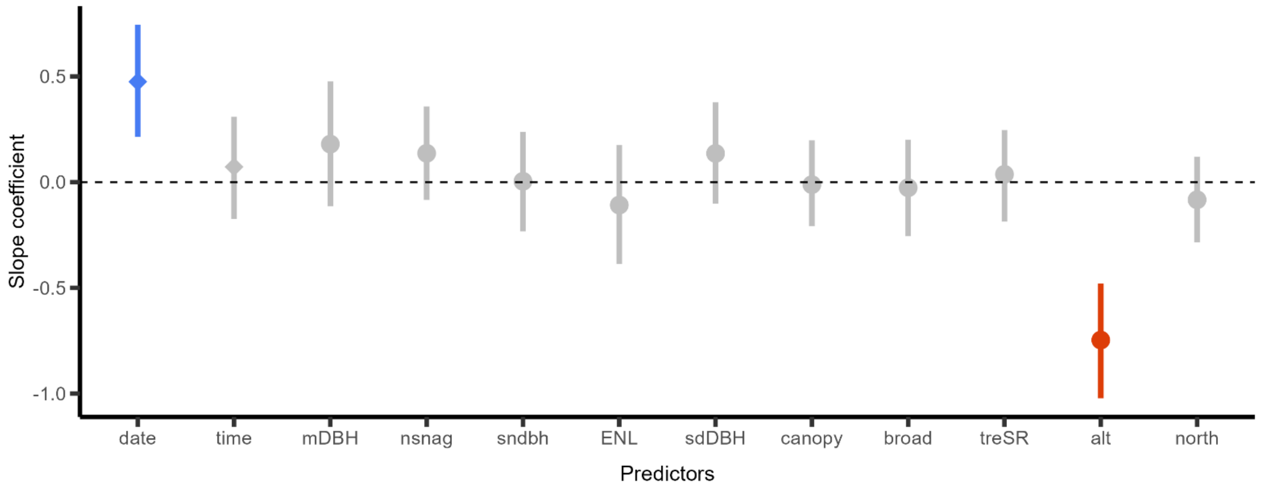

(x) Eurasian treecreeper (*Certhia familiaris*)

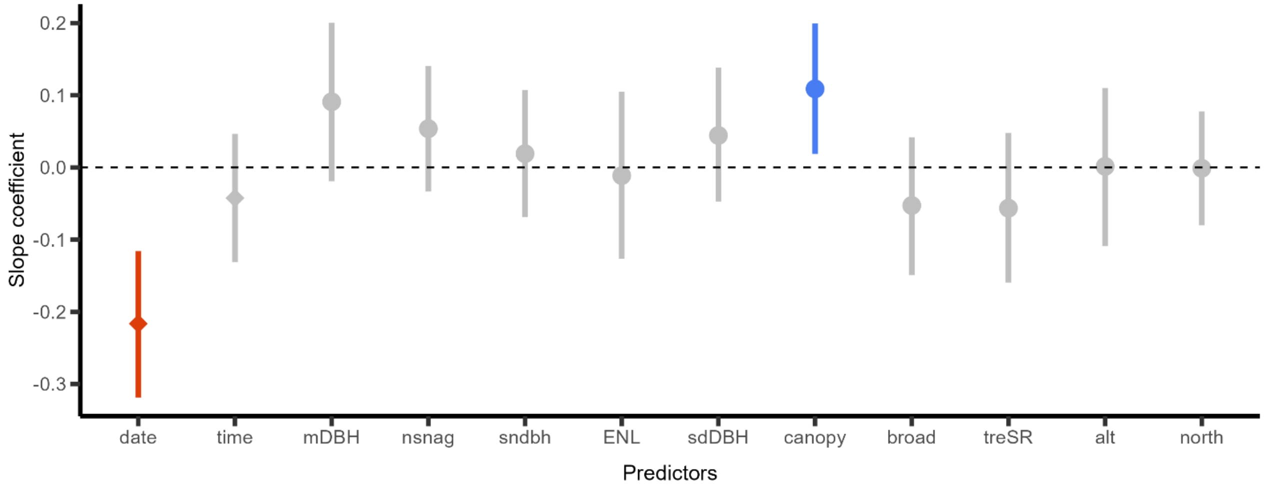

(y) Stock dove (*Columba oenas*)

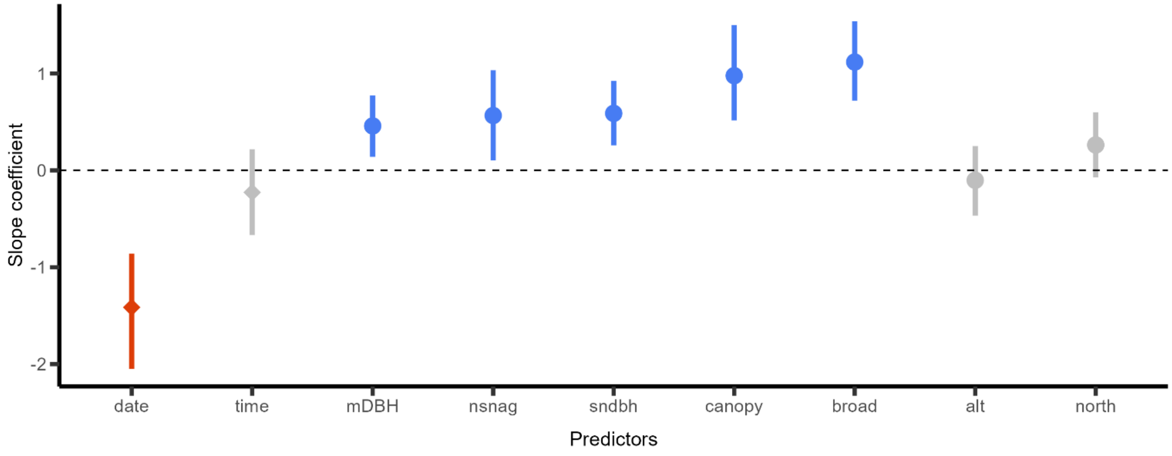

(z) Common wood pigeon (*Columba palumbus*)

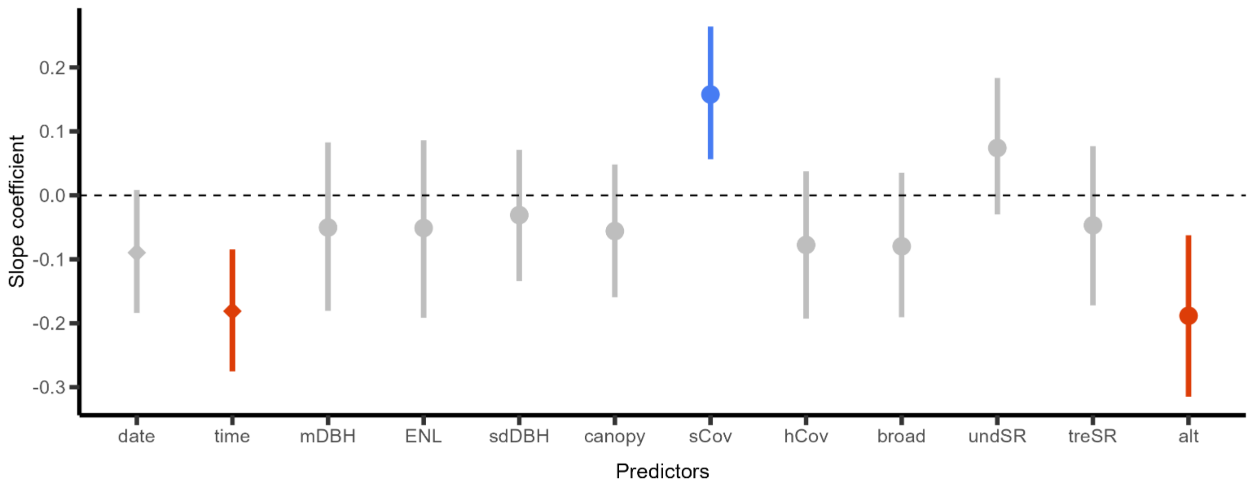

(aa) Eurasian bullfinch (*Pyrrhula pyrrhula*)

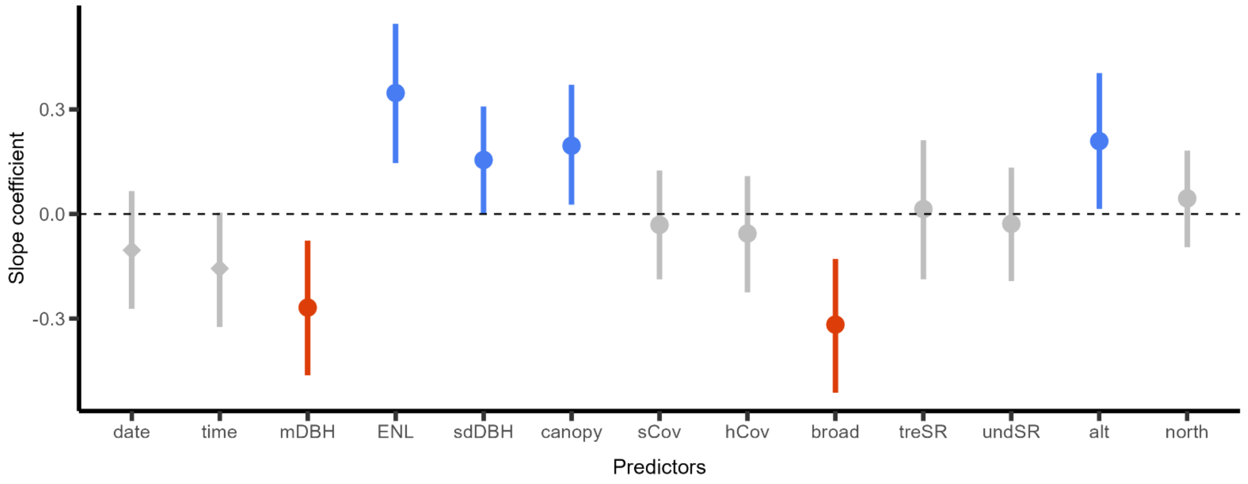

(bb) European greenfinch (*Chloris chloris*)

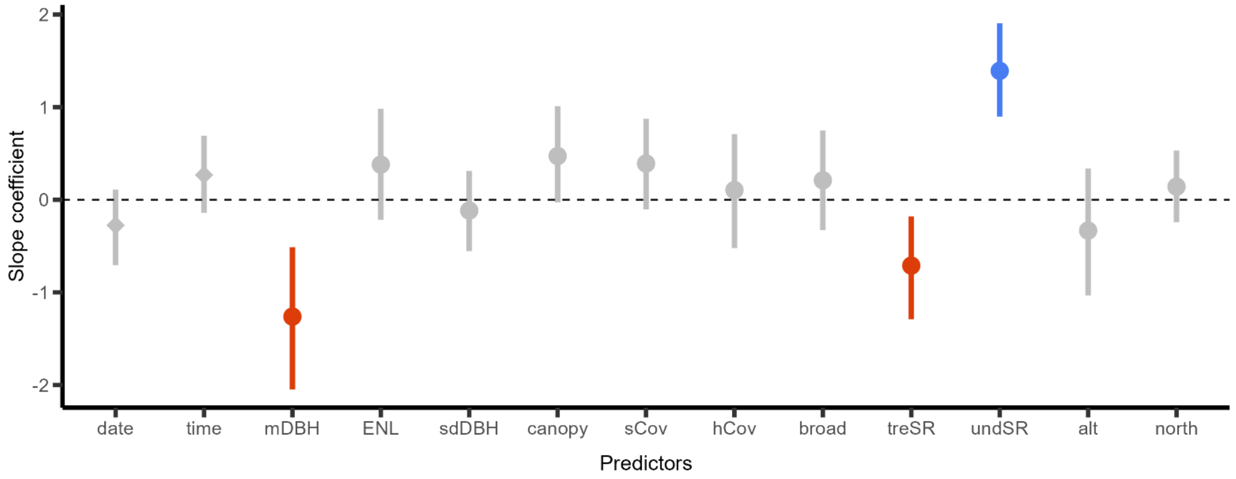

(cc) Common crossbill (*Loxia curvirostra*)

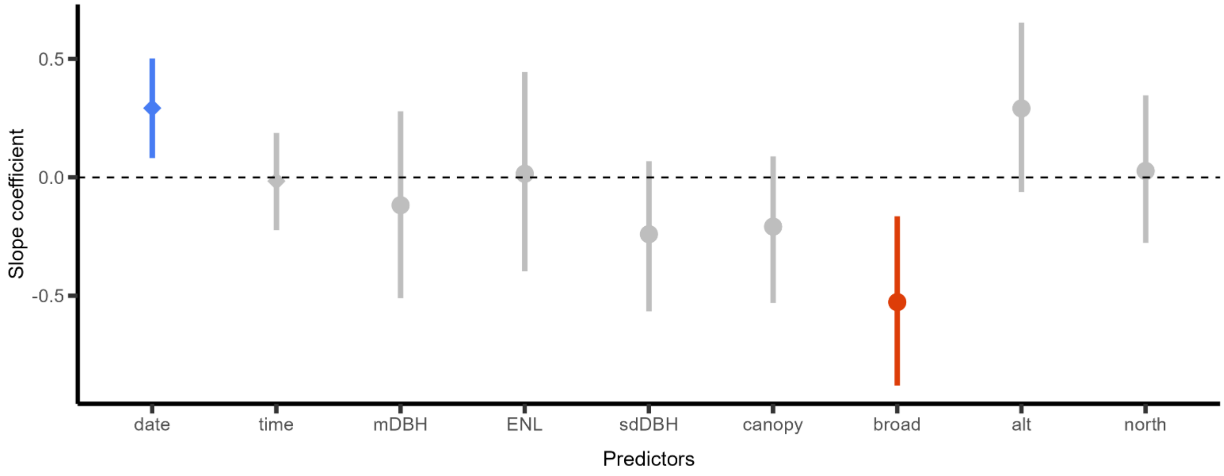

(dd) Eurasian siskin (*Spinus spinus*)

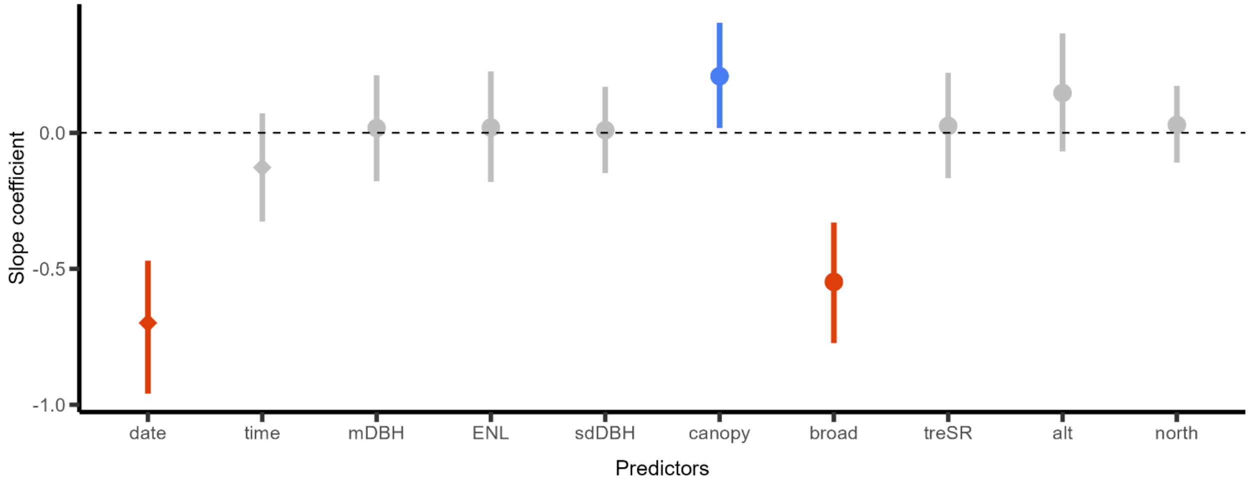

(ee) Gastropoda (PT)

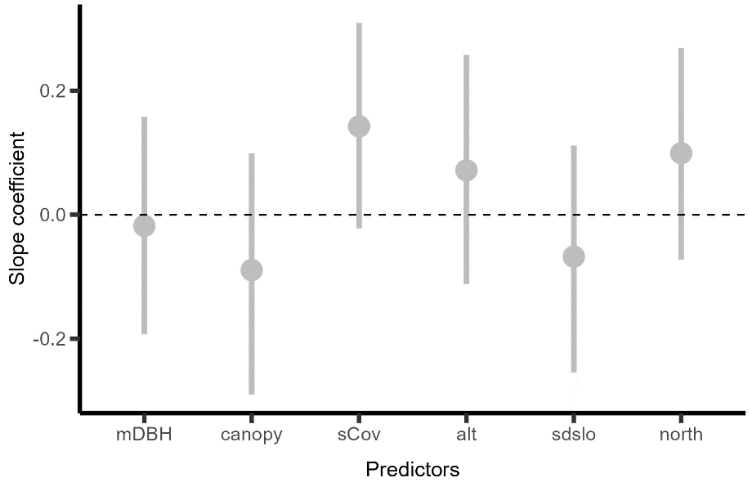

(ff) Opiliones (PT)

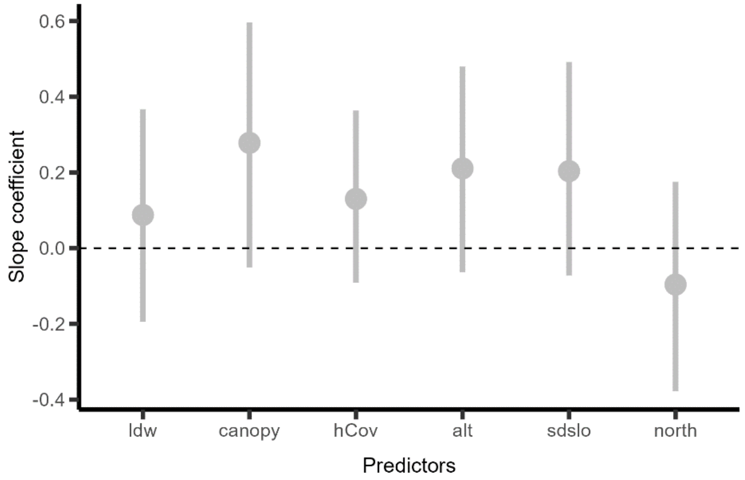

(gg) Araneae (PT)

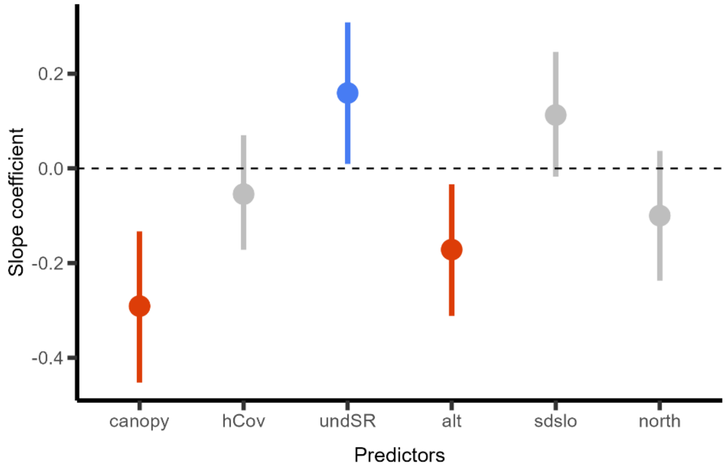

(hh) Chilopoda (PT)

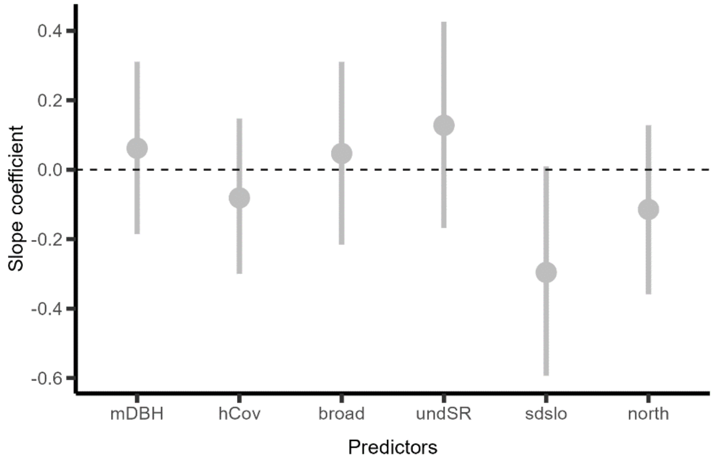

(ii) Diplopoda (PT)

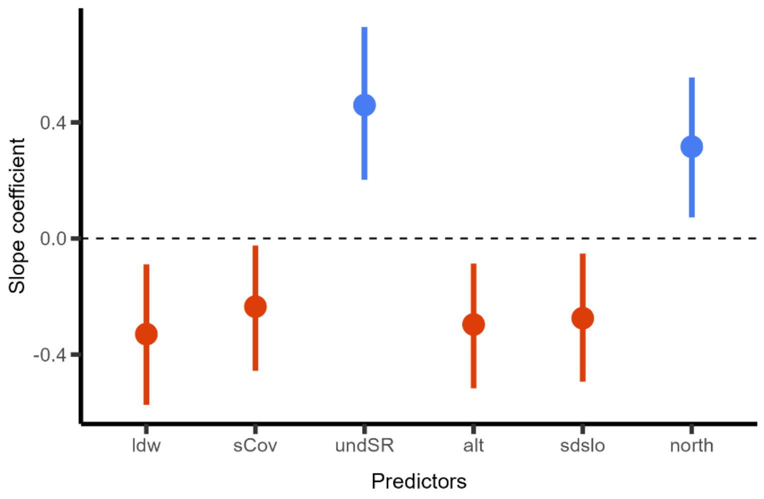

(jj) Isopoda (PT)

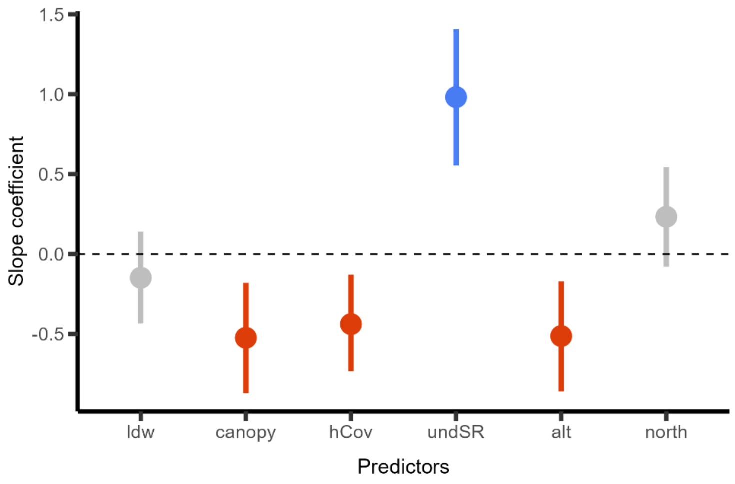

(kk) Collembola (PT)

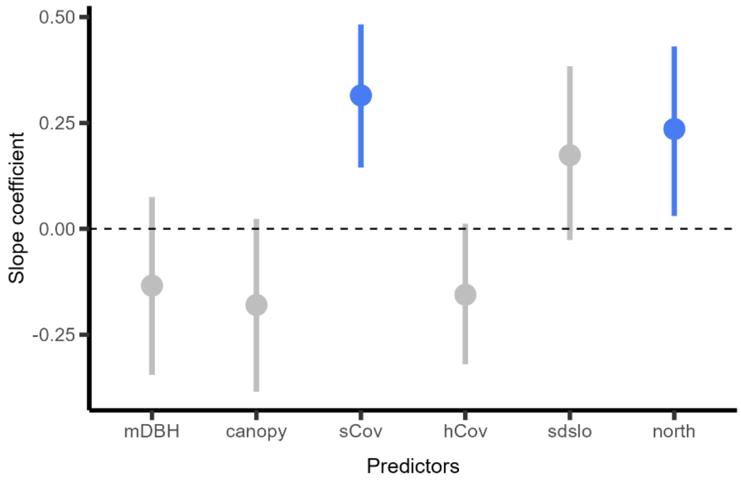

(ll) Hymenoptera (PT)

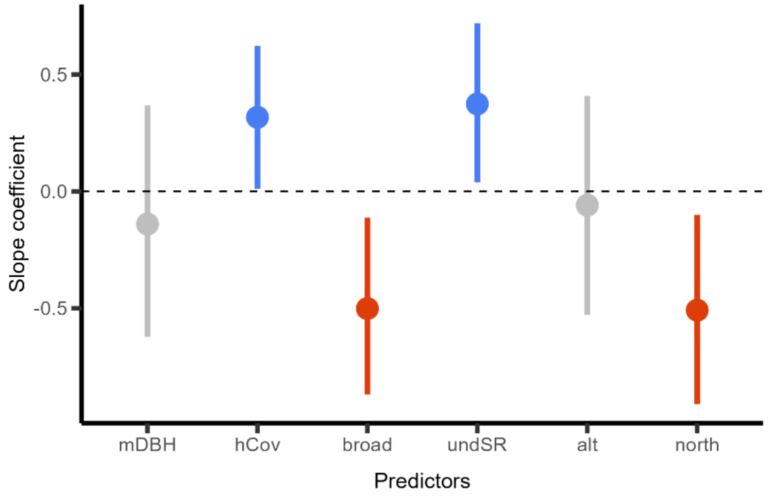

(mm) Diptera (PT)

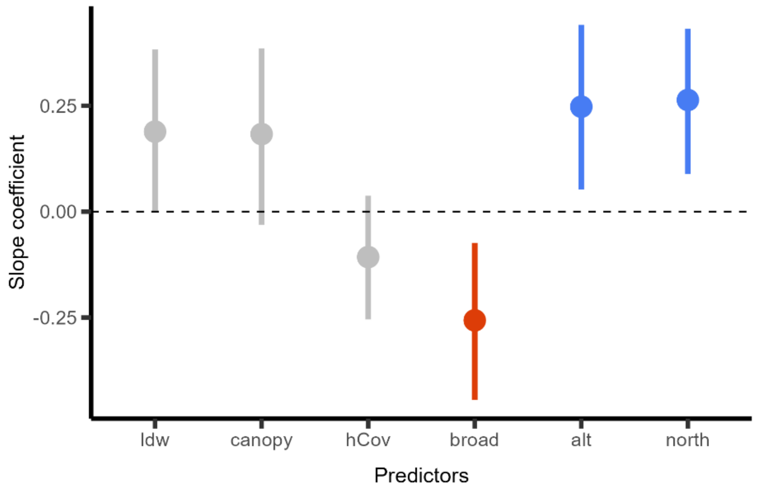

(nn) Coleoptera (PT)

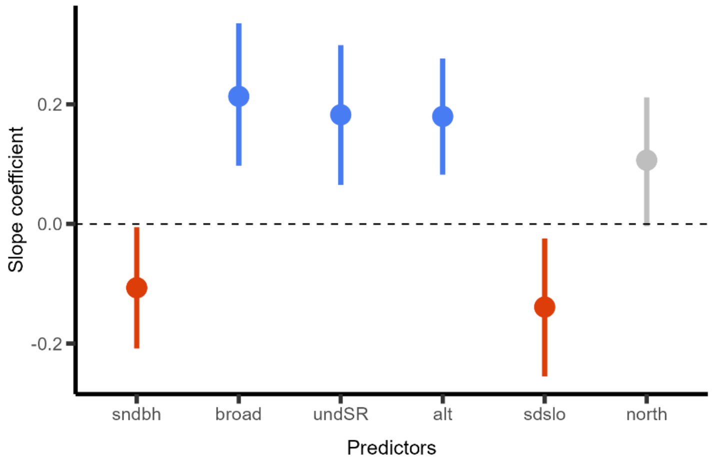

(oo) Araneae (FIT)

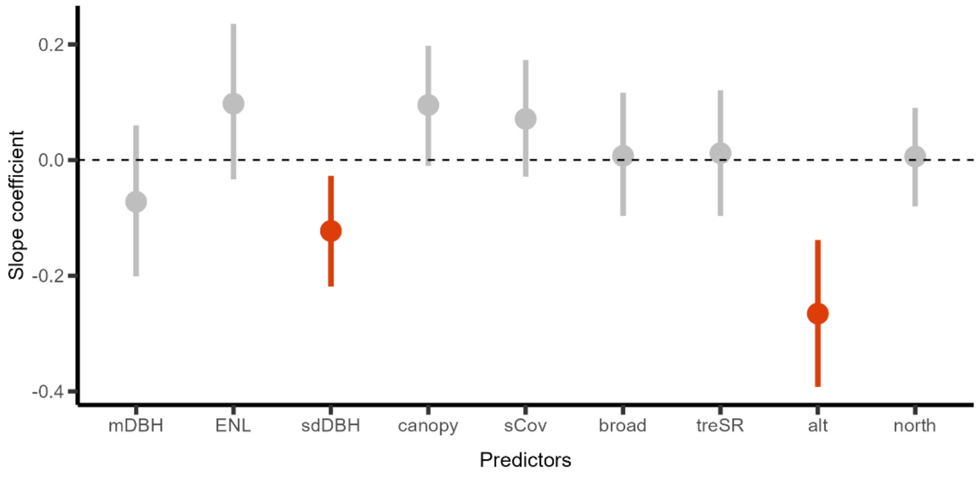

(pp) Collembola (FIT)

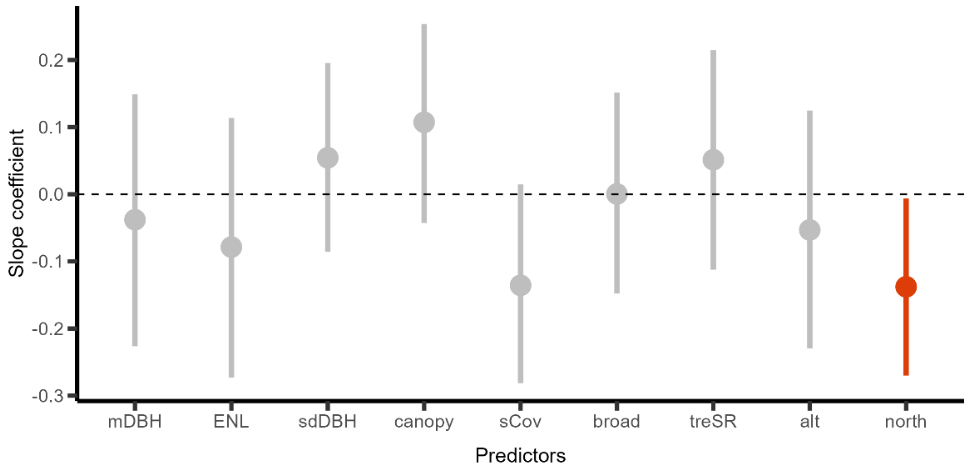

(qq) Psocoptera (FIT)

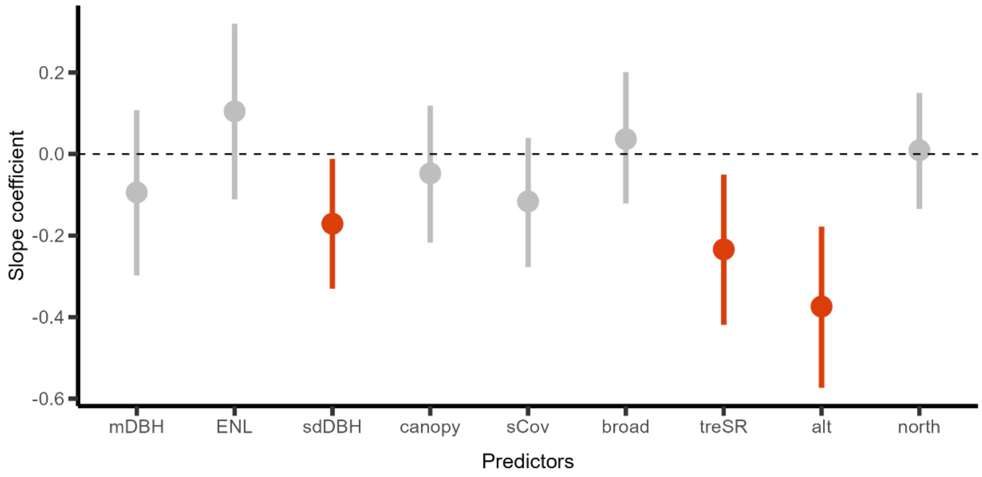

(rr) Auchenorrhyncha (FIT)

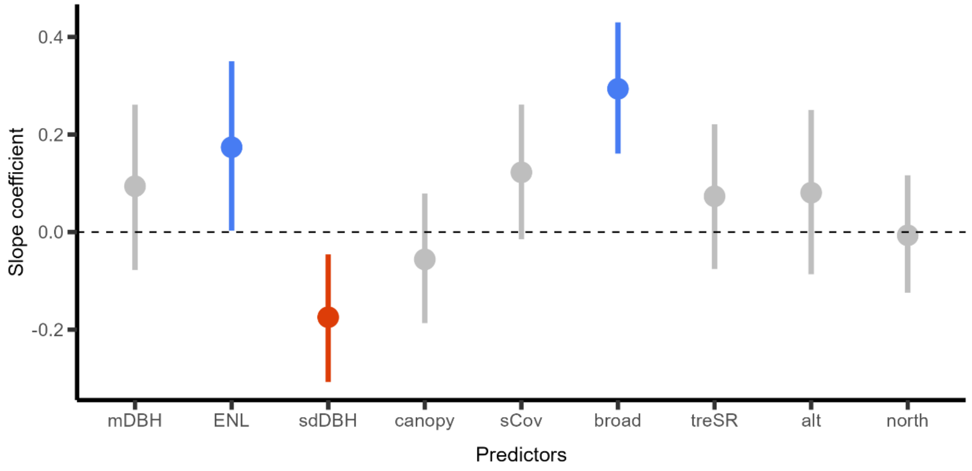

(ss) Sternorrhyncha (FIT)

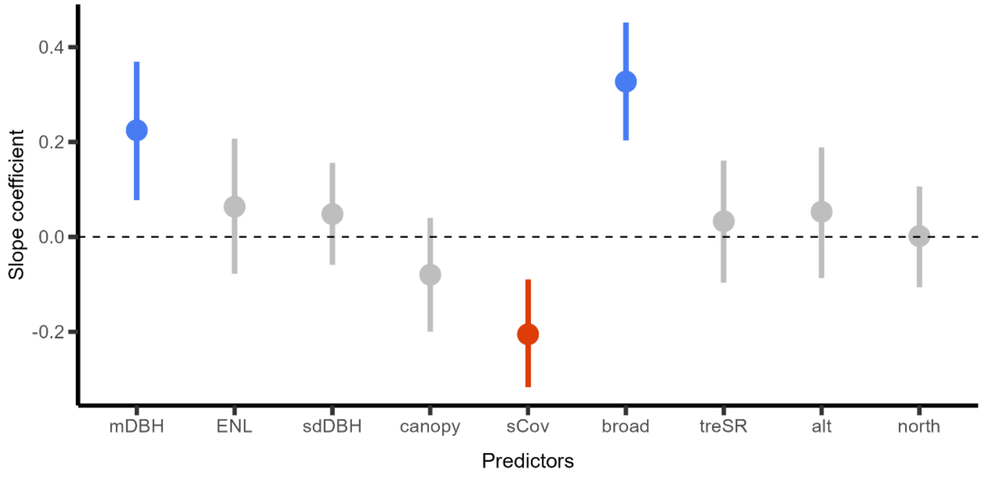

(tt) Heteroptera (FIT)

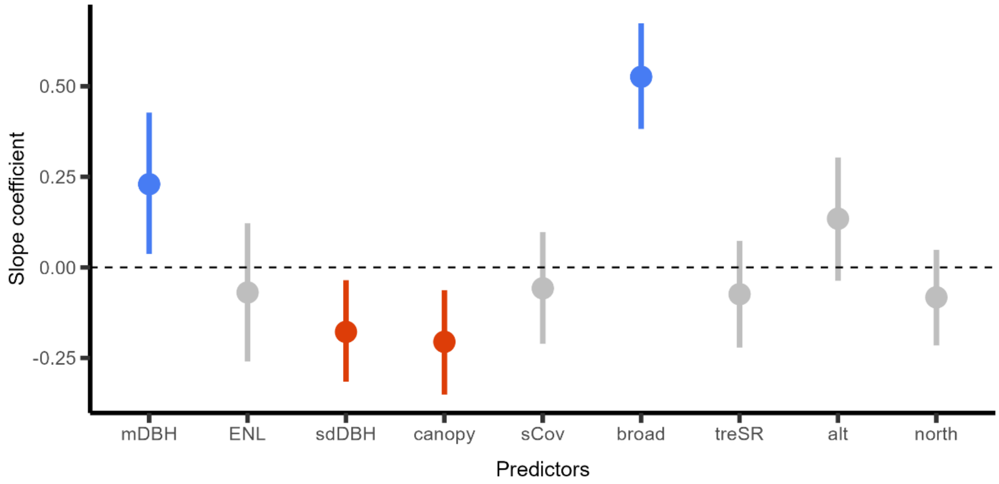

(uu) Lepidoptera (FIT)

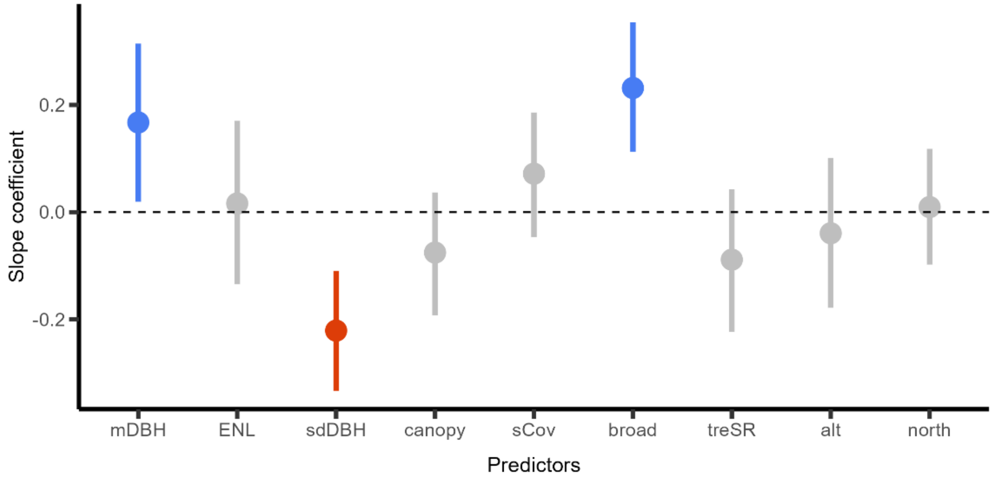

(vv) Diptera (FIT)

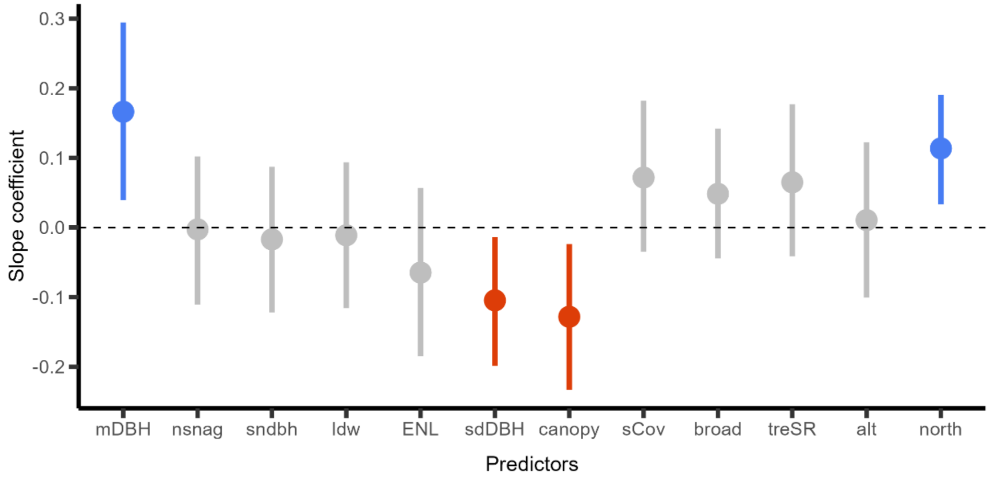

(ww) Hymenoptera (FIT)

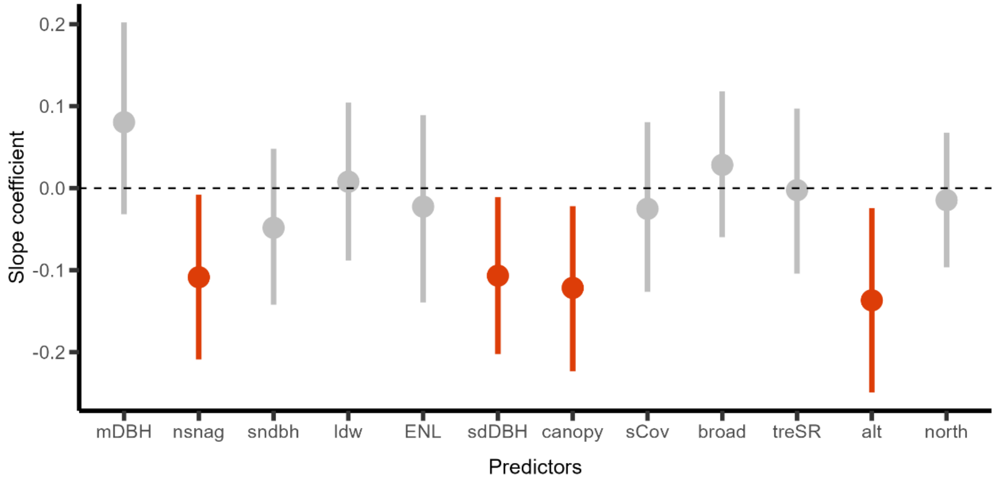

(xx) Coleoptera (FIT)

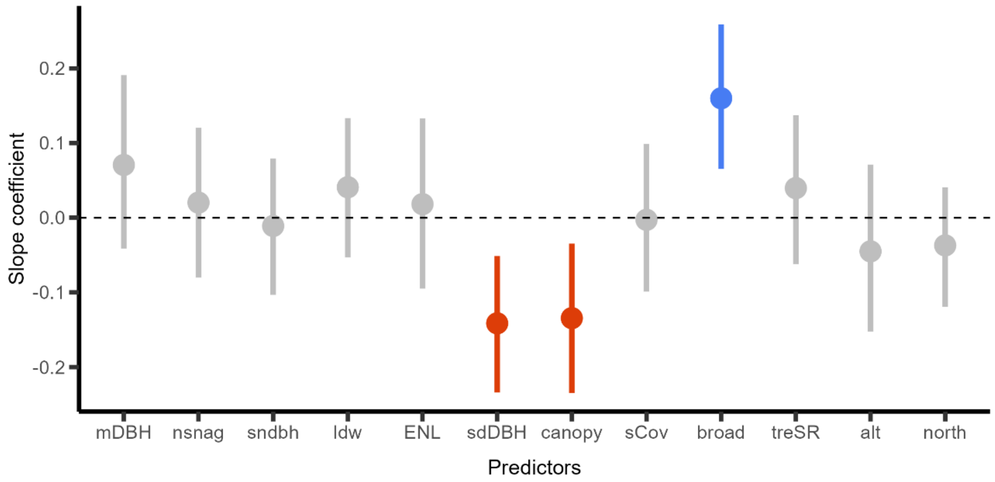

Supplement: Supplementary file 5 — Appendix S5. [file EAP-36-e70198-s006.pdf]
