# Supplementary material for: Disentangling the effects of multifunctional forestry practices on the abundances of birds and their invertebrate prey
Source: Ecol Appl. 2026 Mar 8;36(2):e70198. doi: 10.1002/eap.70198 (PMC12967705; doi:10.1002/eap.70198)
Supplement: Supplementary file 6 — Appendix S6. [file EAP-36-e70198-s007.pdf]

Appendix S6

Summary of invertebrate abundance models

Journal: Ecological Applications

Title: Disentangling the effects of multifunctional forestry practices on the abundances of birds and their invertebrate prey

Authors: João Manuel Cordeiro Pereira, Sara Klingenfuß, Marco Basile, Julian Frey, Grzegorz Mikusiński, Ilse Storch

Table S1: Details of abundance models for invertebrate groups, namely: the assumed distribution for invertebrate abundances (negative binomial, NB, or Poisson with an observation-level random effect, OLRE), the number of predictors included (specified for both a full and a reduced model following variable selection, if applicable), the variation and standard error of Expected Log Predictive Density (Δ ELPD) from the full to the reduced model (if applicable), a Bayesian R^2 and the posterior predictive probability. Also shown is the trapping method for each group (PT: pitfall traps; FIT: flight interception traps), the number of plots where trapping took place (No. plots), and the total number of specimens captured over all plots (No. specimens).

**Prey group**	**Method**	**No. plots**	**No. specimens**	**Model**	**No. predictors**	**Δ ELPD**	**Bayes R^2**	**Posterior predictive p**
Class **Gastropoda**<br>(snails and slugs)	PT	66	755	Poisson<br>(OLRE)	Full: 10<br>Reduced: 6	+ 6.9<br>(SE: 3.7)	89.1 %	0.495
Class Arachnida, Order **Opiliones**<br>(harvestmen)	PT	66	392	NB	Full: 10<br>Reduced: 6	+ 4.4<br>(SE: 0.7)	19.9 %	0.414
Class Arachnida, Order **Araneae**<br>(spiders)	PT	66	3,604	NB	Full: 10<br>Reduced: 6	+ 5.6<br>(SE: 1.2)	26.4 %	0.272
Class **Chilopoda**<br>(centipedes)	PT	66	178	NB	Full: 10<br>Reduced: 6	+ 5.1<br>(SE: 0.7)	17.5 %	0.547
Class **Diplopoda**<br>(milipedes)	PT	66	1,547	NB	Full: 10<br>Reduced: 6	+ 4.2<br>(SE: 1.4)	24.7 %	0.168

Class Malacostraca, Order **Isopoda** (woodlice)	PT	66	1,509	NB	Full: 10 Reduced: 6	+ 3.8 (SE: 2.7)	35.3 %	0.470
Class Entognatha, Subcl. **Collembola** (springtails)	PT	66	5,955	NB	Full: 10 Reduced: 6	+ 6.7 (SE: 2.1)	37.5 %	0.493
Class Insecta, Order **Hymenoptera** (largely ants, family Formicidae)	PT	66	13,135	NB	Full: 9 Reduced: 6	+ 6.0 (SE: 2.6)	23.9 %	0.275
Class Insecta, Order **Diptera** (flies and mosquitoes)	PT	66	4,077	Poisson (OLRE)	Full: 10 Reduced: 6	+ 3.6 (SE: 4.6)	98.7 %	0.491
Class Insecta, Order **Coleoptera** (beetles)	PT	66	9,645	NB	Full: 12 Reduced: 6	+ 4.5 (SE: 3.1)	38.9 %	0.631
Class Arachnida, Order **Araneae** (spiders)	FIT	125	2,180	NB	9	NA	33.6 %	0.511
Class Entognatha, Subcl. **Collembola** (springtails)	FIT	125	3,746	Poisson (OLRE)	9	NA	98.9 %	0.508
Class Insecta, Order **Psocoptera** (barklice)	FIT	125	3,204	Poisson (OLRE)	9	NA	96.5 %	0.503
Class Insecta, Order Hemiptera, Suborder **Auchenorrhyncha** (cicadas and leafhoppers)	FIT	125	1,680	Poisson (OLRE)	9	NA	96.8 %	0.511
Class Insecta, Order Hemiptera, Suborder **Sternorrhyncha** (aphids, whiteflies and scale insects)	FIT	125	23,420	NB	9	NA	35.9 %	0.245
Class Insecta, Order Hemiptera, Suborder **Heteroptera** (typical bugs)	FIT	125	961	NB	9	NA	47.1 %	0.463
Class Insecta, Order **Lepidoptera** (moths and butterflies)	FIT	125	1,231	Poisson (OLRE)	9	NA	87.7%	0.497

Class Insecta, Order **Diptera** (flies and mosquitoes)	FIT	125	37,367	NB	12	NA	28.5 %	0.272
Class Insecta, Order **Hymenoptera** (bees and wasps)	FIT	125	6,357	Poisson (OLRE)	12	NA	96.1 %	0.489
Class Insecta, Order **Coleoptera** (beetles)	FIT	125	34,133	Poisson (OLRE)	12	NA	99.2 %	0.516
