# Supplementary material for: Disentangling the effects of multifunctional forestry practices on the abundances of birds and their invertebrate prey
Source: Ecol Appl. 2026 Mar 8;36(2):e70198. doi: 10.1002/eap.70198 (PMC12967705; doi:10.1002/eap.70198)
Supplement: Supplementary file 7 — Appendix S7. [file EAP-36-e70198-s004.pdf]

Appendix S7

Path diagrams for Structural Equation Models (SEMs)

Journal: Ecological Applications

Title: Disentangling the effects of multifunctional forestry practices on the abundances of birds and their invertebrate prey

Authors: João Manuel Cordeiro Pereira, Sara Klingenfuß, Marco Basile, Julian Frey, Grzegorz Mikusiński, Ilse Storch

Figure S1: Full path diagrams for Structural Equation Models (SEMs), showing all significant paths (unidirectional arrows) and added free correlations (bidirectional arrows with dashed lines). Path colours reflects whether the relationship is negative (red) or positive (blue). For each variable entered as a response in the SEM, the R^2 value is shown below the variable name. Next to each path, the unstandardized coefficient is shown, followed by its significance level (* $p < .05$, ** $p < .01$, *** $p < .001$). **(a)** global SEM for the ground-foraging guild and epigeal invertebrates (n = 57, data from 2020); **(b)** specific SEM for small ground-foraging birds and epigeal spiders (n = 57, data from 2020); **(c)** global SEM for foliage-gleaning and bark-foraging guilds, and invertebrates from flight interception traps (n = 97, data from 2017).

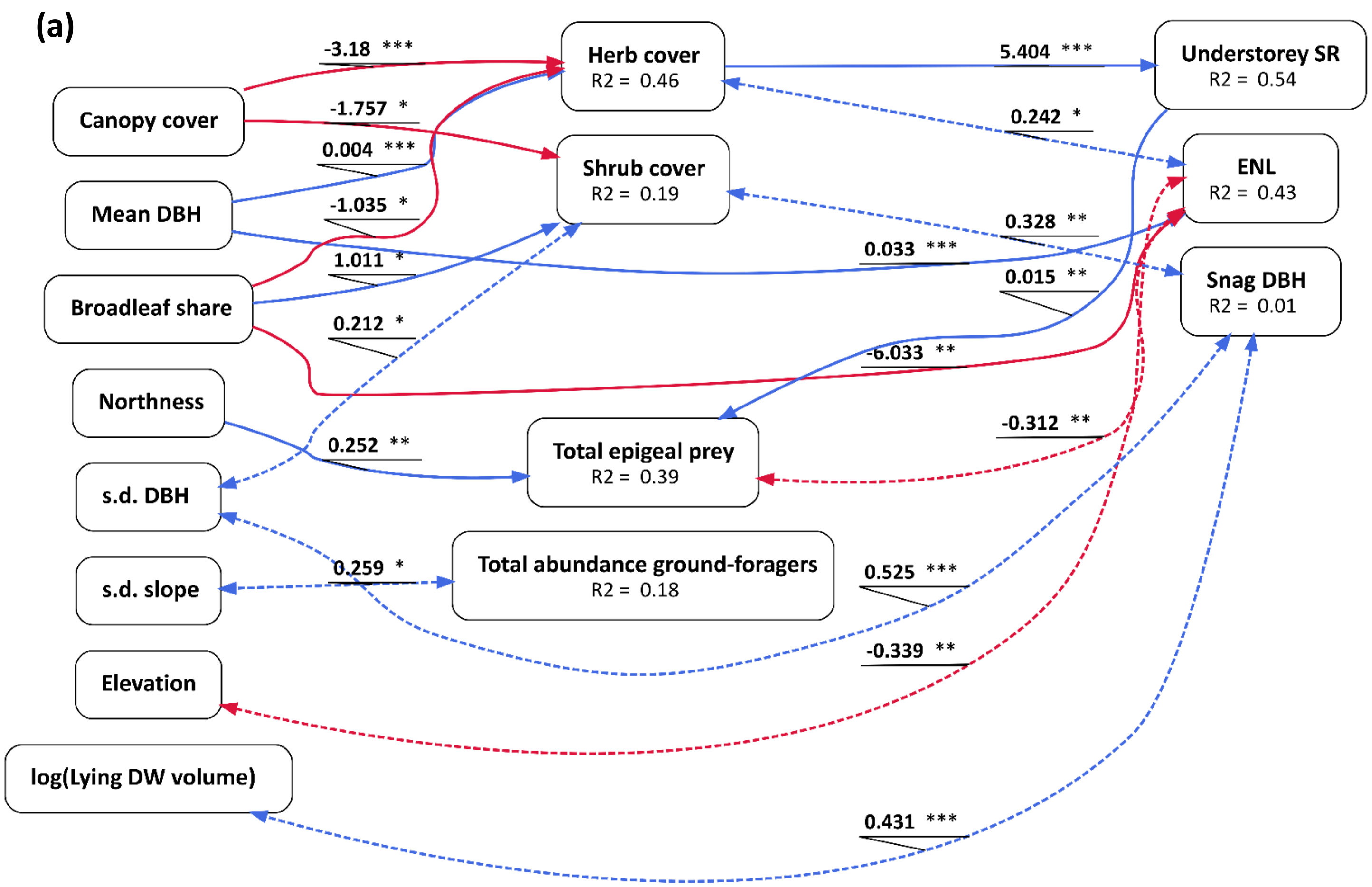
(a)
Herb cover
R2 = 0.46
Understorey SR
R2 = 0.54
Canopy cover
Shrub cover
R2 = 0.19
ENL
R2 = 0.43
Mean DBH
Snag DBH
R2 = 0.01
Broadleaf share
Northness
Total epigeal prey
R2 = 0.39
s.d. DBH
Total abundance ground-foragers
R2 = 0.18
s.d. slope
Elevation
log(Lying DW volume)
-3.18 ***
-1.757 *
0.004 ***
-1.035 *
1.011 *
0.212 *
0.252 **
0.259 *
5.404 ***
0.242 *
0.328 **
0.033 ***
0.015 **
-6.033 **
-0.312 **
0.525 ***
-0.339 **
0.431 ***

(b)

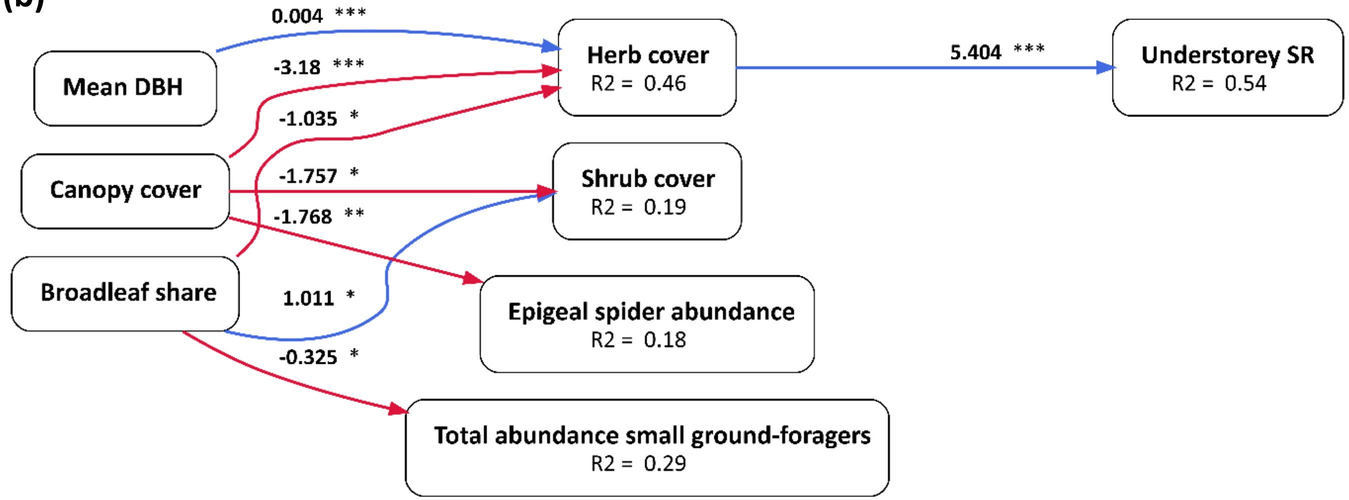
0.004 ***
-3.18 ***
-1.035 *
-1.757 *
-1.768 **
1.011 *
-0.325 *
5.404 ***
Mean DBH
Canopy cover
Broadleaf share
Herb cover
R2 = 0.46
Shrub cover
R2 = 0.19
Epigeal spider abundance
R2 = 0.18
Total abundance small ground-foragers
R2 = 0.29
Understorey SR
R2 = 0.54

(c)

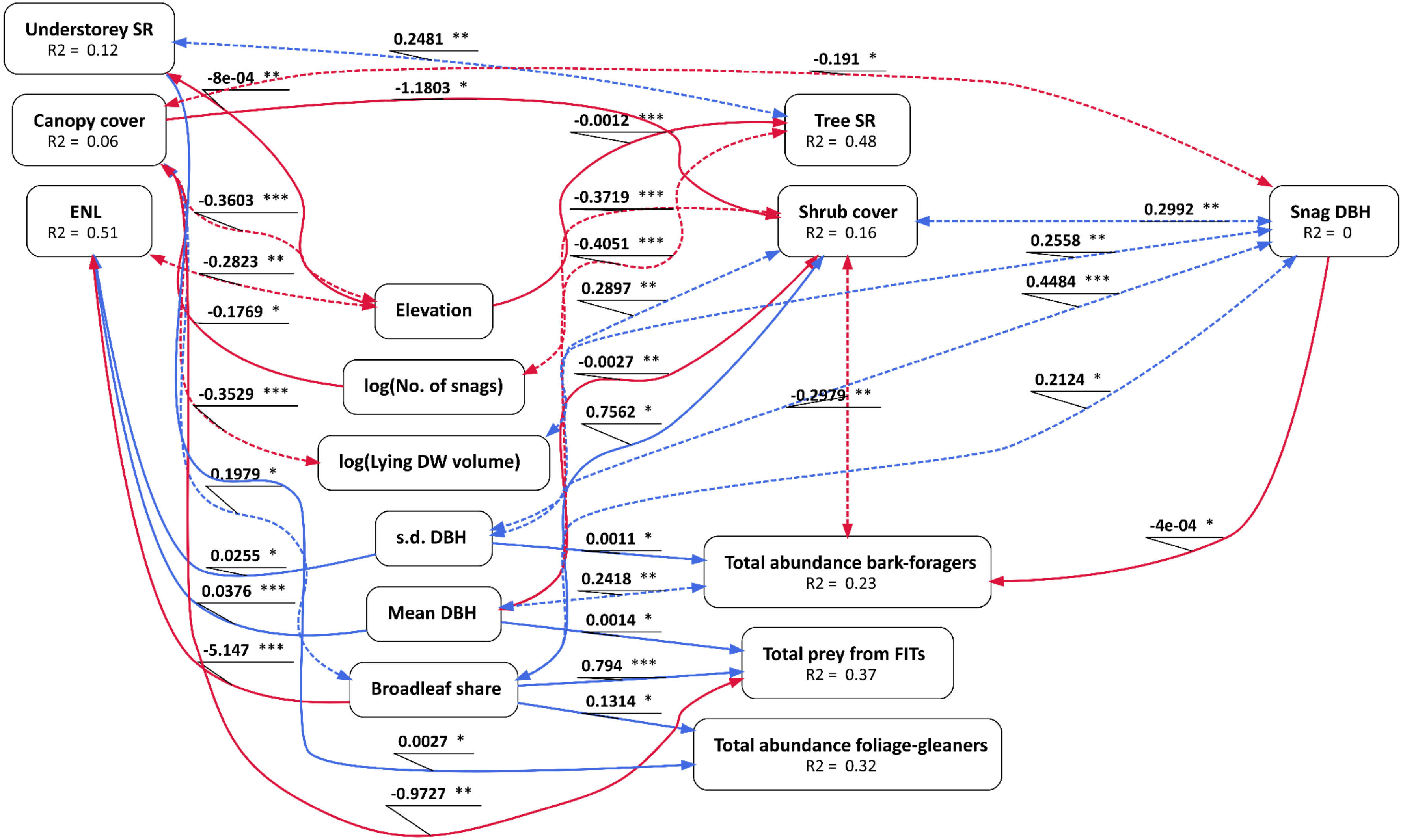

Understorey SR
R2 = 0.12
Canopy cover
R2 = 0.06
ENL
R2 = 0.51
Tree SR
R2 = 0.48
Shrub cover
R2 = 0.16
Snag DBH
R2 = 0
Elevation
log(No. of snags)
log(Lying DW volume)
s.d. DBH
Mean DBH
Broadleaf share
Total abundance bark-foragers
R2 = 0.23
Total prey from FITs
R2 = 0.37
Total abundance foliage-gleaners
R2 = 0.32
0.2481 **
-0.191 *
-8e-04 **
-1.1803 *
-0.0012 ***
-0.3603 ***
-0.3719 ***
0.2992 **
-0.4051 ***
0.2558 **
-0.2823 **
0.2897 **
0.4484 ***
-0.1769 *
-0.0027 **
0.2124 *
-0.3529 ***
-0.2979 **
0.7562 *
0.1979 *
-4e-04 *
0.0011 *
0.0255 *
0.2418 **
0.0376 ***
0.0014 *
-5.147 ***
0.794 ***
0.1314 *
0.0027 *
-0.9727 **
